# A novel rubber tree PR-10 protein involved in host-defense response against the white root rot fungus *Rigidoporus microporus*

**DOI:** 10.1186/s12870-023-04149-3

**Published:** 2023-03-22

**Authors:** Rawit Longsaward, Ashara Pengnoo, Panida Kongsawadworakul, Unchera Viboonjun

**Affiliations:** 1 Department of Plant Science, Faculty of Science, Mahidol University, Bangkok, 10400 Thailand; 2 Agricultural Innovation and Management Division, Faculty of Natural Resources, Prince of Songkla University, Hat Yai Campus, Songkhla, 90110 Thailand; 3 Natural Biological Control Research Center, National Research Council of Thailand, 196 Phahonyothin Road, Lat Yao, Chatuchak, Bangkok, 10900 Thailand

**Keywords:** Rubber tree, *Hevea brasiliensis*, *Rigidoporus microporus*, Proteomics, PR-10

## Abstract

**Background:**

White root rot disease in rubber trees, caused by the pathogenic fungi *Rigidoporus microporus*, is currently considered a major problem in rubber tree plantations worldwide. Only a few reports have mentioned the response of rubber trees occurring at the non-infection sites, which is crucial for the disease understanding and protecting the yield losses.

**Results:**

Through a comparative proteomic study using the two-dimensional polyacrylamide gel electrophoresis (2D-PAGE) technique, the present study reveals some distal-responsive proteins in rubber tree leaves during the plant-fungal pathogen interaction. From a total of 12 selected differentially expressed protein spots, several defense-related proteins such as molecular chaperones and ROS-detoxifying enzymes were identified. The expression of 6 candidate proteins was investigated at the transcript level by Reverse Transcription Quantitative PCR (RT-qPCR). In silico, a highly-expressed uncharacterized protein LOC110648447 found in rubber trees was predicted to be a protein in the pathogenesis-related protein 10 (PR-10) class. In silico promoter analysis and structural-related characterization of this novel PR-10 protein suggest that it plays a potential role in defending rubber trees against *R. microporus* infection. The promoter contains WRKY-, MYB-, and other defense-related *cis*-acting elements. The structural model of the novel PR-10 protein predicted by I-TASSER showed a topology of the Bet v 1 protein family, including a conserved active site and a ligand-binding hydrophobic cavity.

**Conclusions:**

A novel protein in the PR-10 group increased sharply in rubber tree leaves during interaction with the white root rot pathogen, potentially contributing to host defense. The results of this study provide information useful for white root rot disease management of rubber trees in the future.

**Supplementary Information:**

The online version contains supplementary material available at 10.1186/s12870-023-04149-3.

## Background

Natural rubber obtained from rubber trees (*Hevea brasiliensis* (Willd. ex A.Juss.) Müll.Arg.) is an essential and critical raw material for many specialized products, especially for medical devices, aircraft parts, and car tires. The rubber tree has become an economically important crop in many countries, especially in Southeast Asia [6, 22, 64]. Yet numerous factors, both internal (such as rubber tree clone) and external, influence the yield gained from natural rubber production. In rubber-producing countries in tropical areas, severe flooding events and heavy rainfall caused by monsoons increase the risk of invasion by soil-borne fungal pathogens [48, 81]. Root diseases caused by soil-borne fungi can lead to serious pathogenic and economic problems, as they can affect water and nutrient uptake, and hence reduce the growth rate and yield of natural rubber production.

One of the most destructive root diseases in rubber plantations is white root rot, caused by the pathogenic fungi *Rigidoporus microporus* (Fr.) Overeem [36, 89]. It has been suggested that this disease is the main cause of the current rubber tree crisis in many rubber-producing countries, including Thailand, Malaysia, Indonesia, Sri Lanka, Nigeria, and Cote d’Ivoire [7]. To date, there is no rubber tree clone that is completely resistant against this root disease, and new pathogenic fungus strains can emerge with increased aggressiveness, enabling them to overcome evolved host resistances. Trace amounts of fungi can persist in the soil for long periods of time [97] and continuously infect other living roots and/or wood debris in the plantation area. The disease is known to spread from one plant to another through rhizomorphs [84]. The well-developed rhizomorph of this species is highly influenced by the tropical environment, as the fungi favor moderate to high soil moisture, warm temperatures, compacted soil with poor drainage, as well as other factors that promote the pathogen’s spread [7].

The aggressiveness of *R. microporus* in rubber trees has been a crucial issue for developing effective disease management strategies. The roots of the rubber tree turn creamier and softer after being infected by *R. microporus*. The fungus has the ability to release cell wall-degrading enzymes into rubber tree roots, leading to defects in host cell lignification, which is beneficial for fungal penetration [64]. The defensive latex of the rubber tree did not have any negative effects on *R. microporus* and, conversely, the fungi displayed fast growth due to their various gene activities, possibly related to latex degradation [62]. The fungi also increase the activities of amino acid and carbohydrate metabolism enzymes, as well as the production of many acidic compounds, which promotes their pathogenicity during penetration and colonization of rubber tree roots [20]. The symptoms of infected rubber trees initially develop and progress in the roots below ground, whereas the aboveground parts are almost identical to those of healthy trees [18]. As a consequence, it is difficult to identify infected trees during the early stage of root disease because noticeable symptoms on aboveground parts are only displayed once the fungus has destroyed almost all of the root system [61]. Normally, initial aboveground symptoms of infected plants include the discoloration of leaves from deep green to yellowish-brown, early flowering, and off-season fruiting. The severest events result in the death of the entire rubber tree, in which all of the leaves fall before the appearance of *R. microporus* basidiocarps on the bark of the lower tree trunk [64].

To explore further possibilities for white root rot disease management, such as the breeding of resistant genes in rubber trees, a more thorough understanding of molecular interactions between rubber trees and *R. microposus* fungi is needed. The response of rubber trees to white root rot disease is systemically linked from the infected root to other distal parts. Moreover, the molecular mechanism of rubber trees that develop an early response against *R. microporus* infection is known to biochemically respond by stimulation of key enzymes in biosynthesis, such as cell wall lignification enzymes in the infected roots [82]. Previous research has already reported the plant enzyme activities involved in degrading structural polymers of fungal mycelia walls as a host mechanism at the site of infection [67]. Rubber trees have the potential to produce antimicrobial compounds such as coumarins, flavonoids, and triterpenes that are able to inhibit microbial penetration [33].

The advancement of technology has allowed for investigation of responsive genes and proteins that are expressed in rubber trees after infection by *R. microporus*, which could potentially fulfill missing gaps and reveal candidate genes and proteins involved in the systemic response to disease, such as the pathogenesis-related (PR) proteins expressed in the stem [63]. Leaves are another organ of interest that are not only a center of energy production, but can also perceive and amplify PAMP-triggered immunity (PTI) signals in plants [23], [35], [59]. Here, we performed a comparative leaf proteome and we reveal the noteworthy role of an uncharacterized protein LOC110648447 as well as other defense-related proteins in the distal response of rubber trees infected at the root by *R. microporus*. We further analyze the expression of these selected proteins at the transcript level and provide an in silico functional prediction of the uncharacterized protein identified in this study. The expression levels of these defense-related candidates in rubber tree leaves will contribute to our understanding of the interaction between plants and fungi, and benefit white root rot disease management in the future.

## Results

### Comparative rubber tree leaf proteome reveals some differentially expressed proteins after *R. microporus* inoculation

In this study, proteomic analysis was employed to investigate how rubber trees respond to the presence of *R. microporus*, the white root rot pathogenic fungus. Leaf protein samples were extracted from rubber seedlings at 10, 30, and 50 days after inoculation (DAI). The comparative leaf proteome between *R. microporus*-inoculated and mock-inoculated (control) seedlings at each time-point were then assessed by one-dimensional and two-dimensional polyacrylamide gel electrophoresis (1D- and 2D-PAGE).

**Fig. 1:**
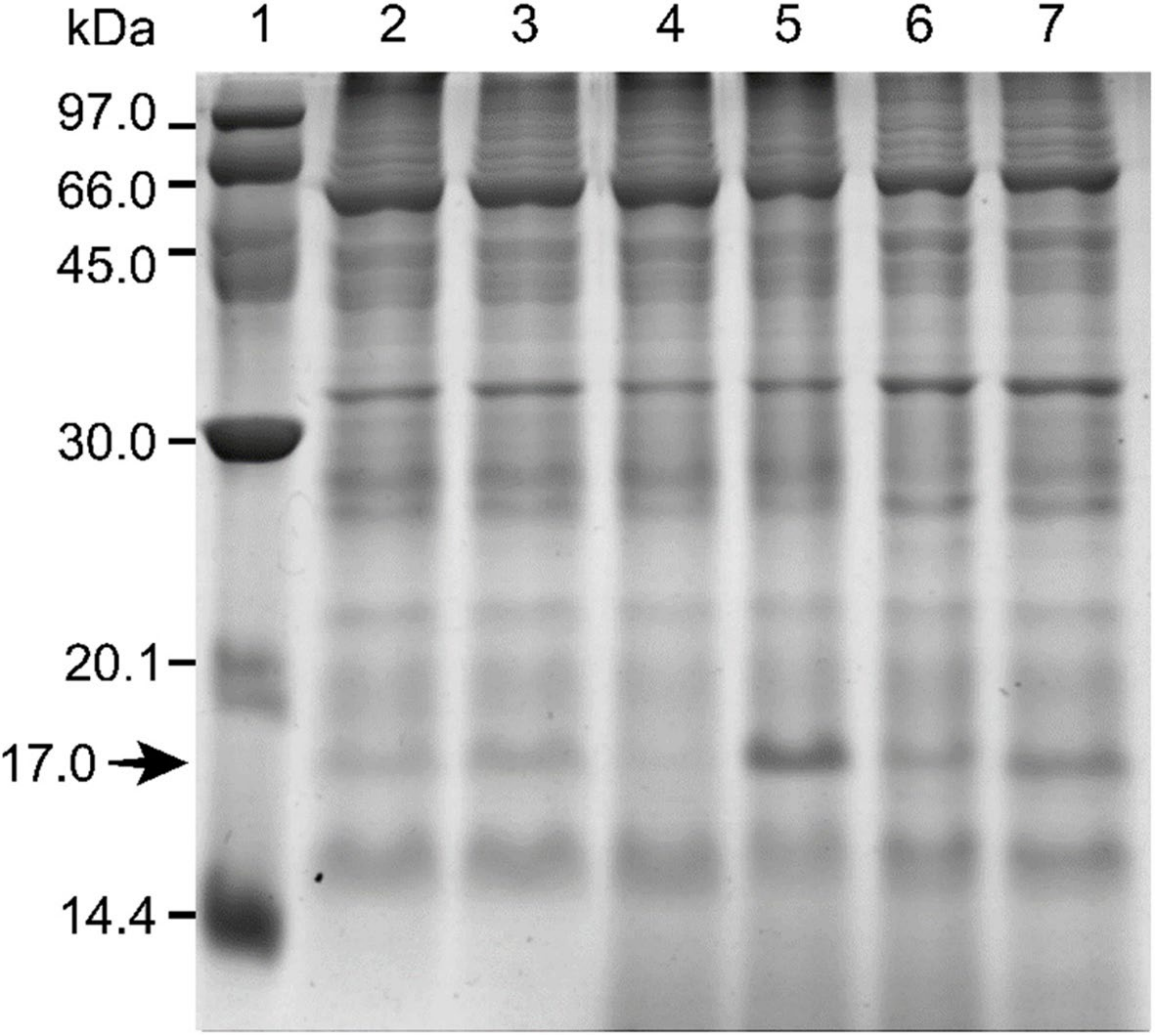
Comparison of the protein patterns of rubber tree leaves after *R. microporus* inoculation by SDS-PAGE with Coomassie brilliant blue (CBB) dye. Lane 1: protein marker; Lanes 2, 4, 6: mock inoculation at 10, 30, and 50 days after inoculation (DAI), respectively; Lanes 3, 5, 7: *R. microporus* inoculation at 10, 30, and 50 DAI, respectively

According to the 1D-PAGE results, the protein patterns in inoculated plants compared with mock-inoculated ones were largely similar in their intensity and mass separation (Fig. 1, Supplementary Fig. 2). Differences were observed in the protein fraction at approximately 17 kDa, which exhibited distinctly higher accumulation in inoculated samples compared to control samples at 30 DAI.

**Fig. 2:**
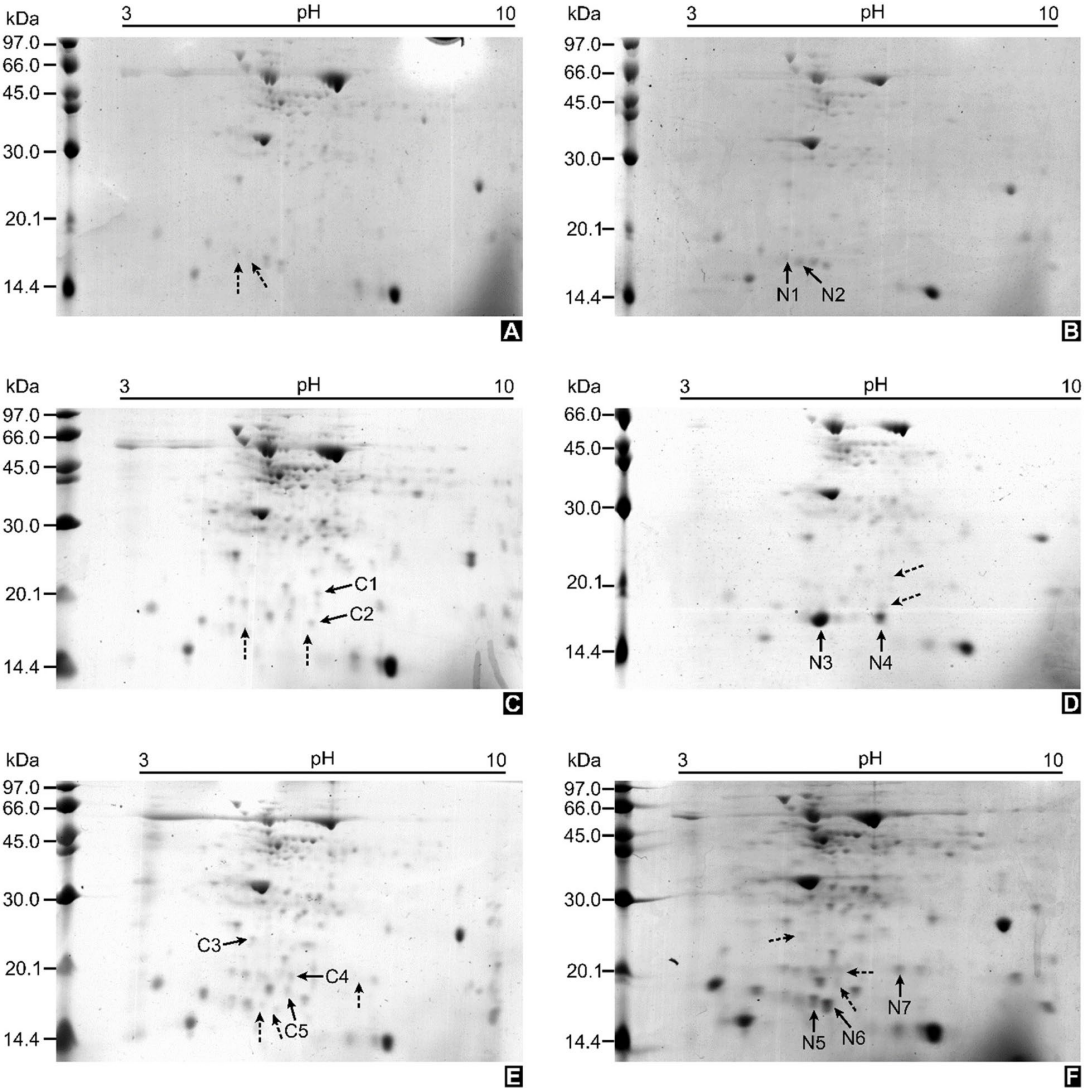
Representative gel profiles from two-dimensional polyacrylamide gel electrophoresis (2D-PAGE) of rubber tree leaf proteins. Samples of mock-inoculation at 10 days after inoculation (DAI) (**A**), 30 DAI (**C**), 50 DAI (**E**); Samples of *R. microporus*- inoculation at 10 DAI (**B**), 30 DAI (**D**), 50 DAI (**F**)

For increased resolution, 2D-PAGE was performed as a gel-based proteomic approach. The protein spots were detected by Coomassie blue G staining. The protein patterns of inoculated plants and mock-inoculated ones were found to be widely distributed in the 2D gel, with various pI values and molecular weights (Fig. 2, Supplementary Fig. 3). Similar proteins were found in both conditions, as was observed in 1D-PAGE. Comparative image analysis revealed a total of 12 differentially expressed protein spots of interest.

The 17-kDa protein band from 1D-PAGE (Fig. 1), as well as the 12 differentially expressed protein spots from 2D-PAGE (Fig. 2), were excised and successfully identified by LC–MS/MS analysis (Table 1; Supplementary Table 1). Four of the down-regulated proteins were identified as stress-related proteins in rubber trees, including a 17.3-kDa class I heat shock protein-like protein (spot C1), a superoxide dismutase [Cu–Zn]-like protein (spot C2), an ATP synthase subunit D mitochondrial-like protein (spot C3), and a universal stress PHOS34 protein (spot C4). The last down-regulated spot (spot C5) was identified as a very short fragment of putative cytochrome c oxidase subunit II PS17 in *Pinus strobus*. Interestingly, most of the up-regulated protein spots (spots N1, N2, N3, N5, N6) and the 17-kDa protein band from 1D-PAGE were identified as the uncharacterized protein LOC110648447, of which 3 isoforms are available in the NCBI database (Supplementary Table 1). Spots N4 and N7 were identified as an aminotran_3 domain-containing protein of a carnivorous pitcher plant (*Cephalotus follicularis*) and a metalloendoproteinase 5-MMP-like protein of rubber tree, respectively.

**Table 1:** Identification results of the selected 1D-PAGE band and 2D-PAGE spots by ESI-QUAD-TOF mass spectrometry (MS/MS) analysis

Spot ID	Protein name	GenBank Accession no	Theoretical pI/MW (kDa)	Observed pI/MW (kDa)	Sequence coverage (%)	emPAI	Fold change
17 kDa protein band from 1D-PAGE	Uncharacterized protein LOC110648447 [*H. brasiliensis*]	XP_021658364.1	5.2/17.0	-/17.0	56.9	5.25	n/a
		XP_021658366.1	5.1/16.6		62.0	3.96	
	Oxygen-evolving enhancer protein 3–2, chloroplastic-like [*H. brasiliensis*]	XP_021639605.1	9.5/24.4		29.9	1.51	
C1	17.3 kDa class I heat shock protein-like [*H. brasiliensis*]	XP_021675560.1	6.0/18.4	6.5/22.0	8.0	0.22	-1.308
C2	Superoxide dismutase [Cu–Zn]-like [*H. brasiliensis*]	XP_021640614.1	5.6/15.4	6.4/18.0	8.0	0.26	-1.779
C3	ATP synthase subunit D, mitochondrial-like [*H. brasiliensis*]	XP_021666672.1	5.1/19.8	5.3/26.5	29.0	3.20	-2.597
C4	Universal stress protein PHOS34 [*H. brasiliensis*]	XP_021668720.1	5.7/18.3	6.0/20.0	18.0	0.47	-1.847
C5	Putative cytochrome c oxidase subunit II PS17 (Fragments) [*Pinus strobus*]	PS17_PINST	9.6/17.1	5.9/18.5	50.0	3.44	-8.712
N1	Uncharacterized protein LOC110648447 [*H. brasiliensis*]	XP_021658364.1	5.2/17.0	4.7/17.7	32.5	1.29	Undetectable in control treatment
		XP_021658365.1	5.3/16.7		35.0	1.87	
		XP_021658366.1	5.1/16.6		35.0	1.87	
N2	Uncharacterized protein LOC110648447 [*H. brasiliensis*]	XP_021658364.1	5.2/17.0	4.9/17.3	60.0	4.25	Undetectable in control treatment
		XP_021658366.1	5.1/16.6		62.3	2.55	
N3	Uncharacterized protein LOC110648447 [*H. brasiliensis*]	XP_021658364.1	5.2/17.0	5.2/16.3	68.2	5.46	Undetectable in control treatment
		XP_021658365.1	5.3/16.7		82.1	4.42	
		XP_021658366.1	5.1/16.6		81.0	5.69	
N4	Aminotran_3 domain-containing protein [*Cephalotus follicularis*]	GAV65601.1	6.4/51.0	6.3/17.0	1.0	0.07	Undetectable in control treatment
N5	Uncharacterized protein LOC110648447 [*H. brasiliensis*]	XP_021658364.1	5.2/17.0	5.5/17.3	40.0	3.22	+ 2.959
		XP_021658365.1	5.3/16.7		24.5	0.87	
		XP_021658366.1	5.1/16.6		24.5	0.87	
N6	Uncharacterized protein LOC110648447 isoform X1 [*H. brasiliensis*]	XP_021658364.1	5.2/17.0	5.7/17.3	7.0	0.22	+ 2.913
N7	Metalloendoproteinase 5-MMP-like [*H. brasiliensis*]	XP_021637894.1	7.0/34.4	7.0/20.5	6.0	0.53	Undetectable in control treatment

### Relative gene expression of selected *R. microporus* responsive proteins in rubber tree leaves by RT-qPCR

The gene expression levels of 6 selected identified proteins were examined by RT-qPCR in order to verify the correlation between protein and gene expression. The specific primer pairs for each gene were designed and validated. The expression level during each time-point of the inoculated samples was calculated relatively to the mock-inoculated samples at 30 DAI, which is considered the control for RT-qPCR. For the protein showing three isoforms, the uncharacterized protein LOC110648447, only one specific primer pair corresponding to isoform 1 (XP_021658364.1) was obtained. All of the tested primer pairs for individual candidates showed high efficiency, ranging from 90.11 to 116.46 (Table 2). The specificity of all transcripts being amplified as a single amplicon was assessed using melting curve analysis.

**Table 2:** Descriptions of candidate genes and their primer characteristics for RT-qPCR

Candidate protein/GenBank Accession no	Forward primer (5' to 3')	Reverse primer (5' to 3')	Amplicon size (bp)	T_a_ (^o^C)	Primer efficiency	R^2^
Uncharacterized protein LOC110648447 isoform X1 (XM_021802672.1)	CTTATCACTGAAAACAGTCGGGG	ATCCATTCCACCCATTTGCCA	108	61	90.11	0.9318
Uncharacterized protein LOC110648447 isoform X2 (XM_021802673.1)	CGCAAAGTCGAGTATGGCCC	ACAGCTTGGAAGTTGTTGACATC	142	61	108.25	0.7156
Uncharacterized protein LOC110648447 isoform X3 (XM_021802674.1)						
Oxygen-evolving enhancer 3–2 (XM_021783913.1)	CAATTCAGTGGCTCAACCCG	CAGGCTCAGCAGAAGAAGGT	115	60	113.93	0.8514
17.3 kDa class I heat shock protein-like (XM_021819868.1)	AGGCAAGCATGGAGAATGGA	TCAACCGGAGATCTCAATGGC	92	60	114.35	0.8220
Superoxide dismutase [Cu–Zn]-like (XM_021784922.1)	AAATCACTGCCTTCCCCTGAC	ATTGTCCCGCTAACACCCTC	161	59	115.10	0.9128
Universal stress protein PHOS34 (XM_021813028.1)	TAGCTCTGAAGTTGCGGTCC	CTGCTGCAAGGCGAGAAATC	133	61	108.64	0.8771
Metalloendoproteinase 5-MMP-like (XM_021782202.1)	CAGTGGAAGGGCTGAGTAAGG	GAACTCCGCATCGTGGAATC	201	58	116.46	0.9373

Interestingly, comparative analysis between protein abundance and RT-qPCR expression profiles showed that most proteins and genes (5 of 6) displayed similar behaviors, confirming the abundance data observed through 2D-PAGE. The one exception was the metalloendoproteinase 5-MMP-like protein; a candidate gene from an up-regulated protein spot showed a significant decrease in gene expression at all 3 time-points after inoculation.

**Fig. 3:**
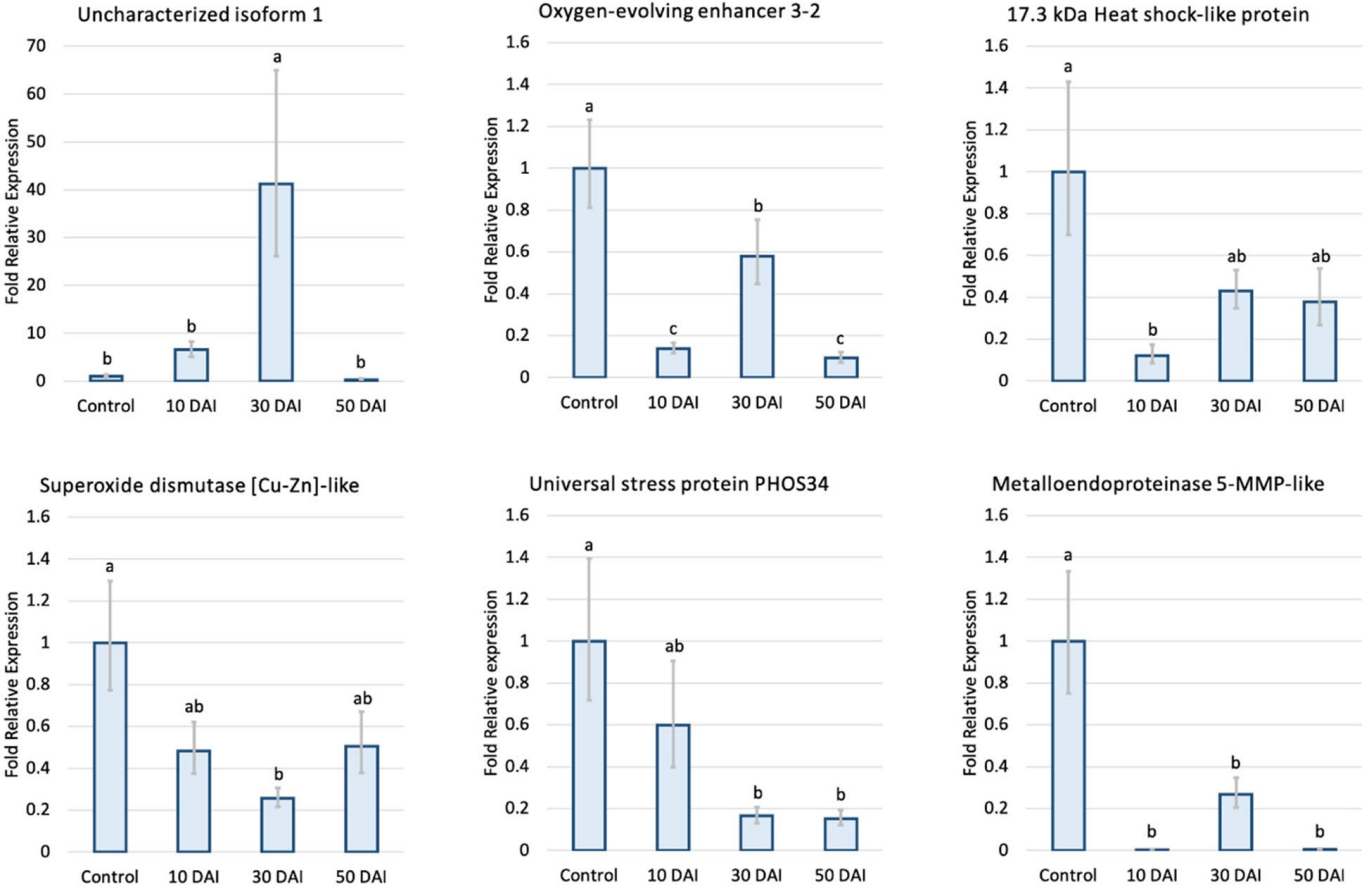
Fold relative expression of candidate genes in rubber tree leaves analyzed by RT-qPCR at 10, 30, and 50 days after *R. microporus* inoculation compared to mock-inoculation at 30 DAI (control). The expression levels of target genes were normalized with the UBC2a reference gene [46]. The data obtained from 3 biological and 9 technical replicates (*N* = 27) were analyzed for the fold relative expression, following the modified method described by Taylor et al. [90]. Error bars indicate one standard error. Statistical analysis was performed with an analysis of variance (ANOVA) and Tukey’s post hoc significance test at *p* < 0.05, groups with different letters are significantly different

Among the six candidate genes selected (Fig. 3), the uncharacterized protein LOC110648447 gene showed significantly higher expression (41-fold) at 30 DAI. The transcript of the 17.3-kDa class I heat shock protein-like gene was significantly down-regulated at 10 DAI compared to the control, whereas the transcripts of the superoxide dismutase [Cu–Zn]-like and universal stress PHOS34 protein genes decreased significantly at 30 DAI and 30 to 50 DAI, respectively. The oxygen-evolving enhancer 3–2, which is another candidate protein identified from the 17-kDa band from 1D-PAGE (Fig. 1, Table 1), showed significantly down-regulated gene expression, especially at 10 and 50 DAI.

### *Cis*-acting regulatory elements of uncharacterized protein LOC110648447

**Fig. 4:**
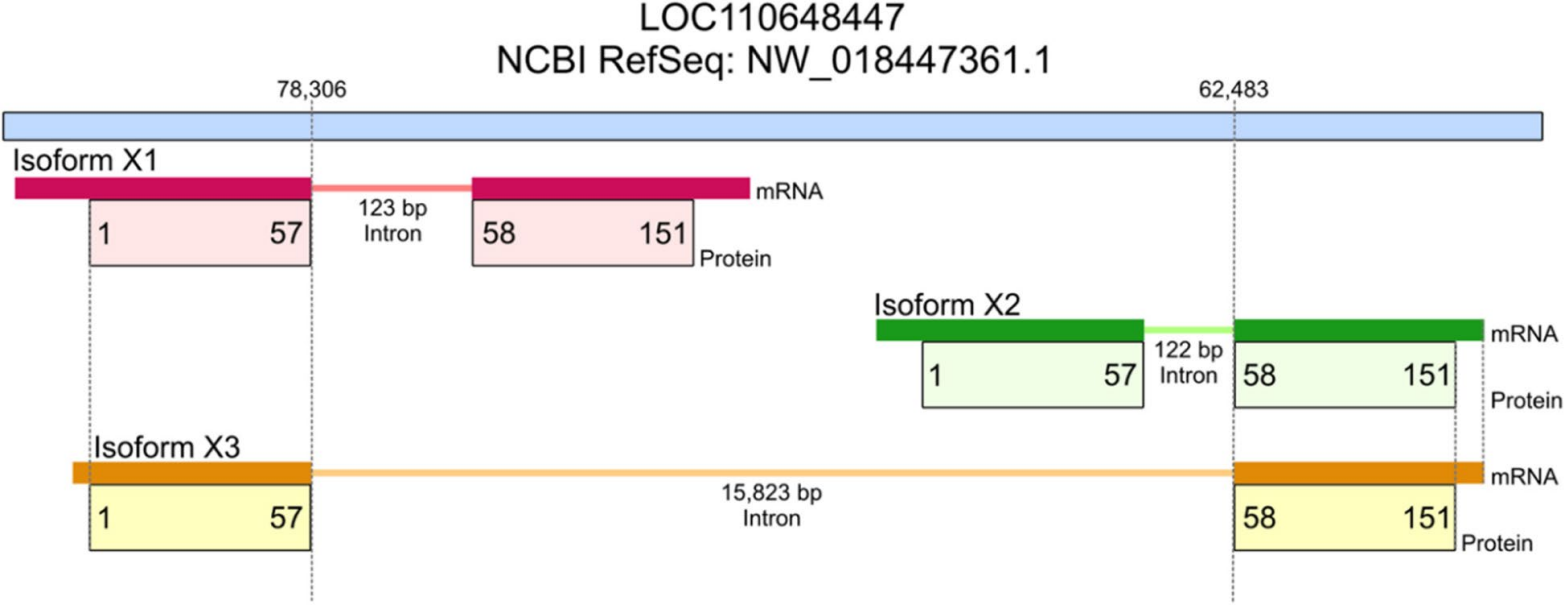
Graphical representation of genomic regions containing the uncharacterized protein LOC110648447 of rubber tree clone reyan7-33–97

The high up-regulation of the uncharacterized protein LOC110648447 (isoform X1, XP_021658364.1) in response to *R. microporus* infection suggests it plays an important role during plant-pathogenic fungi interactions. The NEWPLACE database was used to perform in silico analysis of putative *cis*-acting elements present on the 3000 bp upstream of the transcription start site in order to identify potential regulatory elements that respond to pathogens. The genomic reference sequence of the uncharacterized protein LOC110648447 (NW_018447361.1, Fig. 4) was obtained from the whole-genome shotgun contig LVXX01001672.1 of rubber tree clone Reyan7-33–97. However, the reference sequence contains only 137 bp of the upstream region for *cis*-acting element prediction (Supplementary Fig. 4).

The mRNA sequence encoding the uncharacterized protein LOC110648447 (isoform X1, XP_021658364.1) was homologous to the whole-genome shotgun contig MKXE01009552.1 from rubber tree clone RRIM600 (Supplementary Fig. 5), the same clone as the plant materials used in this study. The 137 bp of the upstream sequence of the transcription start site of this gene, obtained from two clones, Reyan7-33–97 and RRIM600, are identical. Thus, investigation of *cis*-acting elements using the genome sequence from rubber tree clone RRIM600 was then performed. The obtained *cis*-acting elements on the promoter region are listed (Supplementary Table 2; Supplementary Fig. 6), including developmental-regulated, tissue-specific, hormone-responsive, and stress-responsive elements. Some of these elements were previously reported to play a crucial role in regulating defensive genes of other plant species, as well as in response to drought stress (Table 3).

**Table 3:** Selected stress-related putative *cis*-regulatory elements found on the upstream region of the uncharacterized protein LOC110648447 genomic sequence of rubber tree clone RRIM600

Element	Sequence	Upstream site	Function	NEWPLACE database ID
ASF1MOTIFCAMV	TGACG	-405	Abiotic and biotic stress response with auxin and/or SA hormone	S000024
BIHDOS	TGTCA	-749 -1447 -2151	Disease response	S000498
GT1GMSCAM4	GAAAAA	-1699 -1805 -2098	Pathogen- and salt-induced, Fungal elicitor response element	S000453
MYB1AT	WAACCA	-879 -1266 -2488	MYB recognition site, drought stress regulation	S000408
MYCATERD1	CATGTG	-1161	MYC recognition site, specifically bind by NAC protein	S000413
PREATPRODH	ACTCAT	-2221	Pro- or hypoosmolarity-responsive element (PRE)	S000450
WBOXNTERF3	TGACY	-2224 -2959	W-box in wounding response	S000457
WRKY71OS	TGAC	-406 -2225 -2960	Binding site of rice WRKY71, W-box elements within the parsley *PR-10* genes	S000447
WBOXATNPR1	TTGAC	-406 -2225	W-box element within the Arabidopsis *NPR1* gene	S000390

### Classification of uncharacterized protein LOC110648447 based on amino acid sequences

**Fig. 5:**
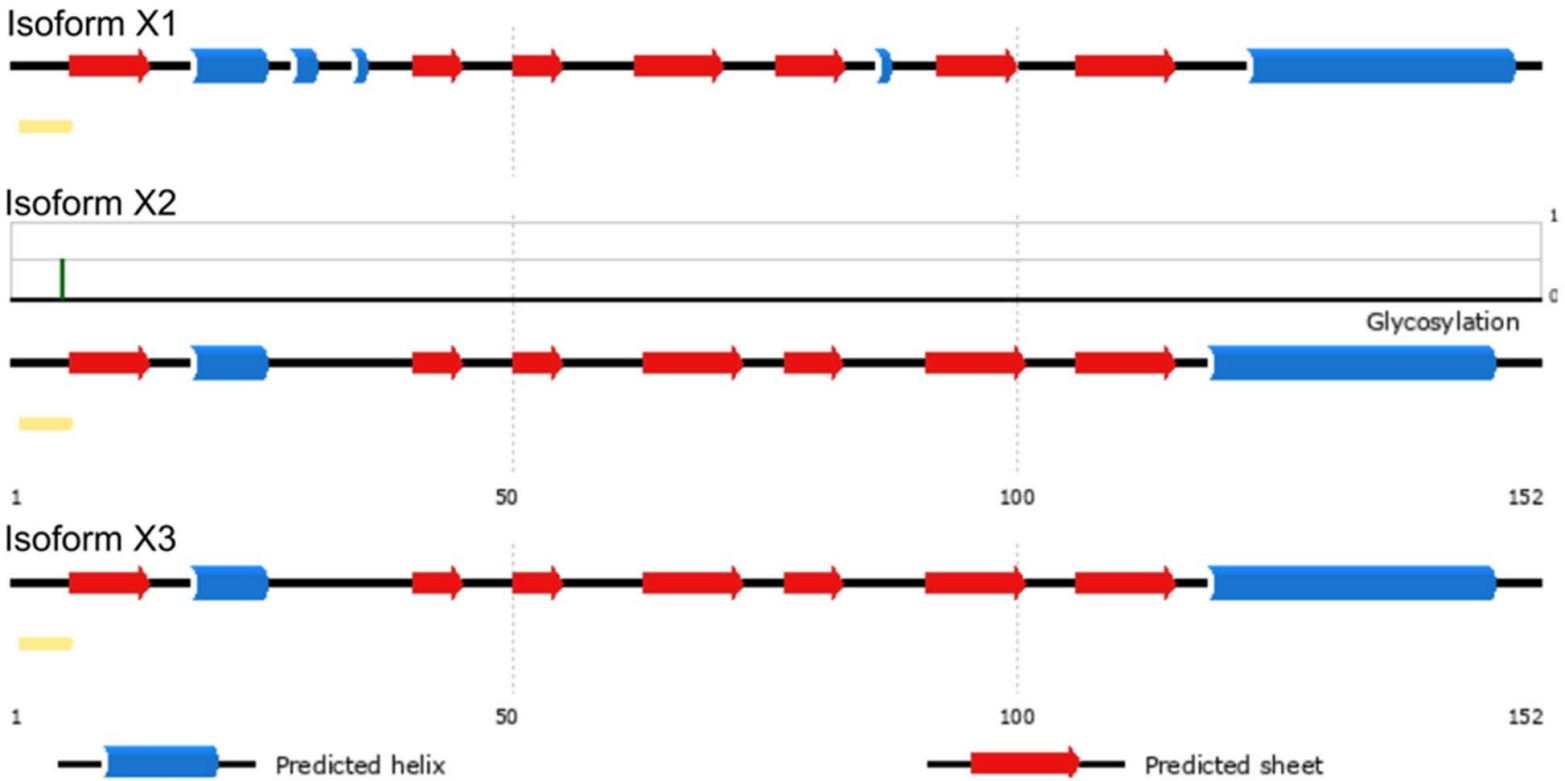
The predicted secondary structures of 3 isoforms of uncharacterized protein LOC110648447 by the PSIPRED server showing β-α_1_-β_6_ and C-terminal α-helix, a topology of the Bet v 1 protein superfamily. The predicted glycosylation site at residue Thr5 in isoform X2 is indicated with a green line (*P* = 0.5)

In order to predict its function, the amino acid sequences of uncharacterized protein LOC110648447 isoform X1 (XP_021658364.1), X2 (XP_021658365.1), and X3 (XP_021658366.1) were blasted against the NCBI and Uniprot databases. The best results were matched with the same group of uncharacterized proteins and hypothetical proteins in rubber trees with more than 80% identity. Some matched known proteins include those in the group of Bet v 1 superfamily with a maximum identity of 63.8% (Table 4). PSIPRED was used to look for secondary structures in deduced amino acid sequences of uncharacterized protein LOC110648447 isoforms X1, X2, and X3 (Supplementary Fig. 7). The obtained modeling structure suggests that the proteins contain β-α_1_-β_6_ and C-terminal α-helix, which corresponds to the topology of the Bet v 1 superfamily [70] (Fig. 5). Moreover, a residue for probable glycosylation was predicted at Thr5 of the uncharacterized protein LOC110648447 isoform X2 (*P* = 0.5).

**Table 4:** The percentage of sequence similarity between the three isoforms of the unidentified protein LOC110648447 and related known proteins in the Bet v 1 superfamily

Query	Percent coverage/Percent identity				
	Bet v 1-domain containing protein [*H. brasiliensis*] (A0A6A6KMV5)	Uncharacterized protein [*Manihot esculenta*] (XP_021614465.1)	MLP-like protein 423 [*H. brasiliensis*] (XP_021679695.1)	Bet v 1 domain-containing protein [*Artemisia annua*] (PWA67768.1)	Bet v 1 domain-containing protein [*Cephalotus follicularis*] (GAV74530.1)
Uncharacterized protein LOC110648447 isoform X1 [*H. brasiliensis*]	100/63.8	98.00/47.02	97.00/42.95	90.00/33.10	72.00/36.67
Uncharacterized protein LOC110648447 isoform X2 [*H. brasiliensis*]	100/63.8	99.00/48.68	100.00/46.15	90.00/35.14	93.00/32.89
Uncharacterized protein LOC110648447 isoform X3 [*H. brasiliensis*]	100/62.5	99.00/48.03	100.00/44.87	90.00/35.81	90.00/34.69

### In-silico prediction of the 3D structure and interactive features of uncharacterized protein LOC110648447 by I-TASSER

**Fig. 6:**
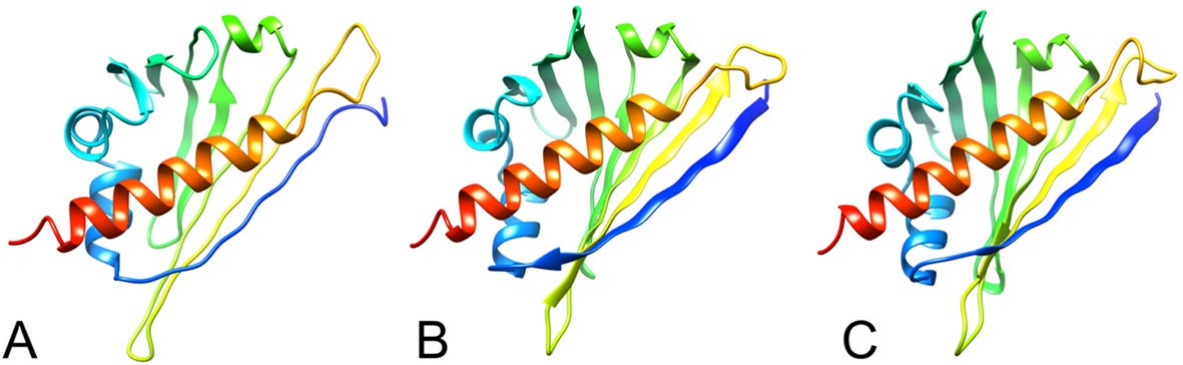
Final 3D structural models of uncharacterized protein LOC110648447 isoform X1 (**A**), isoform X2 (**B**), and isoform X3 (**C**) using I-TASSER. The C-scores of models are 0.14, 0.43, 0.31, while the estimated TM-scores are 0.73 ± 0.11, 0.77 ± 0.10, 0.75 ± 0.10, and the estimated RMSD are 4.5 ± 3.0A^o^, 3.9 ± 2.7A^o^, 4.1 ± 2.8A^o^ for isoforms X1, X2, and X3, respectively. The N-terminal to C-terminal sites of each protein isoform are color-shaded blue to red, visualized by UCSF Chimera software

The secondary structures of the 3 isoforms of the uncharacterized protein LOC110648447 were analyzed by I-TASSER, as shown in Supplementary Figs. 8 and 9. The results support those predicted by PSIPRED. According to I-TASSER, the confidence score (C-score) is estimated for the quality of predicted models and calculated based on the significance of threading template alignments and the convergence parameters of the structural assembly simulations. C-score values typically fall in the range of -5 to 2. The C-scores of the final protein structural models for isoforms X1, X2, and X3 were 0.14, 0.43, and 0.31, respectively, which are well within the standard range (Fig. 6, Supplementary Table 5). The models of these 3 isoforms showed characteristics of Bet v 1, consisting of three α helix motifs and seven antiparallel β strands arranged to form a large internal hydrophobic cavity involved in ligand binding [19].

To further predict the possible function and properties of the uncharacterized protein LOC110648447, the structural similarity between our models (Fig. 6) and templates from the protein data bank (PDB) was evaluated by the template modeling score (TM-score), and the sequence identity in the structurally aligned regions was determined. With good annotation scores (TM-score = 0.797–0.896) and around 94 percent sequence coverage, the top ten models suggest that the uncharacterized protein LOC110648447 is closely related to proteins in the PR-10 or Bet v 1 superfamily (Supplementary Tables 3, 4, 6).

Modeling the uncharacterized protein LOC110648447 by the I-TASSER server also predicts gene ontology (GO) terms based on C-scores for the query proteins. Each modeled protein is usually associated with multiple GO terms, including those related to biological processes, molecular functions, and cellular components (Table 5; Supplementary Tables 7.3, 7.4). For example, in terms of biological processes, a defense response (GO:0006952) and a response to biotic stimuli (GO:0009607) are predicted for all 3 isoforms with a GO score of 0.88–0.94.

**Table 5:** Consensus GO terms amongst the 10 top scoring templates. The GO-Score associated with each prediction is defined as the average weight of the GO term, where the weights are assigned based on the Cscore^GO^ of the template (Supplementary 4)

	Molecular function		Biological process		Cellular component	
	GO	GO score	GO	GO score	GO	GO score
Isoform X1	GO:0010427 ABA binding	0.39	GO:0006952 Defense response	0.88	GO:0005737 Cytoplasm	0.39
	GO:0004872 Signaling receptor activity	0.39	GO:0009607 Response to biotic stimulus	0.88	GO:0005634 Nucleus	0.39
	GO:0042803 Protein homodimerization activity	0.39	GO:0009738 ABA-activated signaling pathway	0.39		
Isoform X2	GO:0016787 Hydrolase activity	0.42	GO:0006952 Defense response	0.94	None was predicted	
			GO:0009607 Response to biotic stimulus	0.94		
Isoform X3	GO:0004872 Signaling receptor activity	0.39	GO:0006952 Defense response	0.88	GO:0005737 Cytoplasm	0.64
	GO:0010427 ABA binding	0.39	GO:0009607 Response to biotic stimulus	0.88	GO:0005634 Nucleus	0.39
	GO:0042803 Protein homodimerization activity	0.39	GO:0009738 ABA-activated signaling pathway	0.39		

**Fig. 7:**
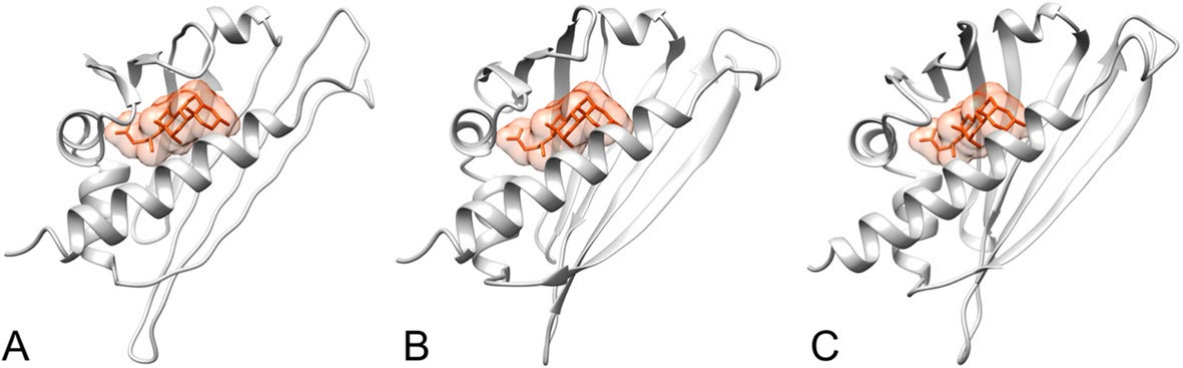
The ligand binding site predictions of the uncharacterized protein LOC110648447 by I-TASSER. Deoxycholate (orange) is predicted to bind in the cavity of uncharacterized protein LOC110648447 isoforms X1 (**A**), X2 (**B**), and X3 (**C**), respectively, with C-scores of 0.15, 0.18, and 0.19 and cluster sizes of 30, 34, and 32 (Supplementary Table 7.1). The structure of the predicted protein and ligand binding cavity was visualized using UCSF Chimera software

For the prediction of protein–ligand binding sites, the structure-based biological function annotation of the uncharacterized protein LOC110648447 was performed using the BioLiP database (Supplementary Table 7.1). The predicted docking models of isoforms X1, X2, and X3 showed that the proteins bind with a deoxycholic acid ligand with C-scores of 0.15, 0.18, and 0.19, respectively (Fig. 7). Abscisic acid was also predicted as a ligand of isoform X1 with a C-score of 0.15.

**Fig. 8:**
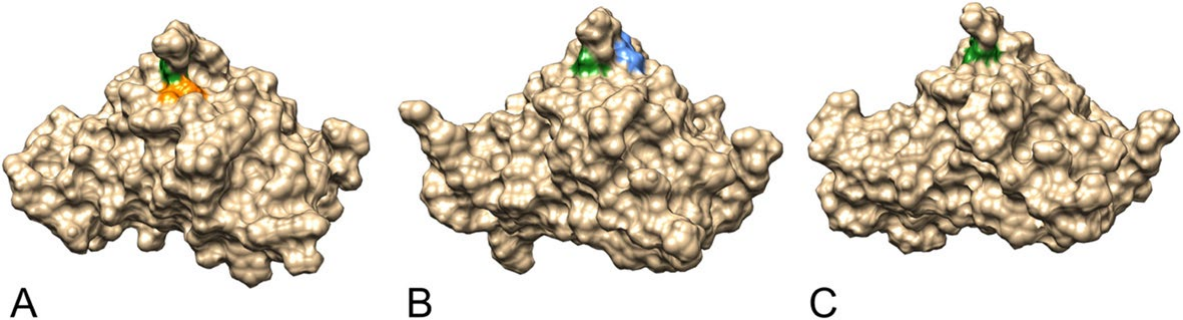
Predicted enzymatic active sites on the predicted 3D structures of uncharacterized protein LOC110648447 isoform X1 (**A**), isoform X2 (**B**), and isoform X3 (**C**) by I-TASSER and visualized by UCSF Chimera software. Norcoclaurine synthase activity (EC:4.2.1.78) was predicted to be performed by isoform X1 at residues Gly49 (green) and Leu68 (orange) with a C-score^EC^ = 0.386, by isoform X2 at residues Ile45 (blue) and Gly49 (green) with a C-score^EC^ = 0.349, and by isoform X3 at residue Gly49 (green) with a C-score.^EC^ = 0.415

Additionally, the catalytic sites for the enzymatic activity of the uncharacterized protein were predicted by I-TASSER (Fig. 8; Supplementary Table 7.2). The results revealed Gly49 and Leu68 residues as putative active sites that perform norcoclaurine synthase activity (Enzyme Commission (EC) number EC:4.2.1.78) in isoform X1 (C-score^EC^ 0.386), while only Gly49 alone was predicted to perform this activity in isoform X3 (C-score^EC^ 0.415). The norcoclaurine synthase activity in isoform X2 was predicted to be carried out by Ile45 and Gly49 active sites (C-score^EC^ 0.349).

## Discussion

Plant pathogens destroy many important economic plants worldwide. Although the interactions between plant hosts and pathogens have been investigated using classical biochemical and molecular biological approaches [28, 68], proteomics is an effective methodology that provides global information on various cellular protein networks [56]. To our knowledge, there have been few comparative proteomic studies of the rubber tree leaf response induced by the root rot fungi, *R. microporus*. In this study, leaf proteome changes in rubber tree clone RRIM600 during interactions with *R. microporus* and subsequent changes in transcript abundance of selected modulated proteins were assessed. The rubber tree clone RRIM600 is considered a susceptible clone to white root rot disease [97]. Here, the possible response mechanisms that occur in rubber tree leaves during pathogen infection are discussed.

### White root rot fungi negatively affect the rubber tree defensive proteins found in leaves

Many defense-related proteins, including a 17.3-kDa class I heat shock protein-like protein, a superoxide dismutase [Cu–Zn]-like protein, an ATP synthase subunit D mitochondrial-like protein, and a universal stress PHOS34 protein, were down-regulated both at the protein and transcription levels after inoculation with *R. microporus* (Table 1 and Fig. 3).

The major heat shock proteins (HSPs) function as molecular chaperones and protect cells against the deleterious effects of stress, including stress caused by pathogen infection [31, 104]. HSPs are conservatively recognized according to their molecular mass and classified into five major families: HSP100, HSP90, HSP70, HSP60, and 17-to-30-kDa small HSPs (sHSPs) [96]. In this study, a 17.3-kDa class I heat shock protein-like protein was found to be down-regulated after rubber tree infection by *R. microporus*. The transcription levels of the 17.3-kDa HSP decreased during 10–50 DAI (Fig. 3). Similarly, a 17.3-kDa HSP was found to decrease steadily during the 5 h after infection (hai) in an avocado root infected with *Phytophthora cinnamomic*, a soil-borne oomycete [2]. In rice, small HSPs showed differential responses to infection by *Magnaporthe grisea*, which were growth stage-specific,four of the sHSPs (HSP16, 17, 18.1 and 18.2) were up-regulated, while another four (HSP16.6, 17.8, 18.8 and 22) were down-regulated [79]. sHSPs are speculated to be involved in the adjustment to invading pathogens, probably by maintaining newly synthesized stress-related proteins that are correctly folded by the HSP70 or HSP60 chaperone complexes [16].

Universal stress protein (USP) genes encode proteins containing the 140–160 highly conserved residues of the USP domain with other catalytic motifs and play a crucial role in diverse aspects of plant growth and development, including abiotic and biotic stress resistance [10]. In *Arabidopsis*, two USPs, including AtPHOS32 and AtPHOS34, are phosphorylated by mitogen-activated protein kinases (MAPKs) after treatment with *Phytophthora infestans* zoospores or the bacterial-eliciting peptide, flagellin-22 [45, 55]. Phosphorylated USPs seem to be involved in the activation of pathogen defense and then provide protection against pathogenic attacks. Recently, functional characterization of an *Arabidopsis* USP showed that it plays a critical role in the plant's tolerance to diverse pathogenic infections with novel antifungal activity [66]. This protein has been found to efficiently suppress fungal growth in fungal cells by inducing ROS. In this study, the universal stress protein PHOS34 in rubber trees was found to be down-regulated in leaves after being infected by *R. microporus*. This might be because leaves are not the site of this pathogenic infection. It would be interesting to further analyze the expression of this protein in the root of the rubber tree.

The balance between ROS production and scavenging performed by antioxidant molecules is one of the major defense responses of the host plant to pathogens. Well-balanced ROS levels in the host plant could serve as a defense-triggered signaling molecule, while an overload of accumulated ROS can cause toxicity to plant cells [25, 38]. The homolog of the superoxide dismutase [Cu–Zn] (CuZn-SOD) enzyme, which is responsible for the dismutation of highly active O_2_^−^ to H_2_O_2_ before being scavenged by the catalase and/or peroxidase enzymes, was identified here as a down-regulated protein due to *R. microporus*. A previous study showed that a rubber tree CuZn-SOD protein was up-regulated in the leaves of the clone PB314 challenged by the ascomycete *Pseudocercospora ulei* [41]. In addition, the CuZn-SOD overexpressed transgenic plants were proven to have better scavenging of salinized-induced ROS compared to wild-type plants [34]. Accordingly, the down-regulation of a CuZn-SOD-like protein and transcript in this study suggests impaired ROS scavenging in the leaves of rubber tree clone RRIM 600 after *R. microporus* infection.

Remarkably, there are increasing reports indicating that, besides its direct role in converting light energy to chemical energy, photosynthesis can also regulate plant response to both biotic and abiotic stresses [37]. Oxygen-evolving enhancer proteins (OEEs) are important proteins for oxygen evolution and Photosystem II (PSII) stability. OEEs consisting of three subunits, OEE1 (33 kDa, PsbO), OEE2 (23 kDa, PsbP) and OEE3 (16 kDa, PsbQ), are nuclear-encoded chloroplast proteins which bind to the periphery of PSII [8, 74]. Enhanced expression of OEEs under stress has been reported. After being exposed to the powdery mildew pathogen, *Sphaerotheca fuliginea* (Sf), the abundance of OEE1 and OEE2 proteins was higher in the leaves of resistant cucumbers than in the leaves of susceptible ones [15]. This finding suggests that OEE1 and OEE2 play an important role in the maintenance of PSII activity under pathogen infection. In addition, the higher expressions of OEE1 and OEE2 are some of the protective responses exhibited by resistant cucumbers to powdery mildew disease. In other instances, oxygen evolving proteins seem to be the strategic target of plant pathogens. Recently, the 23-kDa VpOEE2 or VpPsbP in grapevine was identified as a host target of a *Plasmopara viticola* RXLR effector, resulting in reduced H_2_O_2_ accumulation and activating the ^1^O^2^ signaling pathway through stabilizing PsbP, thereby promoting disease in grapevines [50]. The bacterial pathogen *Pseudomonas syringae* secretes an HopN1 effector or cysteine protease that targets an OEE3 or PsbQ protein in tomato plants, which interferes with ROS production in chloroplasts and reduces defense response in leaves [73]. In this study, an OEE3-2 protein was identified from the 17-kDa band obtained from 1D-PAGE. According to RT-qPCR analysis, the OEE3-2 gene was down-regulated after *R. microporus* inoculation. The reduced transcription of OEE3-2 suggests another negative response of photosynthetic machinery to pathogen infection in rubber trees.

Matrix metalloproteinases (MMPs), also known as matrixins, are a group of endoproteinases containing a zinc ion in their catalytic center. The expression patterns of MMPs in plants suggest that they play an important role in remodeling plant tissue during growth and development, and in response to biotic and abiotic stresses [11, 51, 80, 71]. *Nicotiana benthamiana metalloprotease 1* encodes NtMMP1 in tobacco plants. Its expression increased after infection by pathogens and after treatment with hormones. Plants overexpressing NtMMP1 exhibited greater resistance to infection, while the silenced plants exhibited greater susceptibility to infection by *Phytophthora infestans* compared to control plants [26]. The role of some *Arabidopsis* MMPs in fungal resistance was also proved in genetically repressed and overexpressed transgenic plants [109]. In this study, the metalloendoproteinase 5-MMP-like protein in the susceptible rubber tree clone (RRIM 600) was down-regulated at the transcription level, while there was greater accumulation at the protein level after fungal infection. The role of this gene should be further analyzed in other resistant clones of rubber tree.

An ATP synthase subunit D protein is a mitochondrial ATP synthase or complex V according to the Uniprot protein accession Q9FT52 of *Arabidopsis*. Many plant species show a decreased abundance of ATP synthase subunits during cold stress [12, 39, 69, 88, 100]. Here, the subunit D of a mitochondrial ATP synthase in rubber tree leaves was reduced in response to *R. microporus*. Our result was related to the downregulation of mitochondrial ATP synthase in tobacco responding to aggressive cucumber mosaic virus (CMV-R3E79R) [21] and *Pseudomonas syringae* pv. *tabaci* [87]. Gellért and colleagues [21] found that the CMV-R3E79R proteins interact with the tobacco ATP synthase F1 motor complex and lethally block its rotation, which may lead to cell apoptosis. These findings suggest a negative interaction between the pathogen and plant ATP synthase.

### The novel uncharacterized protein LOC110648447 might mediate rubber tree response to white root rot fungi

Most of the up-regulated protein spots at 10, 30, and 50 DAI, as well as the major protein from the 17-kDa band from 1D-PAGE, were identified as the uncharacterized protein LOC110648447 (isoforms X1, X2, X3) (Figs. 1, 2; Table 1). The significantly higher expression of the uncharacterized protein LOC110648447 isoform X1 after fungal infection was confirmed by RT-qPCR (Fig. 3). *Cis*-acting regulatory elements previously reported to play a crucial role in regulating defensive genes as well as in drought response were found on the uncharacterized protein LOC110648447 (isoform X1) gene of the rubber tree clone RRIM600 (Table 3). These results suggest that the uncharacterized protein LOC110648447 likely plays a role in rubber tree response to *R. microporus* invasion.

Three GT1GMSCAM4 and 3 BIHDOS elements were found on the uncharacterized protein LOC110648447 promoter of the RRIM600 rubber tree clone. GT1GMSCAM4 element (GAAAAA motif) is known to play a role in pathogen response [65] and also in fungal elicitor response [93]. BIHDOS elements are homeodomain transcription factor OsBIHD1 binding sites [52], which were previously reported in the promoter region of pathogen-responsive and pathogen-inducible (*PRPI*) defensin genes [42] and involved in disease response [93]. W-box, a wounding-responsive element, and several pathogen-responsive elements (including WBOXNTERF3, WRKY71OS, and WBOXATNPR1) were also found. The WBOXNTERF3 has been reported to be related to wounding response in the tobacco ethylene-responsive transcription factor 3 (*ERF3*) gene [58]. The WRKY71OS element is a binding site of rice WRKY71 [99], and this element was also present in the parsley *PR-10* gene promoter, which proved to interact with parsley WRKY proteins [14]. The WBOXATNPR1 was found on an *Arabidopsis* disease resistance regulatory *NPR1* (Nonexpresser of PR genes 1) gene and recognized by salicylic acid (SA)-induced WRKY proteins [9, 105]. Interestingly, many sites of w-box founded on the promoter of the barley germin-like *GER4* gene were related to necrotrophic and biotrophic pathogen induced gene expression [27]. The mutation of the w-box element in rice *OsPR-10* showed an impaired stress hormone response [30]. Another SA-activated element found in this study is the ASF1MOTIFCAMV element (TGACG), which is an activating-sequence factor 1 (ASF-1) protein binding motif [13], [72]. The presence of these SA-responsive elements suggests a potential response of gene transcription induced by the stress hormone SA that might accumulate after *R. microporus* infection.

The drought-responsive MYB1AT, MYCATERD1, and PREATPRODH elements found in this study indicate that this uncharacterized protein gene might play a role in plant response to environmental stress. The MYB1AT element identified in the dehydration-responsive gene was recognized by MYB proteins [1], well-known for their importance in stress response. The pro- or hypoosmolarity-responsive element (PRE) named PREATPRODH in the proline dehydrogenase gene is induced by low osmolarity conditions [75, 76]. The MYC recognition site MYCATERD1 binds specifically to NAC proteins [83, 92], which are proteins related to ABA signaling [60] and plant immunity [106].

**Fig. 9:**
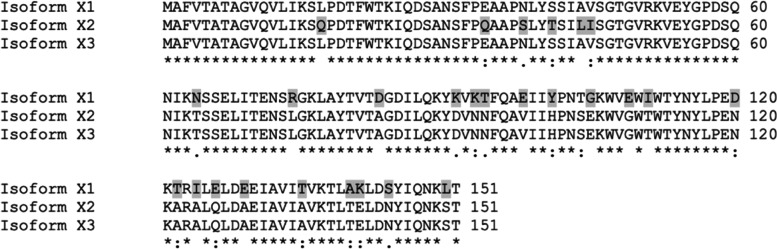
The alignment of amino acid sequences of three isoforms of the uncharacterized protein LOC110648447 by Clustal W multiple sequence alignment

From the genome sequence of rubber tree clone Reyan 7–33-97, there are three closely related isoforms of the uncharacterized protein LOC110648447. These 3 isoforms are likely derived from alternative splicing within the same genomic locus, as shown in the NW_018447361.1 reference sequence. The arrangement of exon–intron encoding in each isoform (Fig. 4) and the amino acid alignment (Fig. 9) show that isoforms X1 and X3 have identical amino acid sequences for the first 57 residues, and that isoforms X2 and X3 have identical sequences for the next 94 residues. Isoform-specific fragments might be selected by using the fragment encoded by the second exon of isoform X1, i.e., NSSELITENSR, or by using the fragments encoded by the first exon of isoform X2, i.e. IQDSANSFPQAAPSLYTSILISGTGVR (Supplementary Table 1). There is no amino acid fragment specific to isoform X3. Different from Reyan 7–33-97, the identical sequences of the isoforms X1 and X2 were present in different contigs of the RRIM 600 whole genome shotgun sequence (contig MKXE01009552.1 for isoform X1, and contigs AJJZ011011755.1 and MKXE01140403.1 for isoform X2).

### The uncharacterized protein LOC110648447 is classified as a member of Bet v 1 protein family

Structural and functional annotations of particular uncharacterized proteins may result in the identification of new structures, aiding in the introduction of new protein functions and interactions. This study aimed to create the first 3D model of the uncharacterized protein LOC110648447, which significantly increased after *R. micoporus* infection. As a result, this protein was classified as a pathogenesis-related 10 (PR-10) protein, also known as the Bet v 1 protein family (Table 4). This protein family contains numerous members from plants and also bacteria, which exhibit Bet v 1-like folding characteristics. The “hotdog fold/domain” typically consists of seven antiparallel β-sheets, two V-shaped α-helices, and a long C-terminal α-helix, forming a large hydrophobic cavity for ligand binding [70]. In this study, the topology of the secondary structure arrangement resulting from PSIPRED prediction (Fig. 5) showed a “β-α1-β6 and C-terminal α-helix” structure, strongly supporting the classification of uncharacterized protein LOC110648447 into this family.

The Bet v 1 protein family is divided into several groups, including intracellular pathogenesis-related (IPR) proteins, which are classic PR-10 proteins; cytokinin-specific binding (CSBP) proteins; (S)-norcoclaurine synthases (NCS) proteins; and major latex (MLP) proteins [3, 19]. The MLP proteins are known to perform functions in many plant species, such as an MLP-like 43 protein of *Arabidopsis*, which mediates drought stress via ABA signaling [95], and an MLP28 protein of cotton, which plays a role in defense against *Verticillium dahlia* [101]. In the genus *Fragaria*, strawberry-related plants showed an increased expression of *PR-10* genes in response to fungal pathogens [24, 32, 91, 102]. Cowpea and barley PR-10 s were linked to cultivars resistant against *Rhynchosporium secalis* and *Uromyces vignae* infection. [57, 86]. Two antifungal MdPR10 proteins of apple were recently confirmed to confer resistance to Alternaria leaf spot disease due to their interaction with leucine-rich repeat proteins [108]. A PR-10 protein was suggested as a biomarker of Fusarium head blight (FHB) resistance due to its high abundance in a comparative iTRAQ/MS proteomic study of *Fusarium graminearum*-resistant and susceptible wheat lines [94]. Interestingly, the root rot pathogen *Fusarium solani* triggered the expression of a defense-related PnPR10 protein of *Panax notoginseng*, which showed RNase activity in vitro [47].

In rubber trees, the potential PR10 proteins identified here not only showed sequences and secondary structures related to PR10/Bet v 1, but were also in the same molecular weight range of this protein family (approximately 17 kDa) (Figs. 1, 2).

The promoter of this gene in rubber trees was also predicted to respond to various biotic stressors, such as necrotrophic and biotrophic pathogens (Table 3), which are proposed to be involved in the up-regulation of gene transcripts during *R. microporus* inoculation. In addition, the predicted GO terms related to biological processes suggest with a relatively high score of prediction that they may play a role in defense response against biotic stress (Table 5), similar to other members in the PR10/Bet v 1 protein family. Although we only hypothesized that the PR10s here participate in fungal defense in rubber tree leaves in some aspects, prediction of the GO terms’ molecular functions shows a moderate score of hydrolase, protein homodimerization, ABA-binding, and signaling receptor activities.

### Structure prediction by I-TASSER suggests possible phytohormone binding sites and active sites of novel rubber tree PR-10 s

The predicted structures of uncharacterized protein LOC110648447 clearly showed the conserved topology of Bet v 1 folding with relatively low C-scores, except for isoform X1 (Fig. 6). The C-scores of protein prediction by I-TASSER ranged between -5 and 2, indicating acceptable quality and confidence. Accordingly, the low C-scores of our predicted models may be due to a lack of closely related protein templates. However, these predicted structures were annotated to the PDB database and received high TM-scores when aligned with the Bet v 1/PR-10 related templates in the PDB (Supplementary Table 6). This result provides additional strong evidence for classifying the uncharacterized protein LOC110648447 into the Bet v 1/PR-10 family.

The various proteins of the Bet v 1-like structure specifically bind with many groups of phytohormones and metabolites [4]. The hydrophobic binding pocket of Bet v 1 may also perform a storage role for certain phytohormones before releasing them under specific conditions [3]. For example, phytoprostane PPE1 binding to a Bet v 1 protein has been shown to protect the protein from cysteine protease [85].

In silico prediction of protein binding sites and ligands revealed a possible hydrophobic cavity of the novel rubber tree PR-10 proteins, which might be the site for abscisic acid binding in isoform X1, or deoxycholate binding in all three isoforms (Fig. 7). In this study, the mutated PYR1 protein (PDB: 3ZVU) was used as the template structure for the ABA binding site prediction in I-TASSER (Supplementary Table 7.1). The pyrabactin-resistant (PYR) protein is an ABA receptor that is homologous to the Bet v 1 protein family [70], and hence, binding with ABA may be possible for Bet v 1 structural-related proteins. Deoxycholate is a bile acid molecule that can exogenously induce *Arabidopsis* defenses against bacterial pathogens [107]. The crystal structure of the major birch allergen Bet v 1 a protein (PDB: 4A83) was shown to be able to bind to deoxycholate. This protein was also selected as the template for ligand binding site prediction for all three isoforms of the rubber tree PR10 protein. Although the C-scores of each binding site prediction exhibit low confidence (< 20% confidence), the results still revealed that the large cavity formed in the protein models possibly serves as a binding pocket for phytohormones and other small molecules (Fig. 7). The molecules that naturally interact with this novel PR-10 of the rubber tree still require further investigation.

Many studies of PR-10 proteins have been reported for their RNase activities [40, 98, 110, 43] and cysteine protease inhibitor activity [5]. However, these two enzyme activities were not predicted by our PR-10 protein in the rubber tree. According to enzyme classification by I-TASSER, the specific residues of novel rubber tree PR-10 were predicted to have norcoclaurine synthase (NCS) activity (EC:4.2.1.78) (Fig. 8, Supplementary Table 7.2). The reaction by the NCS enzyme generates a six-membered ring by forming a bond between C-6 of the 3,4-dihydroxylphenyl group of dopamine and C-1 of the aldehyde in the imine formed between the substrates. The product is the precursor of benzylisoquinoline alkaloids in plants. According to the MetaCyc database (ID:PWY-3581), NCS enzymes participate in the biosynthesis of (S)-reticuline, which is an intermediate in the biosynthesis of many antimicrobial molecules such as coptisine and berberine. The proteins showing NCS activity were found in *Thalictrum flavum* [77] and *Papaver somniferum* [49]. The designated plant PR-10 from *Coptis japonica* (Uniprot: A2A1A1) and *Papaver somniferum* (Uniprot: Q4QTI9 and Q4QTJO) showed very similar characters to NCS proteins. We hypothesize that the predicted active site may be significant for the activity of PR-10 during secondary metabolite biosynthesis. It would be interesting to investigate whether the novel PR-10 protein can perform NCS activity. Defensive alkaloids in rubber tree leaves may be synthesized due to the higher expression of this protein when challenged by the white root rot fungi.

**Fig. 10:**
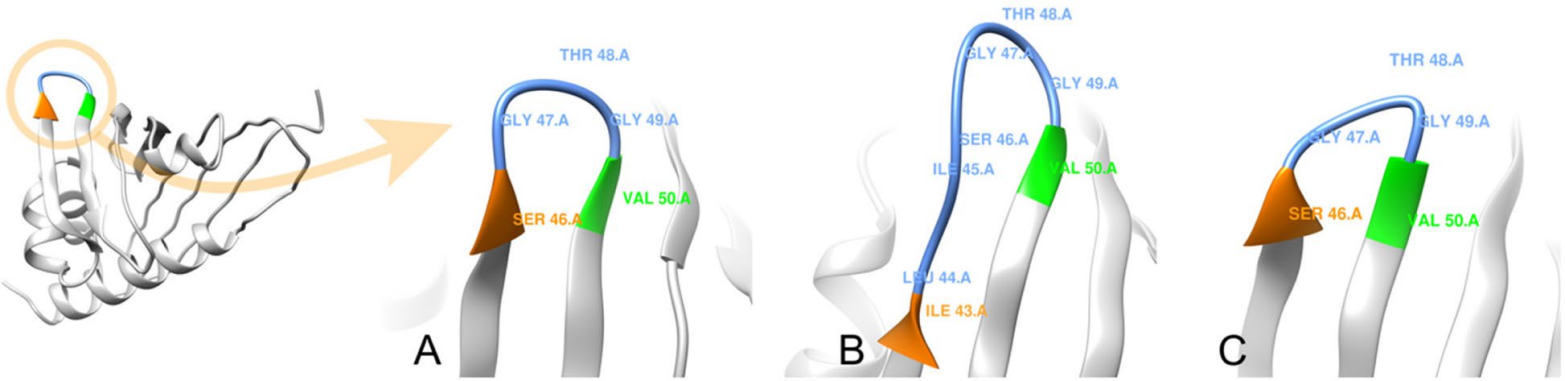
The P-loop (blue) connects strands 2 (orange) and strands 3 (green) in the predicted structure of the novel rubber tree PR-10 (uncharacterized protein LOC110648447) isoform X1 (**A**), isoform X2 (**B**), and isoform X3 (**C**)

The most conserved region of PR-10 proteins is located at the connecting loop between beta strands 2 and 3, called the P-loop or Glycine-rich loop [78]. Many of them exhibit nucleotide-binding ability. We noticed that the structure of the Glycine-rich loop for all 3 rubber tree PR-10 isoforms (Fig. 10) was similar to the superposition of various PR-10 structures reported by Fernandes and colleagues (2013). The P-loop of two soybean PR-10 proteins, GmPR10 and Gly m 41, were recently proved to exhibit anti-pathogen function [17]. A couple of glycine residues (GXG) in the P-loop were also found in a potato PR-10 at positions 45–47 [54]. A PR-10 protein lacking the P-loop was observed in MLP28, another group of PR-10 proteins in *Arabidopsis* [53]. The novel rubber tree PR-10 in this study showed a couple of conserved glycine residues (Gly47 and Gly49) in the P-loop, of which Gly49 is the predicted active site in all three isoforms (Figs. 8, 10). The P-loop observed in the uncharacterized protein LOC110648447 sequence strongly supports the likelihood that this protein is a novel PR-10 of the rubber tree.

## Conclusions

This research demonstrates that the response of rubber trees to white root rot disease is systemically linked between the infected root and other distal parts such as the leaves. Many defense-related genes and proteins were down-regulated, such as the molecular chaperone, 17.3-kDa HSP, and an ROS-scavenging enzyme, Cu–Zn SOD. Following infection by *R. microporus*, the novel PR-10 increased substantially at both the transcript and protein levels. The Bet v 1-related structure of the novel PR-10, as well as its ligand-binding sites and putative enzymatic activity, were predicted and hypothesized for their defense function. However, further validating the defense ability of this protein would be beneficial for understanding the distal response of rubber trees against fungi. The information from this study will be useful for white root rot disease management of rubber trees in the future.

## Methods

### Pathogen inoculation and sample collection

*Rigidoporus microporus* NK6, a virulent strain, was isolated from the root of a diseased rubber tree in southern Thailand. Pure fungal mycelia were cultured on potato dextrose agar (PDA). The mycelia on PDA were then cut into 0.5 cm diameter discs and used to inoculate 100 g of sterile sorghum seeds in Erlenmeyer flasks. Each flask was inoculated with 5 PDA discs and incubated at 30 °C for 14 days. RRIM 600 grafted rubber tree seedlings (8 months old) were used in this experiment. Experimental seedlings were inoculated with fungal culture placed next to the roots at the bottom of the tree pot. Control plants were mock inoculated with fungus-free cultured material. The experiment was conducted using three replicates under greenhouse conditions. The infection of rubber trees was validated by observed the tree morphology and symptoms. At 10, 30, 50 DAI, the trees did not show the symptom aboveground, but the roots were completely infected since 10 DAI as shown in Supplementary Fig. 1. Leaf samples were collected at 10, 30, and 50 DAI and immediately frozen in liquid nitrogen before -80 °C storage for subsequent protein and RNA extraction.

### Protein extraction, decontamination, and quantification

Leaf proteins were extracted from 3 g of ground leaf sample using a phenol-based protocol modified from Hurkman and Tanaka [29]. The sample was mixed with 5 mL of extraction medium (0.1 M Tris–HCl pH 8.8, 10 mM EDTA, 0.4% 2-Mercaptoethanol, 0.9 M sucrose) and 5 mL of phenol buffer saturated with Tris–HCl pH 8.8. The samples were vortexed for 30 s. The mixture was divided and transferred into 1.5-mL microcentrifuge tubes and then centrifuged at 10,000 × g for 30 min at 4 ^o^C to separate the mixture. The phenol phase was collected and transferred new microcentrifuge tubes (200 µL per tube). One ml of -80 °C pre-chilled precipitation buffer (0.1 M ammonium acetate in absolute methanol) was added into each tube and incubated at -80 °C for at least 2 h to precipitate proteins in the phenol phase. The mixture was then centrifuged at 10,000 × g for 30 min at 4 ^o^C again to obtain the protein pellet, and the supernatant was discarded. Afterwards, the pellet was washed twice with Washing buffer I (cold 0.1 M ammonium acetate in absolute methanol containing 10 mM DTT) and once with Washing buffer II (cold 80% acetone containing 10 mM DTT) by vortexing the pellet in 1 mL of washing buffer and centrifuging at 10,000 × g for 30 min at 4 oC. The pellet was then air-dried for 3–5 min and rehydrated in an optimal volume of Rehydration buffer (7 M Urea, 2 M Thiourea, 30 mM DTT, 4% CHAPS). The rehydrated protein was kept at -80 °C until analysis. Then, the concentration of protein was triplicate measured at an absorbance of 595 nm using Bradford’s assay reagent system (Bio-Rad, USA). The pooled samples were decontaminated using a 2D Clean-Up kit (GE Healthcare, USA) following the manufacturer’s protocol.

### Comparative proteomic analysis using 1D- and 2D-PAGE

The protein samples were separated by 1 dimensional- and 2 dimensional- polyacrylamide gel electrophoresis (1D- and 2D-PAGE). For 1D-PAGE, the protein samples (10 μg) were separated by 12.5% polyacrylamide gels as described by Laemmli [44]. For 2D-PAGE, equal amounts of protein (60 μg) from each sample were separated as follows. In the first dimension, IPG strips (GE Healthcare, USA) that were 7 cm in length and pH 3–10 were used. Electrophoresis was performed following the specific conditions described by the manufacturer. After isoelectric focusing (IEF), the proteins were separated by SDS-PAGE in the second dimension using 12.5% polyacrylamide gels. The gels were stained using the Coomassie Blue-G staining method. For each biological replicate, one set of high-resolution gels, run at different times, was selected for further analysis. The relative abundance of each protein spot was quantified. The gel images were scanned by a UMAX image scanner coupled with Labscan software (GE Healthcare, USA). For the 2D-PAGE profile, protein spots were detected, matched, and volume-quantified using ImageMaster 2D platinum v.6.0 software (GE Healthcare, USA).

### Protein identification

The fold-change of each candidate protein spot was calculated from the ratio of their relative volumes. The selected protein band and spots were excised from preparative gels and sent for protein identification by ESI-QUAD-TOF mass spectrometry at Salaya Central Instrument Facility, Mahidol University, Thailand. The resultant data were searched against databases using MASCOT (www.matrixscience.com) to identify the annotated names of the sequenced proteins.

### RNA extraction, decontamination, and quantification

The RNA sample of each treatment was isolated from 0.2 g of ground leaf sample using Pure Link™ Plant RNA reagent (Invitrogen, USA) according to the manufacturer’s protocol. The removal of genomic DNA contamination in isolated RNA samples was performed using the DNA-free™ DNA removal kit (Invitrogen, USA) and DNase-treated samples were quantified by NanoDrop spectrophotometer. The cDNA synthesis was then carried out using the SuperScript™ III first-strand synthesis system (Invitrogen, USA). The resulting concentrated cDNA was kept at -20 °C for expression analysis.

### Primer design and efficiency validation

The primers of each selected gene were designed from available sequences in GenBank accessions (Table 2). The primer sequences and amplicon regions which were potentially suitable for Reverse Transcription Quantitative PCR (RT-qPCR) amplification were investigated using NCBI Primer BLAST (https://www.ncbi.nlm.nih.gov/tools/primer-blast/) and further observed for self-dimers and heterodimers by the IDT oligo analyzer (https://www.idtdna.com/calc/analyzer) to obtain the suitable primer pairs for RT-qPCR. The annealing temperature (T_a_) of each primer for RT-qPCR was optimized. The primer efficiency (E) was validated using serial-diluted pooled cDNA of leaf samples for RT-qPCR. The slope obtained from plotting C_T_ values (y-axis) against the log of concentration (x-axis) was used for efficiency calculation, following the formula E = (10^-1/slope^ – 1) x 100.

### Relative gene expression analysis by RT-qPCR

The total 20-μL mixture used in RT-qPCR reaction contained 13.6 μL of H_2_O, 2 μL of 10X Buffer, 0.8 μL of 50 mM MgCl_2_, 0.4 μL of 10 mM dNTP mix (Biotechrabbit, Germany), 0.4 μL of 20 mM forward primer, 0.4 μL of 20 mM reverse primer, 0.24 μL of SYBR Green (Sigma, USA), and 2 μL of 1:25 diluted sample cDNA. Thermal cycling was performed by the ABI 7500 fast real-time PCR system (Applied Biosystems, USA), set as follows: initial denaturation at 94 °C for 3 min, followed by 40 cycles of denaturation at 95 °C for 15 s and annealing at the optimized temperature of each primer pair, followed by polymerization at 72 °C for 1 min. The C_T_ values of the target gene were collected for the calculation of relative gene expression, normalized with the *UBC2a* reference gene [46]. The C_T_ data obtained from 3 biological and 9 technical replicates (*N* = 27) were analyzed for the fold-change in relative expression, following the method described by Taylor et al. [90]. Statistical analysis was carried out using analysis of variance (ANOVA) and Tukey’s post hoc significance test at *p* < 0.05 in SPSS software (IBM, USA).

### Cis-acting element analysis of uncharacterized protein LOC110648447

A genomic sequence of the uncharacterized protein LOC110648447 was BLAST searched against whole-genome shotgun contigs of rubber tree (id:3981) in the NCBI database. The hit contig MKXE01009552.1 from clone RRIM600 was selected for the analysis. A sequence of 3000 bp at the 5’-end upstream before the coding sequence was chosen to search for a *cis*-acting element via the NEWPLACE database (https://www.dna.affrc.go.jp/PLACE/?action=newplace) and for functional analysis of the obtained elements.

### Structure-based in silico predictions of uncharacterized protein LOC110648447

The amino acid sequences of uncharacterized protein LOC110648447 isoforms were submitted to online servers for the secondary structure and 3D structure predictions using the PSIPRED 4.0 protein secondary structure prediction server (http://bioinf.cs.ucl.ac.uk/psipred/) and Iterative Threading ASSEmbly Refinement (I-TASSER) (https://zhanglab.dcmb.med.umich.edu/I-TASSER/), respectively. I-TASSER predicts the functions of modeled proteins based on global and local similarity to template proteins in the Protein Data Bank (PDB) with known structures and functions. Here, the I-TASSER server was then applied to build the structural model of the uncharacterized protein using identified templates in the PDB database using 10 threading algorithms. The models were then refined and scored before further analyzing the function of the highest-scoring model based on structural matching with proteins in the BioLiP database [103]. The resulting output of template proteins, predicted structural models, proteins related to the predicted structural models, GO terms, ligand binding sites, and enzymatic active sites (summarized from the I-TASSER server) were obtained to determine the potential function of the uncharacterized protein.

## Supplementary Information

**Additional file 1: Supplementary Figure 1.** The phenotypes of mock- and *R. microporus*-inoculated rubber tree.** Supplementary Figure 2.** Raw Image of One-dimensional (1D) SDS-PAGE gel showed the comparison of the protein patterns of rubber tree leaves after *R. microporus *inoculation by SDS-PAGE with Coomassie brilliant blue (CBB) dye. Lane 1: protein marker; Lanes 2, 4, 6: mock inoculation at 10, 30, and 50 days after inoculation (DAI), respectively; Lanes 3, 5, 7: *R. microporus*-inoculation at 10, 30, and 50 DAI, respectively.** Supplementary Figure 3.** Raw Image of Two-dimensional (2D) SDS-PAGE gels. The three replicates of 2D SDS-PAGE of each treatment are showed below. The framed gels are the representative gels of each treatment.** Supplementary Figure 4.** Location of the coding sequence of the uncharacterized protein LOC110748447 isoforms on whole genome shotgun contig LVXX01001672.1 (Range: 61922-78548) *Hevea brasiliensis *cultivar reyan7-33-97 scaffold1672, whole genome shotgun sequence (matched 100% +/**-** ).** Supplementary Figure 5.** Location of the coding sequence of the uncharacterized protein LOC110748447 isoforms on whole genome shotgun contig MKXE01009552.1 (Range: 38570-39259) *Hevea brasiliensis *cultivar RRIM600, whole genome shotgun sequence (matched 100% +/- with isoform X1) The 3000 bp of 5’upstream region was the blue region.** Supplementary Figure 6.** Location of predicted cis-acting elements on the promoter region of Uncharacterized protein LOC110648447.** Supplementary Figure 7.** The secondary structure prediction of Uncharacterized protein LOC110648447 isoforms using PSIPRED server.** Supplementary Figure 8.** The secondary structure prediction of Uncharacterized protein LOC110648447 isoforms using I-TASSER server.** Supplementary Figure 9.** The predicted normalized B-factor of predicted secondary structure of Uncharacterized protein LOC110648447 isoforms using I-TASSER server.** Supplementary Table 1.** Identification results of the selected 1D-PAGE band and 2D-PAGE spots by ESI-QUAD-TOF mass spectrometry (MS/MS) analysis.** Supplementary Table 2.** Location of predicted cis-acting elements on the promoter region of Uncharacterized protein LOC110648447.** Supplementary Table 3.** The top 10 threading templates used by I-TASSER in predicting the 3D structures of Uncharacterized protein LOC110648447 isoforms.** Supplementary Table 4.** The alignment of Uncharacterized protein LOC110648447 isoforms with the top 10 threading templates in supplementary table 3.** Supplementary Table 5.** The top 5 final models of Uncharacterized protein LOC110648447 isoforms predicted by I-TASSER server.** Supplementary Table 6.** The top 10 structurally closed proteins in the PDB to the model of Uncharacterized protein LOC110648447 isoforms predicted by I-TASSER server.** Supplementary Table 7.** The predicted functions of Uncharacterized protein LOC110648447 isoforms by COFACTOR and COACH through I-TASSER server.

## Data Availability

The datasets generated in the present study are available from the corresponding author on reasonable request.

## Abbreviations

ABA: Abscisic Acid
ATP: Adenosine triphosphate
bp: Base pair
cm: Centrimeter
g: Gram
Gly: Glycine
H_2_O_2_: Hydrogen-peroxide
Ile: Isoleucine
In silico: Done/Produce using computer
iTRAQ/MS: Isobaric Tag for Relative and Absolute Quantitation and Mass Spectrometry
kDa: Kilo Dalton
LC–MS/MS: Liquid Chromatography with Tandem Mass Spectrometry
Leu: Leucine
M: Molar
min: Minutes
mL: Milliliter
mM: Millimolar
O_2_^−^: Superoxide
PAMP: Pathogen-Associated Molecular Pattern
pI: Isoelectric point
PSII: Photosystem II
µg: Microgram
µL: Microliter
