# Supplementary material for: A novel rubber tree PR-10 protein involved in host-defense response against the white root rot fungus Rigidoporus microporus
Source: BMC Plant Biol. 2023 Mar 22;23:157. doi: 10.1186/s12870-023-04149-3 (PMC10032002; doi:10.1186/s12870-023-04149-3)
Supplement: Supplementary file 1 — Additional file 1: Supplementary Figure 1. The phenotypes of mock- and R. microporus-inoculated rubber tree. Supplementary Figure 2. Raw Image of One-dimensional (1D) SDS-PAGE gel showed the comparison of the protein patterns of rubber tree leaves after R. microporus inoculation by SDS-PAGE with Coomassie brilliant blue (CBB) dye. Lane 1: protein marker; Lanes 2, 4, 6: mock inoculation at 10, 30, and 50 days after inoculation (DAI), respectively; Lanes 3, 5, 7: R. microporus-inoculation at 10, 30, and 50 DAI, respectively. Supplementary Figure 3. Raw Image of Two-dimensional (2D) SDS-PAGE gels. The three replicates of 2D SDS-PAGE of each treatment are showed below. The framed gels are the representative gels of each treatment. Supplementary Figure 4. Location of the coding sequence of the uncharacterized protein LOC110748447 isoforms on whole genome shotgun contig LVXX01001672.1 (Range: 61922-78548) Hevea brasiliensis cultivar reyan7-33-97 scaffold1672, whole genome shotgun sequence (matched 100% +/- ). Supplementary Figure 5. Location of the coding sequence of the uncharacterized protein LOC110748447 isoforms on whole genome shotgun contig MKXE01009552.1 (Range: 38570-39259) Hevea brasiliensis cultivar RRIM600, whole genome shotgun sequence (matched 100% +/- with isoform X1) The 3000 bp of 5’upstream region was the blue region. Supplementary Figure 6. Location of predicted cis-acting elements on the promoter region of Uncharacterized protein LOC110648447. Supplementary Figure 7. The secondary structure prediction of Uncharacterized protein LOC110648447 isoforms using PSIPRED server. Supplementary Figure 8. The secondary structure prediction of Uncharacterized protein LOC110648447 isoforms using I-TASSER server. Supplementary Figure 9. The predicted normalized B-factor of predicted secondary structure of Uncharacterized protein LOC110648447 isoforms using I-TASSER server. Supplementary Table 1. Identification results of the selected 1D-PAGE band and 2D-PAGE spots by [file 12870_2023_4149_MOESM1_ESM.pdf]

Supplementary Figures

Supplementary Figure 1:

The phenotypes of mock- and *R. microporus*-inoculated rubber tree

1.1) The aboveground phenotypes of the representative tree of mock-inoculated (control) and *R. microporus*-inoculated at 10, 30, and 50 DAI

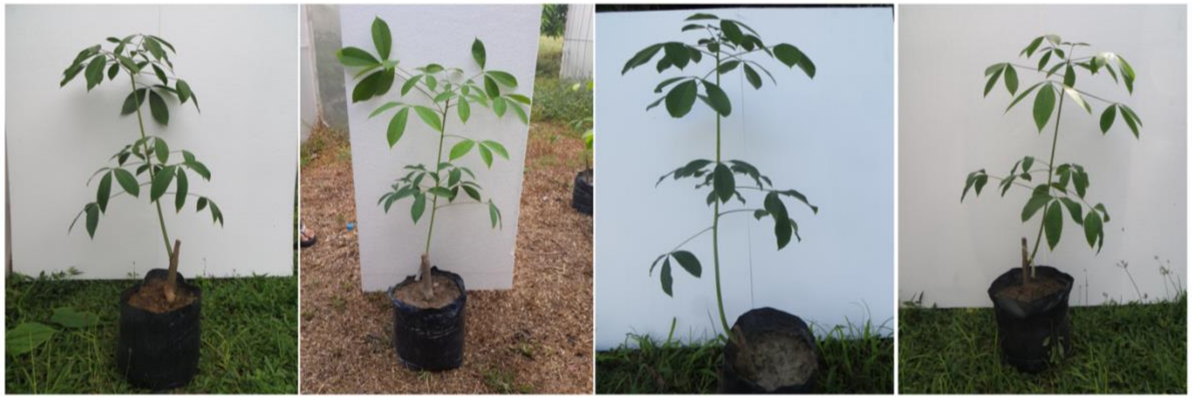

Mock-inoculated plant (50 DAI) | *R. microporus*-inoculated plant (10 DAI) | *R. microporus*-inoculated plant (30 DAI) | *R. microporus*-inoculated plant (50 DAI)

1.2) The underground phenotypes of the representative tree of mock-inoculated (control) and three biological replicates of *R. microporus*-inoculated at 10, 30, and 50 DAI. The yellow arrows indicate the white rhizomorph of the fungal infection

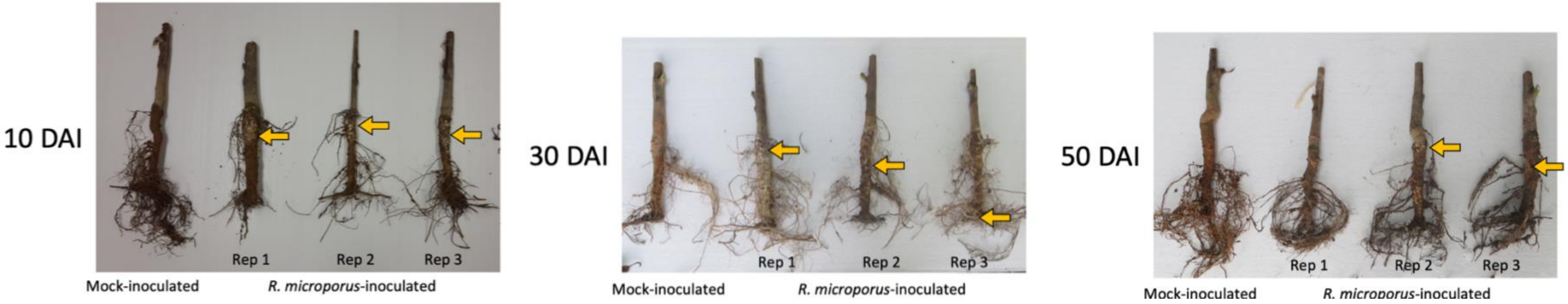

Supplementary Figure 2:

Raw Image of One-dimensional (1D) SDS-PAGE gel showed the comparison of the protein patterns of rubber tree leaves after *R. microporus* inoculation by SDS-PAGE with Coomassie brilliant blue (CBB) dye. Lane 1: protein marker; Lanes 2, 4, 6: mock inoculation at 10, 30, and 50 days after inoculation (DAI), respectively; Lanes 3, 5, 7: *R. microporus*-inoculation at 10, 30, and 50 DAI, respectively.

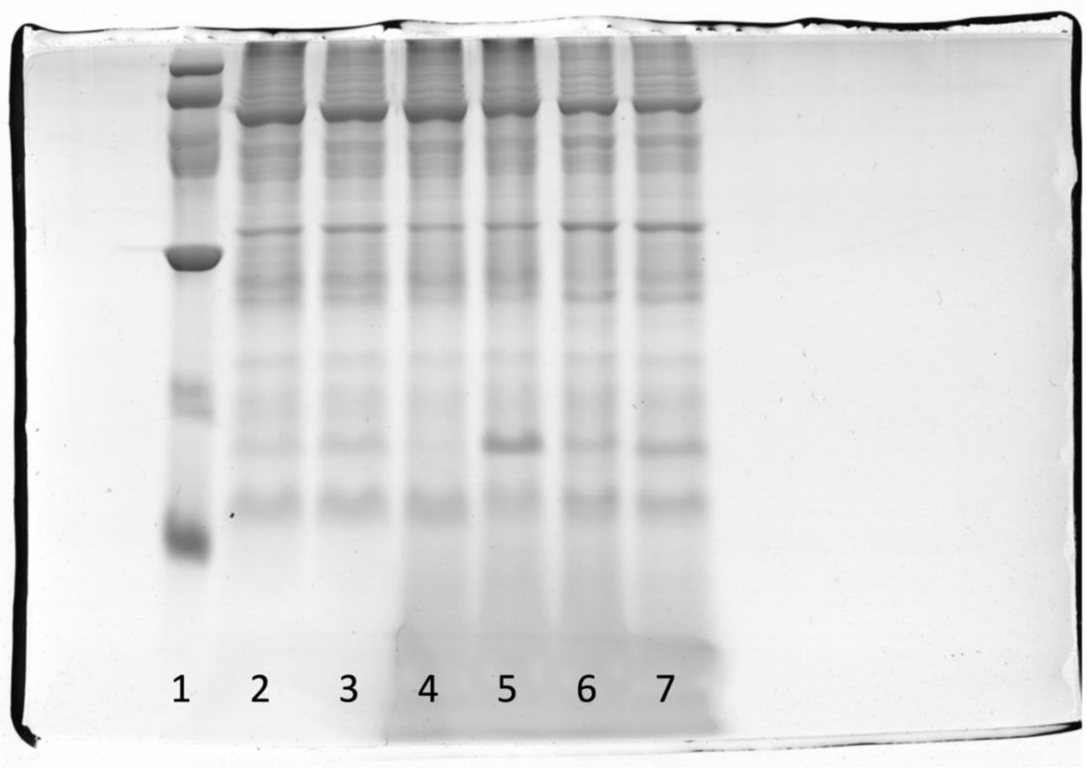

Supplementary Figure 3:

Raw Image of Two-dimensional (2D) SDS-PAGE gels. The three replicates of 2D SDS-PAGE of each treatment are showed below. The framed gels are the representative gels of each treatment.

**10 DAI mock-inoculation**	**10 DAI *R. microporus*-inoculation**
Rep 1 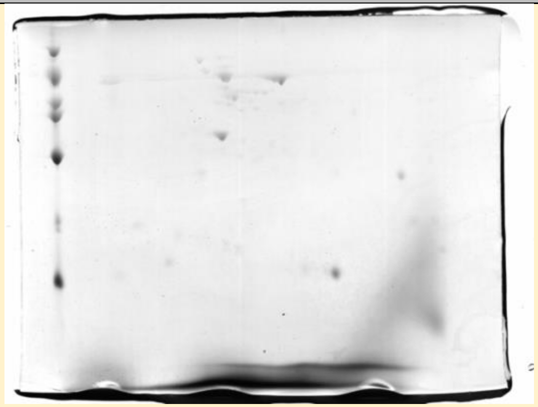<br>Rep 2 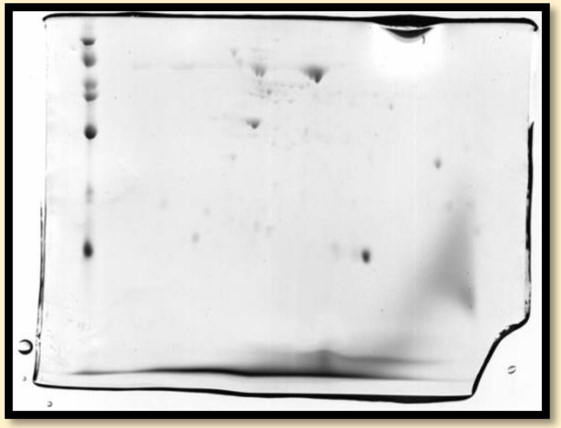	Rep1 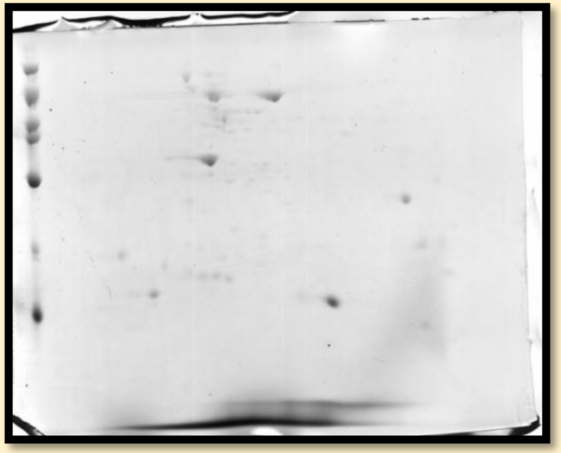<br>Rep 2 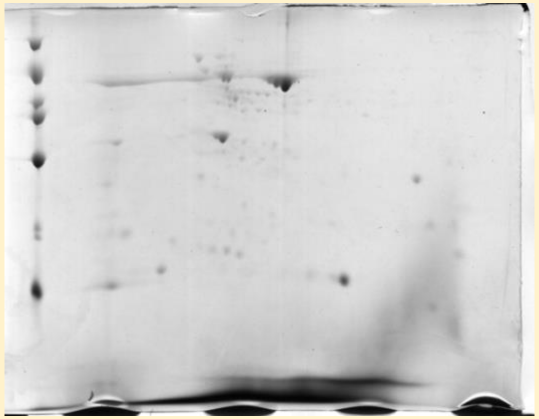

**10 DAI mock-inoculation**	**10 DAI *R. microporus*-inoculation**
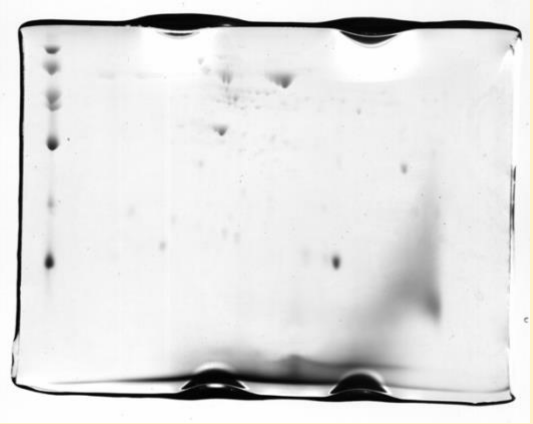 Rep 3	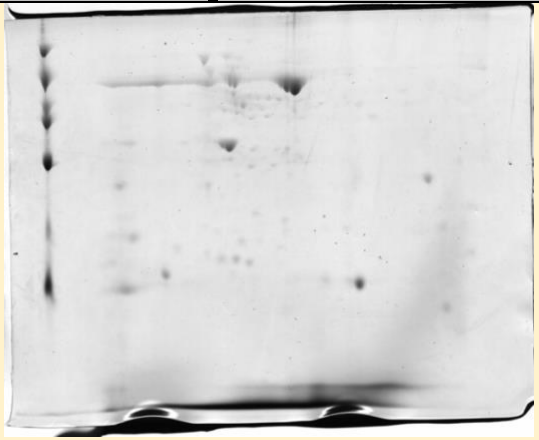 Rep 3

**30 DAI mock-inoculation**	**30 DAI *R. microporus*-inoculation**
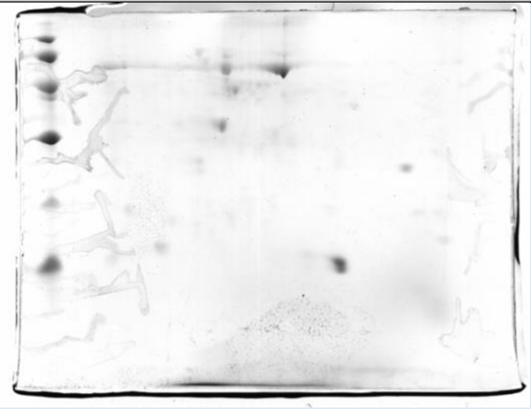 Rep 1	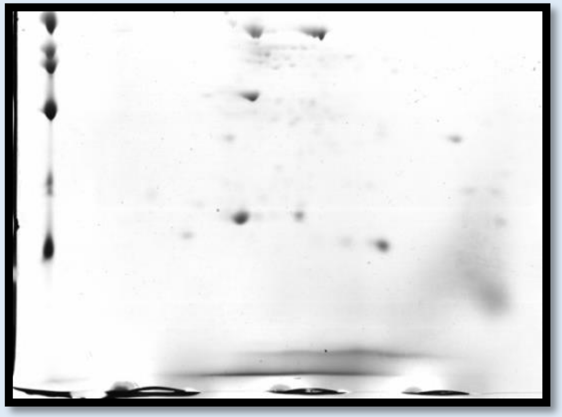 Rep 1

**30 DAI mock-inoculation**	**30 DAI *R. microporus*-inoculation**
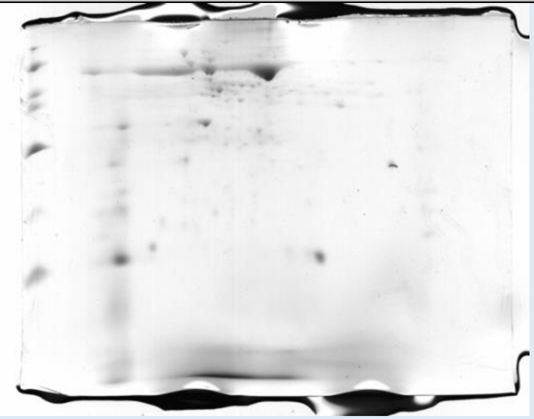 Rep 2 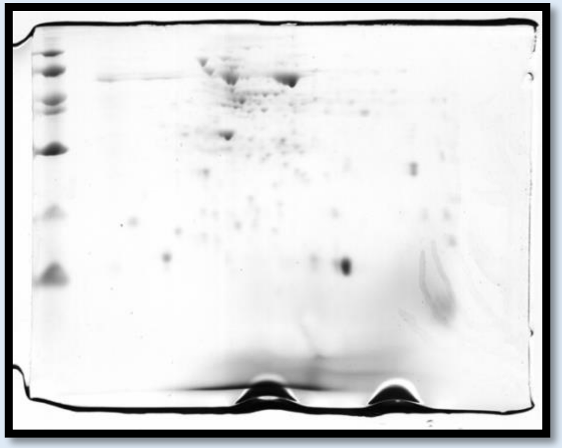 Rep 3	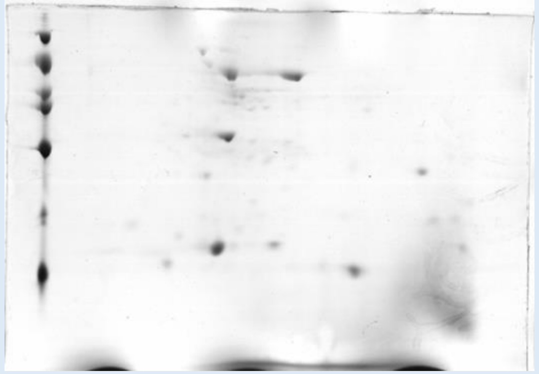 Rep 2 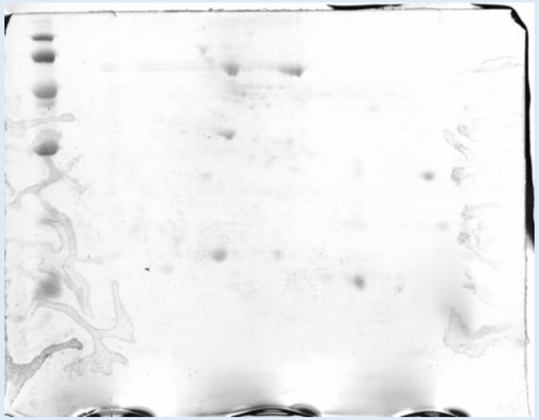 Rep 3

50 DAI mock-inoculation	50 DAI *R. microporus*-inoculation
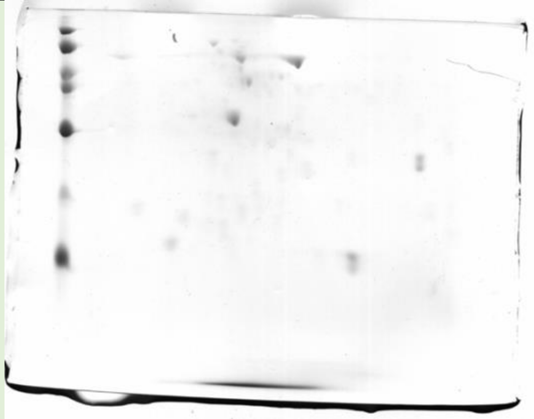 Rep 1 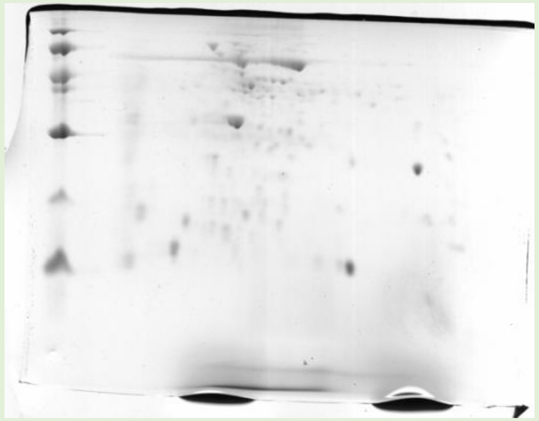 Rep 2	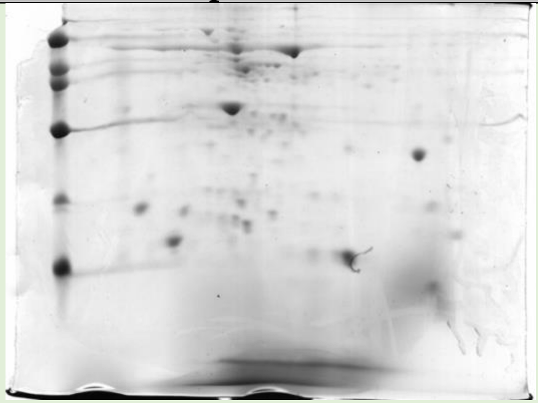 Rep 1 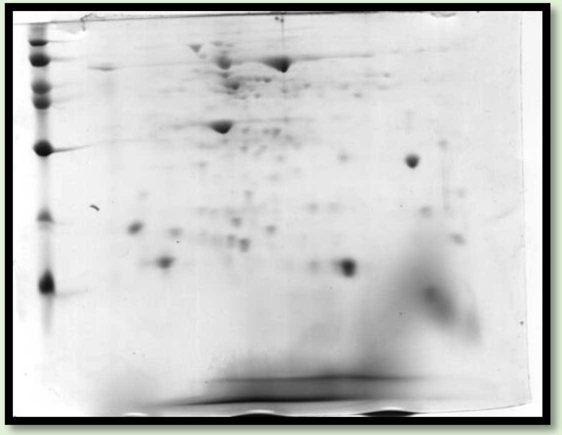 Rep 2

50 DAI mock-inoculation	50 DAI *R. microporus*-inoculation
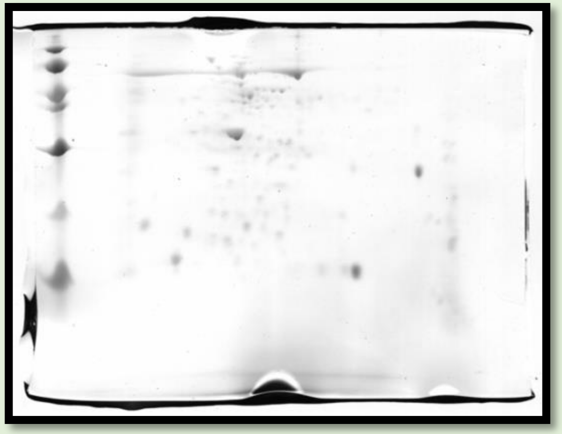 Rep 3	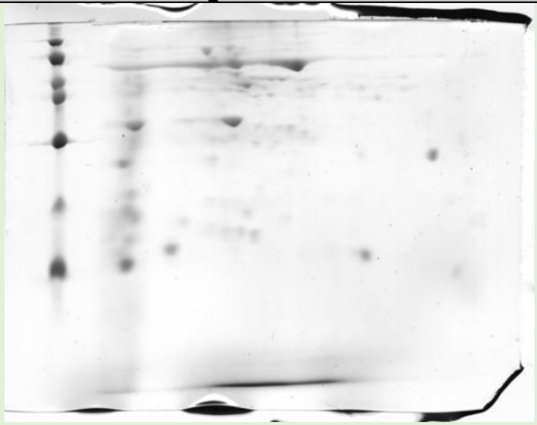 Rep 3

Supplementary Figure 4:

Location of the coding sequence of the uncharacterized protein LOC110748447 isoforms on whole genome shotgun contig LVXX01001672.1 (Range: 61922-78548)

Hevea brasiliensis cultivar reyan7-33-97 scaffold1672, whole genome shotgun sequence (matched 100% +/-)

```
(reversed seq)

78,724  GTGATTTCATTATAAAGCATAATATTTGGAATTTTGATTAATTACACTCTATTACAAGTAAGAAATTTCCCAATTGGAATACGTTAACATCATGAATTTATTAATTTTTGGCAGGGCTTCTTGGTTCTCT    78,595

78,594  CTCACCTATGGCATTATATATACTTGTGTATGTTGCCTCTGGACGCATATCTATCGATCATCCAAATAATTCTCTCTTCATTTGCTTTGCTTTGTTTTGTTTCTCTGTAAGCGAAAATGGCATTTGTCAC    78,465
                                                      Isoform X1 ATATCTATCGATCATCCAAATAATTCTCTCTTCATTTGCTTTGCTTTGTTTTGTTTCTCTGTAAGCGAAAATGGCATTTGTCAC
                                                                    Isoform X3 TCCAAATAATTCTCTCTTCATTTGCTTTGCTTTGTTTTGTTTCTCTGTAAGCGAAAATGGCATTTGTCAC

78,464  CGCCACAGCTGGTGTTCAAGTTCTGATCAAGAGTCTACCAGACACGTTCTGGACTAAAATTCAAGATTCCGCAAATTCTTTTCCTGAAGCCGCACCAAACCTATATTCATCAATTGCTGTCAGCGGCACG    78,335
        CGCCACAGCTGGTGTTCAAGTTCTGATCAAGAGTCTACCAGACACGTTCTGGACTAAAATTCAAGATTCCGCAAATTCTTTTCCTGAAGCCGCACCAAACCTATATTCATCAATTGCTGTCAGCGGCACG
        CGCCACAGCTGGTGTTCAAGTTCTGATCAAGAGTCTACCAGACACGTTCTGGACTAAAATTCAAGATTCCGCAAATTCTTTTCCTGAAGCCGCACCAAACCTATATTCATCAATTGCTGTCAGCGGCACG

78,334  GGGGTACGCAAAGTCGAGTATGGCCCAGGTACGTACTTGCTCCAAGATAATTCTTTAGCACTTCTTTGGTCTTTTTATATACATACTTGAAAAAAATGTTAATGAGTAAATTAATAAAGAATTAATGTTA    78,205
        GGGGTACGCAAAGTCGAGTATGGCCCAG
        GGGGTACGCAAAGTCGAGTATGGCCCAG

78,204  GTGAATTTGTTTTGTATGTAGATTCTCAGAATATCAAAAACTCGTCTGAGCTTATCACTGAAAACAGTCGGGGAAAATTAGCTTACACTGTAACGGATGGTGATATCCTCCAAAAATATAAAGTCAAAAC    78,075
                                ATTCTCAGAATATCAAAAACTCGTCTGAGCTTATCACTGAAAACAGTCGGGGAAAATTAGCTTACACTGTAACGGATGGTGATATCCTCCAAAAATATAAAGTCAAAAC

78,074  CTTCCAGGCTGAAATAATTTACCCAAATACTGGCAAATGGGTGGAATGGATCTGGACCTATAACTATCTCCCAGAGGATAAAACAAGGATTCTTGAACTTGATGAAGAGATTGCAGTGATTACAGTGAAG    77,945
        CTTCCAGGCTGAAATAATTTACCCAAATACTGGCAAATGGGTGGAATGGATCTGGACCTATAACTATCTCCCAGAGGATAAAACAAGGATTCTTGAACTTGATGAAGAGATTGCAGTGATTACAGTGAAG

77,944  ACCCTGGCAAAACTTGATAGCTATATTCAAAACAAGTTAACCTAAATTGCATGGATGACCTTCAAGCACATATATATGTATAGTATGTAAATAAAACAGAGCCTTGCTCTGCACTCATCCATGCATGTCT    77,815
        ACCCTGGCAAAACTTGATAGCTATATTCAAAACAAGTTAACCTAAATTGCATGGATGACCTTCAAGCACATATATATGTATAGTAT

77,814  GCTAGATTATTGTGTCTGTGTGTGTACCTAACAATGTGCTACGTGTGGTGTAGTAATAAATAAAGCAGAGTCCTATGTATGCTTAATGCTCTGCACTTTATCCTATAATTGTCTGCGTTATGCTATGTAC
        AATGTGCTACGTGTGGTGTAGTAATAAATAAAGCAGAGTCCTATGTATGCTTAATGCTCTGCACTTTATCCTATAATTGTCTGCGTTATGCTATGTACTTTTCTCCATTAAAATAAAATCCAGCTTTAGC
        TGGTTCATGTGGTATTTGCTTCGACCTTATGTTTCCCATTTCACGAACCCAAAGCAAAGGACTTAAAGCTTGTTTGCCAACTTGCTTTGCATAATTAATTCAATAAGGGCCAATTGTTATACATTTTTTT
        TTAATTCATAATAACTTAATTTATACTTAATTGAATGATGCACAAAACATCAATATAGTTTAATCAATTATATAAGGGACATTAGAAAAAAAAAAAGCTATCATCAATTCGTCAAAGCATAAATGCATGG
        TAATAATAAAAATTGTTAATAAGAAAATAATTAATTTGAAAGAATGATTTTGAATAATAAATAAGCTACACATTAGGATAGTTTTCTAGTAATTTTTTTTCTATTAAAGATAAAGTTCATTGCCATAAAA
        AGGCCTGGGTCAATTCAAATAAAAGATTCAGACGGGATTCAAGATACATAAGGAAACTCAACCTAATCATCAGCTAGTTAAACCAACAAAAGTAAAATACAAACAAGATCAAGCTAGCATAACGAAGGCC
        TGTGGAAAATGGCTCTAATAGGTTGCAAACCCACGCCTGAACTAAATAAGGCCAGAAGCCCAGAAAGAACCCTGAGCACACTGGACTTGACCCAAAATAAGCAAGGATCCGAAGAAACAAATAAAGACTC
        CGTCATTAACCGTCAAGCTTAACCGAGCTACTTCAGGGAATCTAAGAAAGCCAATGAAGCATCTGATTCTTGTTAGTAGCCAACGACCACCATAGTTGAACCTCAAAAGCACCCTGGGAGGGAAGCAAGC
        TTCTTAGAGTCTTTAGATAAACGATGAGAGTACCATGAAACCAGCACCTTGGTAAGAAGTAGAAATTATAGAATAGGGAAACCAGATGGGCATAAGGCAAAGCTCTTTAGCTCTGTTGGCTGTTAAGGGA
        CTAGTTAGCAAGAAGATGGCACTAATAACATCTCCAAGAGGTACCCCATAGCTAAAAGTGAACCCATCCATCCCCAAGCTTCAAGGCAGCTGAGAGGCCAAAGCCGTTGCCAAGAGCATCCCCTGCATAC
        ATCTAGAAGACATAGACAAACGGATAGAGACTGTCATGACCCAAATTATGAGTTAGACCGGCACTAGGACTTGGGTCAGTATAAGGCCCCCAAAGCCCGTAGTAAATCTAACTATTCCTAGCCCAGCTGT
        AAAGCCCAGCACTGTAGTCCAATTTCAAGAAAATAAACAGACAGAGTCCAGTCATAAATTGAGCTACCCAATGGAGAGCAATATCTCACCCGACCTGTAATCACGACACAAATCAATTTGGGGAGCTCAG
        CTCACCCTTACAATCTACAAACAAATATATATAATCCAATGGGAGCTCAGCTCTCTCATCCAAATACAATCACATAATAACTTTAAAAGACTACTTTGTCAATTTTTTTTTTTTTAATTATTGATATAGTT
        TAGTTAAAAATTCAGTCTTAAACGGGTTGGACTTACCAAATCCCTACCCGATCTTATATGGATCAGACCCAGTTGCGAATTAGCCCGAGCTTGGCTCAGAACAAGTCAAGCCATCTTTGATCTCACAAAG
        ATCAGACATACCATGTATAGATTGGATTATAATAAACTAGATTAGGAGATCGATGCTACAGATCTAAGATTGGAATGACGTGGACATATTACATCTCTGTAATGTATGACCAAAGGGAGTTACATTGACA
        CAGGTACATTTTTGTTACATATGGCTAAAGGAGAGCACAGGATTAGTCAATAGAATCCCTTGAATCTCTTTGTTGCTCTTTTTTTGCCTTCCCTTGAATCTCTTTGTTGCTCTTTGCTCCGTTGTTTCAT
        TTTTATTTATTTATTTATTTTTGGTTTGGGCTGAGTTTGCTTCGGCCTCTCTCCCAATACCATTCTTTAATAAAATTTTGCTTGTTTATAAAAATTTAATAAAAAAATTACAGTAGAAAAGAAGCATAAA
```

TTTTTAAAAATTGAGAAAATAAATATAAATATTCAAACCGGAGTAGATTTGACCCTAATTGATAATAAAGATAAATAAAGCTTAAAAAGTAAGGAAAAAAGAAAAAGAAAAAGAAGAAAAAATGGAGTTAT
TATTGATGAGAAAATTTCATTAGTAACTATTCAAAAGAAAGAGGGAGTTTTAAGAAGGATGAAGGAGGAAATTTTATAAAAAGAATGATGAGTCTTAATTTATTTTGGCAGTAAGCCCAATCTTCTTTTT
TTTAGTTAATTGAGGATCACAGTACGATATAATAGCTGAACTTCCATCTAATGAATTTGAGGAGAGGAAACCTGGAACTGGCTTATGAAAAATATTTTCAATAAAATTTACTTATGAAAAATATTTTTTA
TAAATTTTAACTTTAATAAAAATAAATATATTTCATAATTATAAAAATTTTATTTTATATAACAAAAAGACAAGTATTAATAACAAAAATTTTACTTTTAATTTTTCATTAAATTAAGGTTTTAATTTAA
TTTTTTAAGTAAAACGTTTATATTTATAATCTAACCAAAATATATTTATTGTTTATAAAGAATTCATTAATTTTAAACCAAGTGTGAAAAAAGAAAAAACATATATATATATATATATCAGATAATGGCG
ATTTGTGGTAGGCAGATTTATATTCCATTTCACAGTTCTATTGTGAACAGCTGGCACATTGTCCGCCTCTGTGGTCAAGTGACAAGGGCACAATGAACTTACGTCCATGTCCTCGGTTCGCTCCCCTTCT
CCTCATGTGCTTATCGGAAAAAACAAAAGAAAAATGCTGGCACCTTACACCGAATGGAATTCCATCATCCTGATTTTTAGAGAAGTGAATTATTACGCAGACTGACAAGTGCGTGTAGCACTCGTTTGCT
TCGATTCCATTTTATAAGATTTTTTGAATTCAATTTAATTTCATATTTTGTGTGTTTAGTTTATTAAAAAATTTAAAATTTAATTTTAAAATTAATTATTGTTAAATTTGATGAAATTTAAAATAAATTC
TATAAATCTTATAAATAACATTTTTTTTAAAATTAAAAATATCCTTATCAAAAAATTCTCTTAATAATTCATCGTTTATATACACATATGTTGAAATTATACATTTTAAAACTCTAATCACTAATATGGT
TAATAGAATTTAAAATCCTCATTAAGTACTATTAATTAATTAAAAAATTAAAAGTGATCAAGTACTATTAATATATATATCATCCTTAAATTCTTGTGAGAATCTTGTGAGAAATATGAATTTATTTTATAT
TTACAACTATATTGTTAGTTGGTTTATATATTTATTAACTGTTATAAATTTATCAGATATAATTTTTTTTTAATTTTATAAAAACTTATAATTTTTTAATTTTATTTTTTTTAATAGAAAATTTGATAGT
TTATTAATAAGAATAATAAGATAAGTACATCACAATGTTCTCATAAGAGAAAAAAAAATACAAACATAGATAATTAAAATATTCTGTTCAATGATGATCTGGATAGAGCAGAATAGCCTTTCGAGTAGTAA
CTAATAATTTTTTATTTTGGACAATCAAAGAAAACTTCTGTAATATTACCCTTTTAAGGAATTTGTTAAATCACCACCATCAATGAAGGAATAGATCGTACCATTAGGCTCCATTGATAGAAAAATATTA
CCCATACCTGAGTCTCCAAATAAAAAAAATCAATAATCAATGATGTATTTTGAATTGCCAAATAGTCTATTCAAATATTTAAAAAAATAAACAAAATCTCACTAAGGGAAGAAAAATAAAATCCTAATAA
TGAGAAGAAATAAGACCATAATATTGAGAAGAAAGCTTCAGATTTATTCTTAAATGACACAAATATAAAATGAGAAACTTAAATATGTTCCAAACAGATGTAAATATAAGAGATTATTTAAAAAAATAAT
AAAAAAAAGAAAATCGATCTATGGGTAGTCACCTATGAAAAACTCACCGATAATGACCAAAAAAAGAGATCTAAAAGTGAAAATAAAAAAAGAGAGAAAAGAGAGGAGGAGAAGAGAGAAGGAGAATAAA
TAAATTTTAATTTTGAATAGTGTGTGTGCAAATAAGACAAACTAGATAACATTCTCTAGAAATAATTTTTTATTTTGGACAATCAAAGAAAACTTCTGTAATATTACCCTTTTAAGGAATTTGTTAAATC
ACCACCATCAATGAAGGAATAGATGGTACTATTAGGCTCCATTAATAGAAAAATATTACCCATACCTGAGTCTCCAACTAAAAAAAATCAATAATCAACGCTGTATTTTGAATTGCCAAATAGTCCATTC
AAATATTTAAAAAGATAAACAAAATTTCACTAAGAGGAGAAAAATAAAATCCTAATAATGAGAAGAAATAAGACCCTAATATTGAGAAGAAAGCTTCAGATTTATTCTTAAATGACACAAATATAAAACA
AGAAACTTAAATATGTTCTAAACAGATATAAATATAAGAGATTATTTTAAAAAATAATAAAAAAAAGAAAATCGATCTATGGGTAGTCACCTATGAAAAACTCACCGATAATGACCAAAAAAAGAGATCT
AAAAGTGAAAATAAAAAAAGAGAGAAAAGAGAGGAGGAGAAGAGAGAAGGAGAATAAATAAATTTTAATTTTGAATAGTGTGTGTGCAAATAAGACAAACTAGATAACATTCTCTAGAAATAATGGTTGA
ATTTGTGAATAACGTTCTTTTATTTCTAGAAATAAGGTCGAATTTGTAAGGATATTTTTATGAAAAAAAAAACTGTAAATTGTTAATTATAGATTTTCTCTCACAATAAATTACATTAGTAGTAAATTAA
TATTAATACCATTGATGGCTAATTCAGTTTTGATAAAACTCGATTAATGTTTCCAATACGAGAATTTACATTTTAATAATTATTTCTCTTAGTGTGATATGTAATATTTTATTTTTAAATTAGAAGAATT
TATTTTTATTTAAATTTTTTATAAAAAAATAATAAAGAATCTCATTTAAAAAAATATAAATGTTTAAAAAAAAATCACTTTGATACTAATTTTTTTAGCAAAATGGATTTTGAATTTCACATCTCTTTGC
CATTCGATCTAATCATGGTGATAGCCAACTAAGCTCCCTTAATTGGGTAGAATTAATGAAAAATAATTTTAATTTAGGCTTTATCTCTTTCGTAGAAAATATCTTTTTTAAATATTTTTATATTTTTTAG
TATTTAAGCAATTAAAAAAAATTGATCAACAAAAAATATTTTTATAATTTAAAAAATTTAAGTTAATTTAAAAAAAAAATGGCTCTCTTTTAAAAAAAGAAAGTCATTTTCTAAATTTTGACAATCTAATTA
AATTATAAAAATACTTATATATGCATTATATAAATATATATTATTAATTTAATATTATAACTAACCAATGAAAAATATTTTCATTAAAATATTTTCTTAAAAAATATTTTTCATAAAAATATTTTTCATA
TAAAATTTATTTTTTATGAAACAAACTCTAAGATATAAAAAAGATCATACTTAATTTTTAATATTAATTAAGTGACTTATTACGAATTTGAAAATAGTACAAAAAAAAATCTCTCTCTATTTCTTTTTCT
CTTGAAAAAAATCTTTTAATCCTATTTTTAACTTGGGACTGCCACTGTAAGCTCAAATATAACCCCCTCTATTTCTTTTTCTTTATTTGCTTTCGATAAACTCTTATCTGCTTTCTTTATTTGCTTTCAAT
GAACTCATAAACTTAAATACCTTGTAGTTATGCTTTAGTTGTTTGTTTTGAGCTTTAGCTCCCAATTTCTACCAAAAAACTCAAATATATATATATATAATAGAGGTGCTTTCTACTAAAAATAAATAGATT
TGCTTTCCTTTCTCTTATATTCTATGGAAATTTTTTTCTCCTCTTGAGTAGATTTAATTTCCTATGTTGTTGTCTTCTTCTTTCTGATATTATTTTTTTAATATTTTCTATGAAAAGATATTTGTTAATTT
TGAATTATTAATTATTTATATCACATGAATATAATTTTTTAAAAGATTATAATAATTGTGATGGAAAATTCTACTTAAAAAAGATATTGAGATATCGAAACAATCTAAGAGTAGTACATAATGATCATAT
GTAGCATTTTTGTCCTATTTTGTAAGGTTATATCATATAATTTTTTTATATTAATGTTATTTATTGTAAATATCACATTACTATTTGATTAATTGAACGATACTGTTTTTAGTCTCTTTTTTTATAAGCTT
GTTGAAAATTATATATAAATTTAATTTTTATCGATGTTAAAATGTTAAACAAAATTTATTAATTTGTTTAAAAAAACGAAATTTAAAAAATATATATTCTATGAGAATTTGAAAAATTATTAAATAACGA
CCCGGAAAAGCTCATTCTTAAAATAATTTCTATACTTAAATAAAATATAATTTGAAAATGAATACATTTATTTTATTTTTAAAACTTTTAAATAATAAAATTCGAATAAGTTTATTTAATATAAATAAAT
AAATAAGTTTATTTATTTATGTACTTATCATGTGAATTAAATAAATTTATTTTTTTAACATAATTAGTTTTTATTAAATTTTAAATTTAATACCTCACAATCTTGTAAATAATTTTAATGTTATTAAATT
ATAAAATTTAATAAATGGATGGTGTAAAAATATAATAAAATGTGAAACTTATTTTGAATTGTTGACGTAACATTAATTTGGAAGTTAATAAGCGCGTCTCACGCTTTGTCTCACTAGCGGAAGGGAACAA
AAATTTTCTACAGAATTTTTTTTTTTAAATAAAATTTTTGTAACTATTAGCATTTAGCAGAGCCACATACGATTAATGTGATGCTTACGTGCATGGCATCTCTACATAATTGCATGGAAACAGCCAAAGA
AAATCCTTTAAATGAAAACAAAATATGATAGAATAATTTGGAGACTTAAGTAATGGATAAAGACATAAAAATTAAAACTTTTTCTTTTATATCTTTTATTTCAAATTAAAAAAAAAACTTTACAAAATAA
TTTATAAAACAAAAGTTTGAGATGAACATATATAAATTTAAAATTAGTGTATATATATTATGCAAGACACAGCATTTCGCAATTTTAGATAGAATAAATTACGTGGTTGCTAAAAGAGAAATGAAAATAA
ACATGATTAAATTACGTGGCTGTCTATATTGTTTACCCTCTTCCCGTAAACGTCAACATTTCAAAGGAATGGCACTTGAACTATTCACACTCGGCCGGTAAAAATCATATGATGGGATTTTATTAAAAAA
ATTAATTTAAATAAATTTATAAAAAAGAGAGAAATAATATCAACAAAAGAAAAGAAAATTGACGACCAAAAGAAAATATGAGTTTTTATTGCTGTTGCATTCGAGGAGTCAATATGATGATTCTATTTTG
TTTATCCTCTTTCTAGAAAAAGTCAACTTTTCAAAGGAATGGCACTTAATGTATTCACATTTGGGATGGATGGCAATTTGGATTCGCCCAGTTGTGTTTGTATCCACATAAATAAGAATGAATCGTGAGA
AAAATCTAAATACGAATATAATTATTTTATAATAAAAATGATATGATTTAATATGATTTATATTAAATTGTGTTGATTAATTTGACATAACATAGCTTATTTAACACTGTTAGATATAAATTAATTTATC
TAACACGATTTGATATAAATTTAATTTAATTTTTTATAAATATGTTGAAAATTATATATAAATTTAATTTTTTATCGACGTTAAAATGTTAAATAAAATTTATTAATTTATTTTAAAAAATGAAAATTAAA
AAATATATATTCTATAAGAATTTGAAAAATTATTAAATAAAAACCCGGAAAAGCTCATTCTTAAAATATATATATATATATATATATATATATATATATATATATATATATATATATATATATATATATATA
TATTATTCTAACCCTGGTATTATGAACTCCGTGTATATGCTTTCATTTCGTGAAAATTCGATCGTTGCCCGGATCTGTAATTTCTGGTCGAGCAGATAAGCTGCTGGAACCATTTCAGAACCGGATCAAG
ATTATAGGCTATCCCACCGTTTTCAGACGTTCTAGACGCGTTCCAGTTGTCAGATTTGATATAGGTAAACTTGAACTCTATATTACTCAATTTTTGCCTTGTACTGGGTTAGAATAAAATTTATAAAATA
TTCATGAATGATAAGAAAATTACGATTCCTATTGCAATAGCCTTATAATATTGTTAAGGACTGTGGGGCAGGTTTATAGAATTTTTAAAGTTAGTTTGGATAGTTTTTGAAAAATATTAGTTTTGAAGTC
TTATAATTGAATTGTCTGATATTGTGATTATTGCTTGGTTTGGAGGGCCTAGGAGGGGTCATATAATGATGATGAGATATGGATTGAGTTAAGAAGTATTATTTGAACCATTTTGCAGGTTGGGTAGGTC
ATAGGTATAGGGGAGACTCTGTCGGATTTTCAGCACAACTTAGGGTATCTTTGATCTTTTCTAAAATTGTATTGAGTCAAATATATTAAATAATTGCAATGAAATTGTCAGGTGAGCCAGGACAGCCTTC
CTCCTTCGTCCAGCCGCCGCAGTGACCTCGGTTCAAATTTGTGAGTAAAATATTAATTTTAATTGTAATTTCGATATTATTATATATTCAAGCATGCACATGCATCACTTATAAATATGTATCTATGTAG
TTAAACACTAGGCACATTTTATATTGCATTCATAATTGTTGAAGTGCCATGGATATTGTTTGTGGTAATTTGGAGCAGTGTCTGTGCGTGGCGTACGTGTGGTATGGTATTAGATATGGACATGATGGGT

```
AGAAATGGTTTGAAAGACACTAGCTGGGACCCCGTATTTGGTTTATTAAGCGAAAGTCCGGCATGAAAGACACTCGCTGGCAAATATTGGATTAAGAGAGCTGTGTAGGGGATCAGTTCCCATATATGTA
TTGCTTGACAGTGTTGGGTGTATGAGTGTTCCAAATTGCCTTTTTGCTATTATGGTATGAAAATTATGATGATGTTGCATTTCACTCCACATGGTGCATTAGCTTTAGATAGTTATAGAGATTATGATTA
AAATTTGTATTTTACTCTCTGAGTTGAACGCTCACTCCTGTTTATCTATTTTTTCAGGTTACAGGAGGATCATTTATTGTGGCTAACCTGCTCTTTTTCTTCACAGGTCCATTAATAGAATTTAATGTAT
TTTGTACAATTGAGTTGAATTTTAGACTCCGTATGTGTTAGAAGCACTTATTTTTATTTTGTGTCTATATTATAAAAGTTATGTTGGACTTGTAAAATTATTAACTGTATGCATGATGGGATTGGATGAG
GGAGCTGAGCTCCTATTTGAGTTATGACATTTTGATTATGTGGAGGGTGAGCTGAGCTGAGCTCCCCAATTTATTATACATTGTGTTTATAGGTCGGGTGAGTCAAAAACTCTCCGTTGAATAGTCCATT
TTATAGTCGGACTTTGTCCGATTGAATTCTTGAAATTGGGCCCAAATGGGCCTTAGAGTTGAGTTGAGGGATAATTAGCTTACTACCGGCCTCAGAGGCTTTAGGCTGGCTCAGGTCCTAGTGTCGGTCC
GGTCCATAGGTTGAGTCGTGACATTTATGCACTTATCATGTGAATTAAATAAATTTATTTTTTTTAACATAATTAGTTTTTATTAAATTTTAAATTTAATACCTCACAATCTTATAAATAATTTTAATAT
TATTAAATTATAAAATTTAATAAATGGATGGTGTAAAAATATAATAAAATGTGAAACTTATTTTGAATTGTTGACGTAACATTAATTTGGAAGTTCATAAGCGTGTCTCACGCTTTGTCTCACTAGCGGA
AGGGAACAAAAATTTTCTACAGAATTTTTTTTTTAAATAAAATTTTTGTAACGATTAGCATTTAGCAGAGCCACATACGATTAATGTGATGCTTACGTGCTTGGCATCTCTACATAATTGCATGGAAACA
GCCAAAGAAAATCCTTTAAATGAAAACATAATATGATAGAATAATTTGGAGACTTAAGTAATGGATAAAGATATAAAAATTAAAAGTTTTTCTTTTATATCTTTTATTTTGAATCTAAAAAAAAAAGTTT
CAAAAAAATTTATAAAACAAAAGTTTAAGAAGAACATATATAAATTTAAAATAAGTATATATANNNNNNNNNNNNNNNNNNNNNNNNNNNNNNNNNNNNNNNNNNNNNNNNNNNNNNNNNNNNNNNNNNN
NNNNNNNNNNNNNNNNNNNNNNNNNNNNNNNNNNNNNNNNNNNNNNNNNNNNNNNNNNNNNNNNNNNNNNNNNNNNNNNNNNNNNNNNNNNNNNNNNNNNNNNNNNNNNNNNNNNNNNNNNNNNNNNNNN
NNNNNNNNNNNNNNNNNNNNNNNNNNNNNNNNNNNNNNNNNNNNNNNNNNNNNNNNNNNNNNNNNNNNNNNNNNNNNNNNNNNNNNNNNNNNNNNNNNNNNNNNNNNNNNNNNNNNNNNNNNNNNNNNNN
NNNNNNNNNNNNNNNNNNNNNNNNNNNNNNNNNNNNNNNNNNNNNNNNNNNNNNNNNNNNNNNNNNNNNNNNNNNNNNNNNNNNNNNNNNNNNNNNNNNNNNNNNNNNNNNNNNNNNNNNNNNNNNNNNN
NNNNNNNNNNNNNNNNNNNNNNNNNNNNNNNNNNNNNNNNNNNNNNNNNNNNNNNNNNNNNNNNNNNNNNNNNNNNNNNNNNNNNNNNNNNNNNNNNNNNNNNNNNNNNNNNNNNNNNNNNNNNNNNNNN
NNNNNNNNNNNNNNNNNNNNNNNNNNNNNNNNNNNNNNNNNNNNNNNNNNNNNNNNNNNNNNNNNNNNNNNNNNNNNNNNNNNNNNNNNNNNNNNNNNNNNNNNNNNNNNNNNNNNNNNNNNNNNNNNNN
NNNNNNNNNNNNNNNNNNNNNNNNNNNNNNNNNNNNNNNNNNNNNNNNNNNNNNNNNNNNNNNNNNNNNNNNNNNNNNNNNNNNNNNNNNNNNNNNNNNNNNNNNNNNNNNNNNNNNNNNNNNNNNNNNN
NNNNNNNNNNNNNNNNNNNNNNNNNNNNNNNNNNNNNNNNNNNNNNNNNNNNNNNNNNNNNNNNNNNNNNNNNNNNNNNNNNNNNNNNNNNNNNNNNNNNNNNNNNNNNNNNNNNNNNNNNNNNNNNNNN
NNNNNNNNNNNNNNNNNNNNNNNNNNNNNNNNNNNNNNNNNNNNNNNNNNNNNNNNNNNNNNNNNNNNNNNNNNNNNNNNNNNNNNNNNNNNNNTTGCAAAACAATATAACTTTTAATTTATTATATAAA
TTCAAAAAATAAATAAATTTTAATAAACAAAATAAAATTAACTATAAATGATTAAGTTTATATTTATTAACTTATCATTTTTTAATAATATAATTTGTGAGTTAATGGTTAAAAATGGGTTAAATTAGCA
AGTCAGGGTTAACATGTGAAAAAAATAAAAAAAAAACGTCATAGGTGTGTCAAGTTGGATTAGTGGATCAACTCGTGACATGACATCATGTCATAAATAGATCGATACAATTTCGACATGAATATGAATA
AACTCAATTTAAACCCTATATTTTCATGTCATATCACGAGTCATAGCTAAAATTGTCACCCCTATTCACACTTGTTCAATCAACTTTTTCCACAAAATGATACCATTTAGTAAGAGTGTGTGGTTTTAAT
TTCAATTTGGATCTAGCTAGAGATTATAATTCAATTAAAATTTTGGTATAATTTTGATTTATTGGATCTAGCTAGAGATTATAATTCAATTAAAATTTTGGTATAATTTTGATTTATTTTTTAATATTTT
AATTTTAATTCGATTCAATTTGATTATAATTATTTGGTTTTGTTATGGTTCGATATAATTTAGGCTCAAATTTCAACTTTTAAAATAATTTTATACAATTATTTCTATGAAATTCATACTTAAATTACCT
GTTTAACCTTAAAAGTTTATTATTAATACACAATATATCACACAATATGCTTTTTAACTATTTCTTAATATATAATCTTTAACCTCCCACTATAATATAATTTTGGATTAAGTATATTAATAATATAGT
AATAAAAATATATTATATGGTATACATTTTAGCTATTTATTAGTTATATAATCTTTAATCATAATTAAGAATTAAATATATTATATAATATAGTAGAACACTAATAATTAAAAATTATAAGGTAATTTAA
TGTATTTATAACTAAAATTATTAAAAAAATAGAAGAATGATAGATAATATTAAGTTATATACAATCCATAATATATATACCATTAAAATATATAATATATTTATGAAAATCATTAAAAAAACATATGTAC
GATTCATTTTTGATTCGATATAGGTCCAACATTGATGTACCGTTTAAATTCGATTTTTAGAGAAGAACTAAAACTGAATAAAAAAATCGATTCAATGCAATTTTTTGATTTGATTTGAAAAAGTGGTGGA
GCTATAATTTTTATCAATGGGAGCAAAAGTTTTTTTATCTTGTATTTATAAATTATTTAACAAAAAAATATATTTTTTACTTACAAAATATTGACTTAATTATAAGCATATGAATTAATATACTTTTTGA
TAAAATTTAATTATGTTAAAATTTCAATTTATTAAACTTTGTCAATGTACAAGATTTAATTATATCAATCATTATATCCTCACAAAAAATTGAAAAAAAAATTATATATTTATTTTATATGTTGTCTTTA
GTTTTGTTTTTTTAATCAATATGTTGTCTTTAGTTTAGTAATTACCAATCAATTAAAAGTAATATAGTGGAATTAAATATCAACTTTTTTCTCACTTTAGCTTATACATTATAATTGAAAAATTTTTAAA
TGTAATAATCTCATTGGAAGATTTTCAGATTATTTTATTAATAAATAATTAAATAATTATAAGACTATTAATATATTTCATATCAAATTTTAGTTCTCTCATATATAAATTTTATATAGACTAGGAGAAA
AAAAAATTCAATGGGCATCAAAATATATATAATCATGATAGAAACTAATAAAACAAAAAAATTTCAGTGAGTCAATTGCCCCTATTGGTGAGCTGTAGCAAAACATGTAAAAAAAAAAAGTTTTAATTAAA
ATATGAAAGTGGTCATATTTGAATTTCTGAATATTAATAAGTTAACTTATTATTTAAAGTGATGATATATTATTTAATGTTTTAATTTTAGTTGAAAATATGAAAAATTTAATTATAAATTTTTAGTTTT
CAATTAATTTATTTACTTAATTTTTATAATTATCATCTAAGCAATATTTATTTGTTGTTTAAAAAGAATTGCATAAATCCATTTCAAGTTTACATTGTGAATAGATGAAGTTCCATCATCCTAATAATGT
CACTATTATTATTATTATTATTATTATTATTTAATCTCTCTGTTCATATTTTCAAGAAGCGAACTGCAATCCTATCCCATAAAAAAAAAAAATAACACAACAAAAAACCTCTACAACAACGATAATCACGT
TTAGAGTAATTAGTCTTCCCTTCACACTGTAGCATACACATTCACTCAATTAATAAATGATATATTAATTAAAATTATGATAAATTTTTTATATAAGCATGTGTTTATGATTTTTTAATAATTGAATATA
TAAACACATTGGTGTAGTAAGAATTTTCATTCATATCCAAATTTTATTTCAAATAGATATAGATATTTAAAAATTATTAAAGAGGTTCAACAAAATAAACACTCTACAATAAAAAGATATATATCCTTAA
AAGTAGTCAAAATAGCAATAAAACTCCTCTAAAGGGAAACATAGCACTACATATAGATCAGAGGAAGTAATTATGAACTTTAGCTTTTCATTTAAGTCATTTACTTTTTATATTTATCATCTAACCAAAA
TTTATTTATTTATTGCTTAAAAGGAATTGCATAATTTTAAAATTTGGCAATTTCAGATATTGTAGACTTACAAATCATTTCAAGTTTCCATAGTGAACAGATGGAATCACATCATCCTTATGATGTCACT
GCGTATTTTATTTTCTCCTTATTCATATTTCCAGAGAAATGAATTGATTACTATGCAAACCTAAATGATGCTTGAAAATGATAAATAAGATGTATATATGCACACACTAATTTTTATATCAATTAAATTT
TATTTAATTATTTATTATGATAAGATTTAATTTTTATATCAATTAAATTTTATTTAATTATTTATTATGATAAGATTTGCTTTATCTACACATATAGATATCATATGATCTTTATTTTGAATATGTGATC
CAATAGCTCATTTTAATGTGGAAAATAATTTTTTGAACAAAAAAAAAAACTTATATTTATTCATTGAGCAATAAATTTGACGTCAACTTGAGGCAAAGTTAGGAGTTTCTTATAATTAAAAAAACTTATA
TATGTTTAAAATTTTCAAATTATCAGAAGGGAAATCTTATTTACACCAAATTGTATATATCTGTTTAATTAATAATTTATATATTATTAAAATGATGATGAATTTTACTGGTGATTTCATTATAAAGCAT
AATATTTGGAATTTTGATTAATTACACTCTATTACAAGTAAGAAATTTCCCAATTGGAACACGTTAACATCATGAATTTATTAATTTTTGGCAAGGCTTCTTAGTTCTCTCTCACCTATGGCATTATATA

62,864   TACTCGTGTATGTTACCACTGGACGCATATCTATCGATCATCCAAATATTTCTCTCTTTGCTTTGCTTTGTTTCTCTGAAAGCGAAAATGGCGTTTGTCACCGCCACAGCTGGTGTTCAAGTTCTGATCA   62,735
                                           Isoform X2 ATCGATCATCCAAATATTTCTCTCTTTGCTTTGCTTTGTTTCTCTGAAAGCGAAAATGGCGTTTGTCACCGCCACAGCTGGTGTTCAAGTTCTGATCA

62,734   AGAGTCAACCAGACACGTTCTGGACTAAAATTCAAGATTCCGCAAATTCGTTTCCTCAAGCCGCACCTAGCCTATATACATCAATTCTTATCAGCGGCACGGGGGTGCGCAAAGTCGAGTATGGCCCAGG   62,605
         AGAGTCAACCAGACACGTTCTGGACTAAAATTCAAGATTCCGCAAATTCGTTTCCTCAAGCCGCACCTAGCCTATATACATCAATTCTTATCAGCGGCACGGGGGTGCGCAAAGTCGAGTATGGCCCAG
```

```
62,604  CACGTACTTGCTCCAAGATAATTCTTTAGCACTTCTTTGGTCTTTTTATATATATACTTGAAAAAAATGTTAATGAGTAATTAATAAAGAATTAATGTTACTGAATTTGTTTTGTATGTAGATTCTCAGA  62,475
                                                                                                                                                  ATTCTCAGA
                                                                                                                                                  ATTCTCAGA

62,474  ATATCAAAACCTCGTCTGAGCTTATCACTGAAAACAGCTTGGGAAAATTAGCTTACACTGTAACTGCTGGTGATATCCTCCAAAAATATGATGTCAACAACTTCCAAGCTGTAATAATTCACCCAAATAG  62,345
        ATATCAAAACCTCGTCTGAGCTTATCACTGAAAACAGCTTGGGAAAATTAGCTTACACTGTAACTGCTGGTGATATCCTCCAAAAATATGATGTCAACAACTTCCAAGCTGTAATAATTCACCCAAATAG
        ATATCAAAACCTCGTCTGAGCTTATCACTGAAAACAGCTTGGGAAAATTAGCTTACACTGTAACTGCTGGTGATATCCTCCAAAAATATGATGTCAACAACTTCCAAGCTGTAATAATTCACCCAAATAG

62,344  TGAGAAATGGGTGGGATGGACCTGGACTTATAACTATCTCCCCGAGAATAAAGCAAGAGCTCTTCAGCTTGATGCAGAGATTGCAGTGATTGCAGTGAAGACCTTGACAGAACTTGATAACTATATTCAA  62,215
        TGAGAAATGGGTGGGATGGACCTGGACTTATAACTATCTCCCCGAGAATAAAGCAAGAGCTCTTCAGCTTGATGCAGAGATTGCAGTGATTGCAGTGAAGACCTTGACAGAACTTGATAACTATATTCAA
        TGAGAAATGGGTGGGATGGACCTGGACTTATAACTATCTCCCCGAGAATAAAGCAAGAGCTCTTCAGCTTGATGCAGAGATTGCAGTGATTGCAGTGAAGACCTTGACAGAACTTGATAACTATATTCAA

62,214  AACAAGTCAACCTAAATTGCATGGATGACCTTAAAGCGCGCGCGCGCGCACATATATATATATATATATATAGTATGTAAATAAAACAGAGCCTTGCTCTGCACTCATCCATGCATGTCTGCTAGATTATTG  62,085
        AACAAGTCAACCTAAATTGCATGGATGACCTTAAAGCGCGCGCGCGCGCACATATATATATATATATATATAGTATGTAAATAAAACAGAGCCTTGCTCTGCACTCATCCATGCATGTCTGCTAGATTATTG
        AACAAGTCAACCTAAATTGCATGGATGACCTTAAAGCGCGCGCGCGCGCACATATATATATATATATATATAGTATGTAAATAAAACAGAGCCTTGCTCTGCACTCATCCATGCATGTCTGCTAGATTATTG

62,084  TGTCTGTGTGTGTACCTAACAATGTGCTACGTGTGGTGTAGTAATAAATAAAGCAGAGTCCTATGTATGCTTAATGCTCTGCACTTTATCCTATAATTTTCTGCGTTATGCTATCTACTTTCTCCATTAA  61,955
        TGTCTGTGTGTGTACCTAACAATGTGCTACGTGTGGTGTAGTAATAAATAAAGCAGAGTCCTATGTATGCTTAATGCTCTGCACTTTATCCTATAATTTTCTGCGTTATGCTATCTACTTTCTCCATTAA
        TGTCTGTGTGTGTACCTAACAATGTGCTACGTGTGGTGTAGTAATAAATAAAGCAGAGTCCTATGTATGCTTAATGCTCTGCACTTTATCCTATAATTTTCTGCGTTATGCTATCTACTTTCTCCATTAA

61,954  AATAAAATCCAGCTTTAGCTGGTTCATGTGGTATTTGCTTCGACCTTATGTTTCCCATTTCACGAACCCAAAGCAAAGGACTTAAAGCTTGTTTGCCAACTTGCTTTGCATAATTAATTCTATAAGGGCC
        AATAAAATCCAGCTTTAGCTGGTTCATGTGGTA
        AATAAAATCCAGCTTTAGCTGGTTCATGTGGTA

61,824  AATTGGTATACATTTTTTTTAATTCATAATAACTTAATTTATACTTAATTGAATGATGCACAAAACATCAACATAGTTTAATCAATAATATAAGGGACATTAAAAAAAAAAAAAAGCTATCATCAATTCGT
61,694  CAAAGCATAAATGCATGGTAATAATAAAAATTGTTAATAAGAAAATAATTAATTTGAAAGAATGATTTTGAATAATAAATAAGCTACACATTAGGTTAGCTTTCTAGTAATTTTTTTTCTATCAAAGATA
        + 61,564 bp in the WGS contig
```

Supplementary Figure 5:

Location of the coding sequence of the uncharacterized protein LOC110748447 isoforms on whole genome shotgun contig MKXE01009552.1 (Range: 38570-39259)

Hevea brasiliensis cultivar RRIM600, whole genome shotgun sequence (matched 100% +/- with isoform X1)

The 3000 bp of 5’upstream region was the blue region

```
(reversed seq)
TTTTCTTGTCTCAACAAGCCTTTGTTGAGAAAGTGCTTAAGCGTTTTAACATGAATAATGCTAAGCCTGTGACTGTTCTGTTTGCTGCCCATTTCAAGTT    43,023
ATCTGCAGATATGTCACCAAAAATAGATGAGGAGATAGAGCACATGTCTAGTGTTCCCTATTCGAGTGCTGTTGGTAGCATCATGTATGCTATGGTATGC
ACACGACCTGACATTTTACATACAGTTAGTGTTGTGAGTAGATACATGGCGTGTCCTGGAAAAAAGTATTGGCAGGCAGTGAAATGGATTTTGAGGTATT
TGAAGGGTAATACAGATGTTGGTCTGACATTTGACAGGGCCAAGATGAGTGATTCAGTTGTTGGCTGTGTGGATTCAGATTTTGCAGGGGACTTAAACAA
GAGGAGATCTTTGAAAGGTTATTTGTTTACTCTTTATGGAAGTGCTATCAGTTGGAAGGCAACATTGCAAGCTACAGTTGTTTTGTCTACCACAGAGGCT
GAATATATGGCTTTAGCAGAGGCAGTAAAGGAAGCTTTATGGTAACAAGGTTTGGTGGGTGATCTTGGGTTGATACAGAATAAGACAACGGTGTTTTGTG
ACAGCCAGAGTACAATACATCTCATAAAGAATTAGATGTACCATGAGCGAACTAAGCACATTGATGTCAGATATCACTTCATTCAGGACATTGTATCTTA
GGAGACTGTAGTTGTGCAGAAAGTCTCTACACATGATAATCTAGTAGATATGATGACCAAGGGAGTCCTAGTCAGCAAGTTTAGGTATTACCTAGACTTG
GTTGGTGTTTGTAGTGCTCAGTAATGCCCTTGCGAGGGCATATGGCAAGACAGAGTGTTTTGTGGTTATTTTGTTGATGATTGAGAGAATGCAAGCCAAG
GGAATGAATTGATATTTTTGTGGCTTGAATTTTGGATATGTTTTTCATGGACTAGAATTGTGACCCAACTTGAAAGTTTCGCGCAGCGACCCGATTAGGA
TAAAATATTAGAGTTGTGAGATGTTCGGAAAACACATTGGGGTTAGGGTTTGAAAGTTTTAGTTATTGTATCTTACTCTTTTCATCATAGTAGAACTTT    42,023
TTCTCTTATCTTTCCAGTGGATGTAGACCTTACGGTTGAACCACGTTAAATTCTGTGTTATTCTACTTCTCTTTTTATACTGTGTGATTGTATGATTTGT
GTGTGTGCCTTGAATTGGCGTGCTGGTCATAACATTAAGTATATTATATAGTATAATAGAACACTAATAATTAAGTATTATAAAGTGATCTAATATATTT
GTAACTAAAATCATTAAAAAAAATAGAAGAATGATAGATAATATTAAGTTATATAAGCCATAATCATATAATATATATTATAATATATATATATATATAT
ATATATATATATATATATTATATATATATATAACCATCAAAATATATAATATATTTATAAAATGATTAAAACAACATATGCACGATTCATTTTTGATTTG
ATATAGGTCTAGCATTGATGTACCGTTTCAATTCAATTTCAGTGAAGAATTAAACTGGACAAAAAAAAATCGGTTCAATACAATTTTTTGATTTGGTTTG
AAAAGTGGTGGAGCTATAATTTCTAATGCGAGCAAAAGTTTTACTTCTTGTATTATAAAATATTTAAAAAAAAATATTTTTTTACTTACAAAATATTGACT
CATAAGCATATGAATAAATATACTTTTGAGAAAATTAATTATGTTAAAATTTCAATTTATTAAAACTCTGTCAATGTGCAAGATTTAATTATATCATTCA
CTATATCCTCACAAAAAATTGAAAAATAAATAATTATATATTTATTTTATATGTTGTCTTTAGTTTTGTTTTTTTAATCAATATGTTGTCTTTAGTTTAG
TATTACCAATCAATTAAAGTAATATAGTGGAATAAATATCAACTTTTTTTCACTTTAGCTTATACATTATAATTGAACAATTTTTAAATGTAATAATCTC
ATTGGAAGATTTTCAGATTATTTTATTATAAATAATTAAATAATTATAAGACTATTAATATATTTCATATCAAATTTTAGTTCTCTCATATATAAATTTT    41,023
ATATAGACTAGGAGAAAAAAAAAAATTCAGGCATCAAAATATATAGAATCATGATGGAAACTAATAAAACAAAAAATTTCAGTGAGCCAATGCCCTATAG
GTGAGCTGTAGCAAAAAATGAAAAAAATTAAAATATGAAAGTGGTCATATTTGAATTTCTGAATATTAATAAGTTAACTTATTATTTAAAGTGATGATAT
ATTATTTAATGTTTTAATTTTAGTTGAAAATATGAAAATTTAATTATAAATTTTTAGTTTTCAATTAATTTATTTACTTAATTTTATAATTATCAACTAA
GCTAATTTATTTGTTGTTTAAAAAGAATTGCATAAATCCATTTCAAGTTTACATTGTGAATAGATGAAATTCTGTCATCCTAAAAATGTCGCTATTATTA
TTATTATTAATTATTTATATTATTATTATTATTATTATTATATTATTATTATTATATTATTATTAATCTCTTGTTCATATTTCAAGAAGCAAACTGCAAT
CCTGTCCCATAAAAAAAAATAAATAACCCAACAAAAAGCTCTACAACAACGATAACCACGTTTAGAGTAATTAGTCCTCCTTTCACACTGTATGATACAC
ATTCGCTCAATTAACAAATGATATATTAATAAAATTACGATAAATTTTTTTATATAGCATGTGTTTATGATTTTTTAATAATTGGATATATAAACACATT
GGTGTAGTAAGAATTTTCATTCATATCCAAATTTTATTTCAAATAGATATAGATATTTAAAAATTATAATGAGGTTCAACAAAATAAACACTCTACAATA
AAAAGATACATATCCTTAAAAGTAGTCAAAATAGCAATAAAACTCCTCTAAAGGGAAACATAGCACTACATATATAATCAGAGGAAGTAATTAGAACTTT
AGCTTTTCACTTAATTCATTTATTTTTATATTTATCATCTAACCAAAATTTATTTATTTATTGCTTAAAAGGAATTGCATAATTTTAAATTTTGGCAATT    40,023
TCAGATATTGTACCTTACAAATCATTTCAAGTTTCCATAGTGAACAGATGGAATCACATCATCCTTATGATGTCACTGCGTATTTTTTTCTCCTTATTCA
TATTTCCAGAGAAATGAATGAATTACTATGCAAACCTAAATGAATGGCTTGAAAATGATAAATAAGATGTATATATGCACACACTAATTTTTATATCAAT
TAAATTTTATTTAATTATTTATTATGATAAGATTTAATTTTTATATCAATTAAATTTTATTTAATTATTTATTATGATAAGATTTGCTTTATCTACACAT
ATAGATATCATATAATCTTTATTTTGAATATGTGATCCAATAGCTCATTTTAATGTGGAAAATAATTTTTTGAACAAAAAAAAAAACTTATATTTATTCA
TTGAGCAATAAATTTGACGTCAACTTGAGGCAAAGTTAGGAGTTTCTTATAATTAAAAAAACTTATATATGTTTAAAATTTTCAAATTATCAGAAGGGAA
ATCTTATTTACACCAAATTGTATATATCTGTTTAATTAATAATTTATATATTATTAAAATGATGATGAATTTTACTGGTGATTTCATTATAAAGCATAAT
ATTTGGAATTTTGATTAATTACACTCTATTACAAGTAAGAAATTTCCCAATTGGAATACGTTAACATCATGAATTTATTAATTTTTGGCAGGGCTTCTTG
```

```
GTTCTCTCTCACCTATGGCATTATATATACTTGTGTATGTTGCCTCTGGACGCATATCTATCGATCATCCAAATAATTCTCTCTTCATTTGCTTTGCTTT    39,223
                                                           isoform X1 ATATCTATCGATCATCCAAATAATTCTCTCTTCATTTGCTTTGCTTT
                                                                          isoform X3 TCCAAATAATTCTCTCTTCATTTGCTTTGCTTT
                                                                isoform X2 ATCGATCATCCAAATA-TT-TCTC-TC-TTTGCTTTGC---

GTTTTGTTTCTCTGTAAGCGAAAATGGCATTTGTCACCGCCACAGCTGGTGTTCAAGTTCTGATCAAGAGTCTACCAGACACGTTCTGGACTAAAATTCA    39,123
GTTTTGTTTCTCTGTAAGCGAAAATGGCATTTGTCACCGCCACAGCTGGTGTTCAAGTTCTGATCAAGAGTCTACCAGACACGTTCTGGACTAAAATTCA
GTTTTGTTTCTCTGTAAGCGAAAATGGCATTTGTCACCGCCACAGCTGGTGTTCAAGTTCTGATCAAGAGTCTACCAGACACGTTCTGGACTAAAATTCA
--TTTGTTTCTCTGAAAGCGAAAATGGCGTTTGTCACCGCCACAGCTGGTGTTCAAGTTCTGATCAAGAGTCAACCAGACACGTTCTGGACTAAAATTCA

AGATTCCGCAAATTCTTTTCCTGAAGCCGCACCAAACCTATATTCATCAATTGCTGTCAGCGGCACGGGGGTACGCAAAGTCGAGTATGGCCCAGGTACG    39,023
AGATTCCGCAAATTCTTTTCCTGAAGCCGCACCAAACCTATATTCATCAATTGCTGTCAGCGGCACGGGGGTACGCAAAGTCGAGTATGGCCCAG
AGATTCCGCAAATTCTTTTCCTGAAGCCGCACCAAACCTATATTCATCAATTGCTGTCAGCGGCACGGGGGTACGCAAAGTCGAGTATGGCCCAG
AGATTCCGCAAATTCGTTTCCTCAAGCCGCACCTAGCCTATATACATCAATTCTTATCAGCGGCACGGGGGTGCGCAAAGTCGAGTATGGCCCAG

TACTTGCTCCAAGATAATTCTTTAGCACTTCTTTGGTCTTTTTATATACATACTTGAAAAAAATGTTAATGAGTAAATTAATAAAGAATTAATGTTAGTG    38,923

AATTTGTTTTGTATGTAGATTCTCAGAATATCAAAAACTCGTCTGAGCTTATCACTGAAAACAGTCGGGGAAAATTAGCTTACACTGTAACGGATGGTGA    38,823
                  ATTCTCAGAATATCAAAAACTCGTCTGAGCTTATCACTGAAAACAGTCGGGGAAAATTAGCTTACACTGTAACGGATGGTGA
                  ATTCTCAGAATATCAAAACCTCGTCTGAGCTTATCACTGAAAACAGCTTGGGAAAATTAGCTTACACTGTAACTGCTGGTGA
                  ATTCTCAGAATATCAAAACCTCGTCTGAGCTTATCACTGAAAACAGCTTGGGAAAATTAGCTTACACTGTAACTGCTGGTGA

TATCCTCCAAAAATATAAAGTCAAAACCTTCCAGGCTGAAATAATTTACCCAAATACTGGCAAATGGGTGGAATGGATCTGGACCTATAACTATCTCCCA    38,723
TATCCTCCAAAAATATAAAGTCAAAACCTTCCAGGCTGAAATAATTTACCCAAATACTGGCAAATGGGTGGAATGGATCTGGACCTATAACTATCTCCCA
TATCCTCCAAAAATATGATGTCAACAACTTCCAAGCTGTAATAATTCACCCAAATAGTGAGAAATGGGTGGGATGGACCTGGACTTATAACTATCTCCCC
TATCCTCCAAAAATATGATGTCAACAACTTCCAAGCTGTAATAATTCACCCAAATAGTGAGAAATGGGTGGGATGGACCTGGACTTATAACTATCTCCCC

GAGGATAAAACAAGGATTCTTGAACTTGATGAAGAGATTGCAGTGATTACAGTGAAGACCCTGGCAAAACTTGATAGCTATATTCAAAACAAGTTAACCT    38,623
GAGGATAAAACAAGGATTCTTGAACTTGATGAAGAGATTGCAGTGATTACAGTGAAGACCCTGGCAAAACTTGATAGCTATATTCAAAACAAGTTAACCT
GAGAATAAAGCAAGAGCTCTTCAGCTTGATGCAGAGATTGCAGTGATTGCAGTGAAGACCTTGACAGAACTTGATAACTATATTCAAAACAAGTCAACCT
GAGAATAAAGCAAGAGCTCTTCAGCTTGATGCAGAGATTGCAGTGATTGCAGTGAAGACCTTGACAGAACTTGATAACTATATTCAAAACAAGTCAACCT

AAATTGCATGGATGACCTTCAAGCA------------CATATATATGTATAGTAT------GTAAATAAAACAGAGCCTTGCTCTGCACTCATCCATGCATGTCTGCTAGATTATTGT   38,523
AAATTGCATGGATGACCTTCAAGCA------------CATATATATGTATAGTAT
AAATTGCATGGATGACCTTAAAGCGCGCGCGCGCGCACATATATATATATATATATAGTATGTAAATAAAACAGAGCCTTGCTCTGCACTCATCCATGCATGTCTGCTAGATTATTGT
AAATTGCATGGATGACCTTAAAGCGCGCGCGCGCGCACATATATATATATATATATAGTATGTAAATAAAACAGAGCCTTGCTCTGCACTCATCCATGCATGTCTGCTAGATTATTGT

GTCTGTGTGTGTACCTAACAATGTGCTACGTGTGGTGTAGTAATAAATAAAGCAGAGTCCTATGTATGCTTAATGCTCTGCACTTTATCCTATAATTTTC    38,423
GTCTGTGTGTGTACCTAACAATGTGCTACGTGTGGTGTAGTAATAAATAAAGCAGAGTCCTATGTATGCTTAATGCTCTGCACTTTATCCTATAATTTTC
GTCTGTGTGTGTACCTAACAATGTGCTACGTGTGGTGTAGTAATAAATAAAGCAGAGTCCTATGTATGCTTAATGCTCTGCACTTTATCCTATAATTTTC

TGCGTTATGCTATCTACTTTCTCCATTAAAATAAAATCCAGCTTTAGCTGGTTCATGTGGTATTTGCTTCGACCTTATGTTTCCCATTTCACGAACCCAA    38,323
TGCGTTATGCTATCTACTTTCTCCATTAAAATAAAATCCAGCTTTAGCTGGTTCATGTGGTA
TGCGTTATGCTATCTACTTTCTCCATTAAAATAAAATCCAGCTTTAGCTGGTTCATGTGGTA

AGCAAAGGACTTAAAGCTTGTTTGCCAACTTGCTTTGCATAATTAATTCAATAAGGGCCAATTGTTATACATTTTTTTTTAATTCATAATAACTTAATTT    38,223
```

Supplementary Figure 6:

Location of predicted cis-acting elements on the promoter region of Uncharacterized protein LOC110648447

```
RESULTS OF SIGNAL SCAN SEARCH in NEWPLACE promoter database (https://www.dna.affrc.go.jp/PLACE/?action=newplace)
Using 3000 bp upstream sequence of uncharacterized protein LOC110648447 from rubber tree clone RRIM600
This result is the output of the new signal scan program which was completely rewritten from a scratch by Akio Miyao ($Id: 649.pl,v 1.11 2016/04/20 08:43:39 miyao
Exp $).

The original program of signal scan was reported in Prestridge, D.S. (1991) SIGNAL SCAN: A computer program that scans DNA sequences for eukaryotic transcriptional
elements. CABIOS 7, 203-206.

3000 base pairs

 (+) = Current Strand
 (-) = Opposite Strand

1      TTTCGCTTACAGAGAAACAAAACAAAGCAAAGCAAATGAAGAGAGAATTA
                   (+) POLLEN1LELAT52 S000245 13 AGAAA
                     (+) ANAERO1CONSENSUS S000477 15 AAACAAA
                          (+) ANAERO1CONSENSUS S000477 20 AAACAAA
                              (+) DOFCOREZM S000265 24 AAAG
                                   (+) DOFCOREZM S000265 29 AAAG
                                       (-) EBOXBNNAPA S000144 33 CANNTG
                                       (-) MYCCONSENSUSAT S000407 33 CANNTG
                                       (+) EBOXBNNAPA S000144 33 CANNTG
                                       (+) MYCCONSENSUSAT S000407 33 CANNTG
                                             (-) NODCON2GM S000462 39 CTCTT
                                             (-) OSE2ROOTNODULE S000468 39 CTCTT
                                                     (-) POLASIG3 S000088 47 AATAAT
                                                      (+) TATABOX5 S000203 48 TTATTT

51     TTTGGATGATCGATAGATATGCGTCCAGAGGCAACATACACAAGTATATA
                  (+) GATABOX S000039 62 GATA
                      (+) GATABOX S000039 66 GATA
                                      (+) RAV1AAT S000314 82 CAACA
                                            (+) DPBFCOREDCDC3 S000292 88 ACACNNG
                                                 (-) CACTFTPPCA1 S000449 93 YACT
                                                     (+) TATABOX4 S000111 97 TATATAA

101    TAATGCCATAGGTGAGAGAGAACCAAGAAGCCCTGCCAAAAATTAATAAA
                  (+) GTGANTG10 S000378 112 GTGA
                           (+) REALPHALGLHCB21 S000362 121 AACCAA
                                           (-) SEF4MOTIFGM7S S000103 137 RTTTTTR
                                                (-) TATABOX3 S000110 142 TATTAAT
                                                   (+) POLASIG1 S000080 145 AATAAA

151    TTCATGATGTTAACGTATTCCAATTGGGAAATTTCTTACTTGTAATAGAG
                 (+) GARE2OSREP1 S000420 161 TAACGTA
```

(-) ACGTATERD1 S000415 163 ACGT
(+) ACGTATERD1 S000415 163 ACGT
(+) CCAATBOX1 S000030 170 CCAAT
(-) EBOXBNNAPA S000144 171 CANNTG
(-) MYCCONSENSUSAT S000407 171 CANNTG
(+) CAATBOX1 S000028 171 CAAT
(+) EBOXBNNAPA S000144 171 CANNTG
(+) MYCCONSENSUSAT S000407 171 CANNTG
(-) CAATBOX1 S000028 173 CAAT
(-) CCAATBOX1 S000030 173 CCAAT
(+) GT1CONSENSUS S000198 177 GRWAAW
(-) POLLEN1LELAT52 S000245 182 AGAAA
(+) CACTFTPPCA1 S000449 187 YACT
(-) CACTFTPPCA1 S000449 199 YACT

201 TGTAATTAATCAAAATTCCAAATATTATGCTTTATAATGAAATCACCAGT
(-) ARR1AT S000454 208 NGATT
(-) ROOTMOTIFTAPOX1 S000098 221 ATATT
(+) ROOTMOTIFTAPOX1 S000098 222 ATATT
(-) DOFCOREZM S000265 230 AAAG
(-) TAAAGSTKST1 S000387 230 TAAAG
(-) CARGCW8GAT S000431 230 CWWWWWWWWG
(+) CARGCW8GAT S000431 230 CWWWWWWWWG
(-) ARR1AT S000454 241 NGATT
(-) GTGANTG10 S000378 243 GTGA
(-) CACTFTPPCA1 S000449 248 YACT

251 AAAATTCATCATCATTTTAATAATATATAAATTATTAATTAAACAGATAT
(+) POLASIG3 S000088 269 AATAAT
(-) ROOTMOTIFTAPOX1 S000098 272 ATATT
(-) TATAPVTRNALEU S000340 274 TTTATATA
(+) TATABOX4 S000111 274 TATATAA
(+) TATABOX2 S000109 276 TATAAAT
(-) POLASIG3 S000088 281 AATAAT
(+) TATABOX3 S000110 283 TATTAAT
(+) POLASIG2 S000081 287 AATTAAA
(+) GATABOX S000039 296 GATA
(-) SORLREP3AT S000488 297 TGTATATAT

301 ATACAATTTGGTGTAAATAAGATTTCCCTTCTGATAATTTGAAAATTTTA
(+) CAATBOX1 S000028 304 CAAT
(-) TATABOX5 S000203 315 TTATTT
(+) ARR1AT S000454 320 NGATT
(+) EECCRCAH1 S000494 321 GANTTNC
(-) GT1CONSENSUS S000198 322 GRWAAW
(+) GATABOX S000039 333 GATA
(+) GT1CONSENSUS S000198 333 GRWAAW
(+) IBOXCORE S000199 333 GATAA
(+) GT1CONSENSUS S000198 341 GRWAAW

351 AACATATATAAGTTTTTTTAATTATAAGAAACTCCTAACTTTGCCTCAAG
(-) CARGCW8GAT S000431 353 CWWWWWWWWG
(+) CARGCW8GAT S000431 353 CWWWWWWWWG
(+) TATABOX4 S000111 355 TATATAA
(-) POLASIG2 S000081 367 AATTAAA
(+) POLLEN1LELAT52 S000245 377 AGAAA
(-) EECCRCAH1 S000494 378 GANTTNC
(+) TBOXATGAPB S000383 388 ACTTTG
(-) DOFCOREZM S000265 389 AAAG

401 TTGACGTCAAATTTATTGCTCAATGAATAAATATAAGTTTTTTTTTTTGT
(+) WBOXATNPR1 S000390 401 TTGAC
(-) HEXMOTIFTAH3H4 S000053 402 ACGTCA
(-) PALINDROMICCBOXGM S000255 402 TGACGTCA
(+) ASF1MOTIFCAMV S000024 402 TGACG
(+) PALINDROMICCBOXGM S000255 402 TGACGTCA
(+) TGACGTVMAMY S000377 402 TGACGT
(+) WRKY71OS S000447 402 TGAC
(-) ACGTCBOX S000131 403 GACGTC
(+) ACGTCBOX S000131 403 GACGTC
(-) TGACGTVMAMY S000377 404 TGACGT
(-) ACGTATERD1 S000415 404 ACGT
(+) HEXMOTIFTAH3H4 S000053 404 ACGTCA
(+) ACGTATERD1 S000415 404 ACGT
(-) ASF1MOTIFCAMV S000024 405 TGACG
(-) WBOXATNPR1 S000390 406 TTGAC
(-) WRKY71OS S000447 406 TGAC
(-) POLASIG1 S000080 412 AATAAA
(-) CAATBOX1 S000028 415 CAAT
(+) CAATBOX1 S000028 421 CAAT
(-) SEF1MOTIF S000006 426 ATATTTAWW
(+) POLASIG1 S000080 426 AATAAA
(-) ROOTMOTIFTAPOX1 S000098 430 ATATT
(+) MARTBOX S000067 438 TTWTWTTWTT
(+) MARTBOX S000067 439 TTWTWTTWTT

451 TCAAAAAATTATTTTCCACATTAAAATGAGCTATTGGATCACATATTCAA
(-) POLASIG3 S000088 458 AATAAT
(+) TATABOX5 S000203 459 TTATTT
(-) GT1CONSENSUS S000198 461 GRWAAW
(-) GT1CONSENSUS S000198 462 GRWAAW
(-) CARGCW8GAT S000431 469 CWWWWWWWWG
(+) CARGCW8GAT S000431 469 CWWWWWWWWG
(-) INRNTPSADB S000395 473 YTCANTYY
(-) CAATBOX1 S000028 483 CAAT
(-) CCAATBOX1 S000030 483 CCAAT
(-) GTGANTG10 S000378 489 GTGA
(+) ROOTMOTIFTAPOX1 S000098 493 ATATT

(-) CARGCW8GAT S000431 498 CWWWWWWWWG
(+) CARGCW8GAT S000431 498 CWWWWWWWWG
(-) TATABOX5 S000203 500 TTATTT

501 AATAAAGATTATATGATATCTATATGTGTAGATAAAGCAAATCTTATCAT
(+) POLASIG1 S000080 501 AATAAA
(+) TAAAGSTKST1 S000387 503 TAAAG
(+) DOFCOREZM S000265 504 AAAG
(+) NODCON1GM S000461 504 AAAGAT
(+) OSE1ROOTNODULE S000467 504 AAAGAT
(+) ARR1AT S000454 506 NGATT
(+) GATABOX S000039 515 GATA
(-) GATABOX S000039 517 GATA
(-) NAPINMOTIFBN S000070 524 TACACAT
(+) GATABOX S000039 531 GATA
(+) GT1CONSENSUS S000198 531 GRWAAW
(+) IBOXCORE S000199 531 GATAA
(+) TAAAGSTKST1 S000387 533 TAAAG
(+) DOFCOREZM S000265 534 AAAG
(-) EECCRCAH1 S000494 537 GANTTNC
(-) ARR1AT S000454 540 NGATT
(-) IBOXCORENT S000424 542 GATAAGR
(-) IBOX S000124 543 GATAAG
(-) IBOXCORE S000199 544 GATAA
(-) GATABOX S000039 545 GATA

551 AATAAATAATTAAATAAAATTTAATTGATATAAAAATTAAATCTTATCAT
(+) POLASIG1 S000080 551 AATAAA
(-) TATABOX5 S000203 554 TTATTT
(+) POLASIG3 S000088 555 AATAAT
(+) POLASIG2 S000081 558 AATTAAA
(-) TATABOXOSPAL S000400 560 TATTTAA
(-) TATABOX5 S000203 562 TTATTT
(+) POLASIG1 S000080 563 AATAAA
(-) POLASIG2 S000081 570 AATTAAA
(-) CAATBOX1 S000028 574 CAAT
(+) GATABOX S000039 577 GATA
(-) SEF4MOTIFGM7S S000103 581 RTTTTTR
(+) POLASIG2 S000081 585 AATTAAA
(-) ARR1AT S000454 590 NGATT
(-) IBOXCORENT S000424 592 GATAAGR
(-) IBOX S000124 593 GATAAG
(-) IBOXCORE S000199 594 GATAA
(-) GATABOX S000039 595 GATA

601 AATAAATAATTAAATAAAATTTAATTGATATAAAAATTAGTGTGTGCATA
(+) POLASIG1 S000080 601 AATAAA
(-) TATABOX5 S000203 604 TTATTT
(+) POLASIG3 S000088 605 AATAAT

```
         (+) POLASIG2 S000081 608 AATTAAA
           (-) TATABOXOSPAL S000400 610 TATTTAA
             (-) TATABOX5 S000203 612 TTATTT
              (+) POLASIG1 S000080 613 AATAAA
                     (-) POLASIG2 S000081 620 AATTAAA
                         (-) CAATBOX1 S000028 624 CAAT
                            (+) GATABOX S000039 627 GATA
                                (-) SEF4MOTIFGM7S S000103 631 RTTTTTR
                                        (-) CACTFTPPCA1 S000449 639 YACT
                                                 (-) SORLREP3AT S000488 648 TGTATATAT

651    TATACATCTTATTTATCATTTTCAAGCCATTCATTTAGGTTTGCATAGTA
        (+) TATABOX5 S000203 659 TTATTT
           (-) GT1CONSENSUS S000198 662 GRWAAW
            (-) IBOXCORE S000199 663 GATAA
             (-) GATABOX S000039 664 GATA
                 (-) GT1CONSENSUS S000198 668 GRWAAW
                                                (-) CACTFTPPCA1 S000449 697 YACT
                                                 (-) EECCRCAH1 S000494 698 GANTTNC

701    ATTCATTCATTTCTCTGGAAATATGAATAAGGAGAAAAAAATACGCAGTG
     (+) INRNTPSADB S000395 706 YTCANTYY
         (-) POLLEN1LELAT52 S000245 710 AGAAA
                (+) GT1CONSENSUS S000198 717 GRWAAW
                   (-) ROOTMOTIFTAPOX1 S000098 720 ATATT
                                (+) POLLEN1LELAT52 S000245 733 AGAAA
                                 (+) GT1CONSENSUS S000198 734 GRWAAW
                                 (+) GT1GMSCAM4 S000453 734 GAAAAA
                                                (-) CACTFTPPCA1 S000449 747 YACT
                                                 (+) GTGANTG10 S000378 748 GTGA
                                                  (-) BIHD1OS S000498 749 TGTCA
                                                  (+) WRKY71OS S000447 749 TGAC

751    ACATCATAAGGATGATGTGATTCCATCTGTTCACTATGGAAACTTGAAAT
                (+) GTGANTG10 S000378 767 GTGA
                 (+) ARR1AT S000454 768 NGATT
                       (-) EBOXBNNAPA S000144 774 CANNTG
                       (-) MYCCONSENSUSAT S000407 774 CANNTG
                       (+) EBOXBNNAPA S000144 774 CANNTG
                       (+) MYCCONSENSUSAT S000407 774 CANNTG
                              (-) GTGANTG10 S000378 781 GTGA
                               (+) CACTFTPPCA1 S000449 782 YACT
                                                   (+) ARR1AT S000454 800 NGATT

801    GATTTGTAAGGTACAATATCTGAAATTGCCAAAATTTAAAATTATGCAAT
          (-) CURECORECR S000493 811 GTAC
          (+) CURECORECR S000493 811 GTAC
             (+) CAATBOX1 S000028 814 CAAT
              (-) ROOTMOTIFTAPOX1 S000098 815 ATATT
```

(-) GATABOX S000039 817 GATA
(-) CAATBOX1 S000028 825 CAAT
(-) EECCRCAH1 S000494 846 GANTTNC
(+) CAATBOX1 S000028 847 CAAT

851 TCCTTTTAAGCAATAAATAAATAAATTTTGGTTAGATGATAAATATAAAA
(+) PYRIMIDINEBOXOSRAMY1A S000259 852 CCTTTT
(-) DOFCOREZM S000265 853 AAAG
(+) CAATBOX1 S000028 861 CAAT
(+) MARABOX1 S000063 862 AATAAAYAAA
(+) POLASIG1 S000080 862 AATAAA
(-) TATABOX5 S000203 865 TTATTT
(+) MARABOX1 S000063 866 AATAAAYAAA
(+) POLASIG1 S000080 866 AATAAA
(-) TATABOX5 S000203 869 TTATTT
(+) POLASIG1 S000080 870 AATAAA
(-) REALPHALGLHCB21 S000362 878 AACCAA
(-) MYBATRD22 S000175 879 CTAACCA
(-) MYB1AT S000408 879 WAACCA
(+) GATABOX S000039 888 GATA
(+) GT1CONSENSUS S000198 888 GRWAAW
(+) IBOXCORE S000199 888 GATAA
(-) MARARS S000064 890 WTTTATRTTTW
(-) ROOTMOTIFTAPOX1 S000098 892 ATATT
(-) SEF4MOTIFGM7S S000103 896 RTTTTTR
(-) TATABOX5 S000203 899 TTATTT
(+) POLASIG1 S000080 900 AATAAA

901 ATAAATGAATTAAGTGAAAAGCTAAAGTTCTAATTACTTCCTCTGATTAT
(-) CACTFTPPCA1 S000449 913 YACT
(+) GTGANTG10 S000378 914 GTGA
(+) DOFCOREZM S000265 918 AAAG
(-) NTBBF1ARROLB S000273 923 ACTTTA
(+) TAAAGSTKST1 S000387 923 TAAAG
(+) DOFCOREZM S000265 924 AAAG
(+) CACTFTPPCA1 S000449 935 YACT
(+) ARR1AT S000454 944 NGATT
(-) TATABOX4 S000111 947 TATATAA

951 ATATGTAGTGCTATGTTTCCCTTTAGAGGAGTTTTATTGCTATTTTGACT
(-) CACTFTPPCA1 S000449 957 YACT
(-) DOFCOREZM S000265 971 AAAG
(-) TAAAGSTKST1 S000387 971 TAAAG
(-) POLASIG1 S000080 983 AATAAA
(-) CAATBOX1 S000028 986 CAAT
(+) WBBOXPCWRKY1 S000310 994 TTTGACY
(+) WBOXATNPR1 S000390 995 TTGAC
(+) WBOXHVISO1 S000442 996 TGACT
(+) WRKY71OS S000447 996 TGAC

(+) WBOXNTERF3 S000457 996 TGACY
(+) CACTFTPPCA1 S000449 1000 YACT

1001 ACTTTTAAGGATATGTATCTTTTTATTGTAGAGTGTTTATTTTGTTGAAC
(-) DOFCOREZM S000265 1002 AAAG
(+) MYBST1 S000180 1009 GGATA
(+) GATABOX S000039 1010 GATA
(-) GATABOX S000039 1016 GATA
(-) NODCON1GM S000461 1017 AAAGAT
(-) OSE1ROOTNODULE S000467 1017 AAAGAT
(-) DOFCOREZM S000265 1019 AAAG
(-) CARGCW8GAT S000431 1019 CWWWWWWWWG
(+) CARGCW8GAT S000431 1019 CWWWWWWWWG
(-) POLASIG1 S000080 1022 AATAAA
(-) CAATBOX1 S000028 1025 CAAT
(-) CACTFTPPCA1 S000449 1032 YACT
(-) POLASIG1 S000080 1036 AATAAA
(+) TATABOX5 S000203 1037 TTATTT
(-) RAV1AAT S000314 1043 CAACA

1051 CTCATTATAATTTTTAAATATCTATATCTATTTGAAATAAAATTTGGATA
(+) SEF4MOTIFGM7S S000103 1060 RTTTTTR
(-) SEF1MOTIF S000006 1063 ATATTTAWW
(-) TATABOXOSPAL S000400 1064 TATTTAA
(-) ROOTMOTIFTAPOX1 S000098 1067 ATATT
(-) GATABOX S000039 1069 GATA
(-) GATABOX S000039 1075 GATA
(-) ERELEE4 S000037 1081 AWTTCAAA
(-) TATABOX5 S000203 1085 TTATTT
(+) POLASIG1 S000080 1086 AATAAA
(-) TATCCAOSAMY S000403 1095 TATCCA
(+) MYBST1 S000180 1096 GGATA
(+) GATABOX S000039 1097 GATA

1101 TGAATGAAAATTCTTACTACACCAATGTGTTTATATATCCAATTATTAAA
(+) GT1CONSENSUS S000198 1106 GRWAAW
(+) CACTFTPPCA1 S000449 1115 YACT
(+) CCAATBOX1 S000030 1122 CCAAT
(+) LEAFYATAG S000432 1122 CCAATGT
(+) CAATBOX1 S000028 1123 CAAT
(+) TATAPVTRNALEU S000340 1130 TTTATATA
(-) TATABOX4 S000111 1131 TATATAA
(-) GATABOX S000039 1136 GATA
(-) MYBST1 S000180 1136 GGATA
(+) TATCCAOSAMY S000403 1136 TATCCA
(+) CCAATBOX1 S000030 1139 CCAAT
(+) CAATBOX1 S000028 1140 CAAT
(+) ATHB6COREAT S000399 1140 CAATTATTA
(-) POLASIG3 S000088 1142 AATAAT

```
1151   AAATCATAAACACATGCTATATAAAAAAATTTATCGTAATTTTATTAATA
        (-) ARR1AT S000454 1152 NGATT
                (+) DPBFCOREDCDC3 S000292 1160 ACACNNG
                 (-) EBOXBNNAPA S000144 1161 CANNTG
                 (-) MYCCONSENSUSAT S000407 1161 CANNTG
                 (-) MYCATERD1 S000413 1161 CATGTG
                 (+) EBOXBNNAPA S000144 1161 CANNTG
                 (+) MYCATRD22 S000174 1161 CACATG
                 (+) MYCCONSENSUSAT S000407 1161 CANNTG
                        (-) TATAPVTRNALEU S000340 1168 TTTATATA
                        (+) TATABOX4 S000111 1168 TATATAA
                                    (-) GT1CONSENSUS S000198 1180 GRWAAW
                                     (-) IBOXCORE S000199 1181 GATAA
                                      (-) GATABOX S000039 1182 GATA
                                               (-) POLASIG1 S000080 1191 AATAAA
                                                 (+) TATABOX3 S000110 1193 TATTAAT
                                                  (-) TATABOX3 S000110 1194 TATTAAT
                                                     (-) ROOTMOTIFTAPOX1 S000098 1197 ATATT

1201   TATCATTTGTTAATTGAGCGAATGTGTATCATACAGTGTGAAAGGAGGAC
       (-) GATABOX S000039 1201 GATA
          (-) EBOXBNNAPA S000144 1204 CANNTG
          (-) MYCCONSENSUSAT S000407 1204 CANNTG
          (+) EBOXBNNAPA S000144 1204 CANNTG
          (+) MYCCONSENSUSAT S000407 1204 CANNTG
            (-) AMYBOX1 S000020 1206 TAACARA
            (-) MYBGAHV S000181 1206 TAACAAA
            (-) GAREAT S000439 1206 TAACAAR
                   (-) CAATBOX1 S000028 1213 CAAT
                            (-) NAPINMOTIFBN S000070 1222 TACACAT
                                 (-) GATABOX S000039 1227 GATA
                                         (-) CACTFTPPCA1 S000449 1235 YACT
                                            (+) GTGANTG10 S000378 1238 GTGA
                                               (+) DOFCOREZM S000265 1241 AAAG

1251   TAATTACTCTAAACGTGGTTATCGTTGTTGTAGAGCTTTTTGTTGGGTTA
           (+) CACTFTPPCA1 S000449 1255 YACT
                  (+) T/GBOXATPIN2 S000458 1262 AACGTG
                  (+) ABRERATCAL S000507 1262 MACGYGB
                   (-) ACGTATERD1 S000415 1263 ACGT
                   (+) ABRELATERD1 S000414 1263 ACGTG
                   (+) ACGTATERD1 S000415 1263 ACGT
                      (-) MYB1AT S000408 1266 WAACCA
                         (-) IBOXCORE S000199 1269 GATAA
                          (-) GATABOX S000039 1270 GATA
                                (-) RAV1AAT S000314 1276 CAACA
                                          (-) DOFCOREZM S000265 1286 AAAG
                                               (-) RAV1AAT S000314 1291 CAACA
```

(-) SEF3MOTIFGM S000115 1294 AACCCA
(+) TATABOX5 S000203 1298 TTATTT

1301 TTTATTTTTTTTTATGGGACAGGATTGCAGTTTGCTTCTTGAAATATGAA
(-) POLASIG1 S000080 1301 AATAAA
(+) MARTBOX S000067 1302 TTWTWTTWTT
(+) TATABOX5 S000203 1302 TTATTT
(-) AUXREPSIAA4 S000026 1314 KGTCCCAT
(+) ARR1AT S000454 1322 NGATT
(-) CAATBOX1 S000028 1324 CAAT
(-) ROOTMOTIFTAPOX1 S000098 1343 ATATT

1351 CAAGAGATTAATAATAATATAATAATAATAATATAATAATAATAATAATA
(-) NODCON2GM S000462 1352 CTCTT
(-) OSE2ROOTNODULE S000468 1352 CTCTT
(+) ARR1AT S000454 1355 NGATT
(-) TATABOX3 S000110 1357 TATTAAT
(+) POLASIG3 S000088 1360 AATAAT
(-) MARTBOX S000067 1363 TTWTWTTWTT
(+) POLASIG3 S000088 1363 AATAAT
(-) ROOTMOTIFTAPOX1 S000098 1366 ATATT
(+) POLASIG3 S000088 1371 AATAAT
(+) POLASIG3 S000088 1374 AATAAT
(-) MARTBOX S000067 1377 TTWTWTTWTT
(+) POLASIG3 S000088 1377 AATAAT
(-) ROOTMOTIFTAPOX1 S000098 1380 ATATT
(+) POLASIG3 S000088 1385 AATAAT
(+) POLASIG3 S000088 1388 AATAAT
(+) POLASIG3 S000088 1391 AATAAT
(+) POLASIG3 S000088 1394 AATAAT
(+) POLASIG3 S000088 1397 AATAAT
(-) MARTBOX S000067 1400 TTWTWTTWTT
(+) POLASIG3 S000088 1400 AATAAT

1401 ATAATATAAATAATTAATAATAATAATAATAGCGACATTTTTAGGATGAC
(-) ROOTMOTIFTAPOX1 S000098 1403 ATATT
(+) TATABOX2 S000109 1405 TATAAAT
(-) TATABOX5 S000203 1408 TTATTT
(+) POLASIG3 S000088 1409 AATAAT
(-) TATABOX3 S000110 1413 TATTAAT
(+) POLASIG3 S000088 1416 AATAAT
(+) POLASIG3 S000088 1419 AATAAT
(+) POLASIG3 S000088 1422 AATAAT
(+) POLASIG3 S000088 1425 AATAAT
(+) SEF4MOTIFGM7S S000103 1437 RTTTTTR
(-) BIHD1OS S000498 1447 TGTCA
(+) WRKY71OS S000447 1447 TGAC

1451 AGAATTTCATCTATTCACAATGTAAACTTGAAATGGATTTATGCAATTCT

(+) EECCRCAH1 S000494 1452 GANTTNC
(-) GTGANTG10 S000378 1465 GTGA
(+) CAATBOX1 S000028 1468 CAAT
(+) ARR1AT S000454 1485 NGATT
(-) EECCRCAH1 S000494 1493 GANTTNC
(+) CAATBOX1 S000028 1494 CAAT
(-) DOFCOREZM S000265 1499 AAAG

1501 TTTTAAACAACAAATAAATTAGCTTAGTTGATAATTATAAAATTAAGTAA
(+) RAV1AAT S000314 1508 CAACA
(-) TATABOX5 S000203 1512 TTATTT
(+) POLASIG1 S000080 1513 AATAAA
(+) GATABOX S000039 1530 GATA
(+) GT1CONSENSUS S000198 1530 GRWAAW
(+) IBOXCORE S000199 1530 GATAA
(-) CACTFTPPCA1 S000449 1546 YACT
(-) TATABOX5 S000203 1549 TTATTT
(+) POLASIG1 S000080 1550 AATAAA

1551 ATAAATTAATTGAAAACTAAAAATTTATAATTAAATTTTCATATTTTCAA
(-) CAATBOX1 S000028 1559 CAAT
(+) CEREGLUBOX2PSLEGA S000033 1561 TGAAAACT
(-) SEF4MOTIFGM7S S000103 1568 RTTTTTR
(-) TATABOX2 S000109 1573 TATAAAT
(+) POLASIG2 S000081 1579 AATTAAA
(-) GT1CONSENSUS S000198 1585 GRWAAW
(+) ROOTMOTIFTAPOX1 S000098 1591 ATATT
(-) GT1CONSENSUS S000198 1593 GRWAAW

1601 CTAAAATTAAAACATTAAATAATATATCATCACTTTAAATAATAAGTTAA
(+) POLASIG2 S000081 1605 AATTAAA
(-) TATABOXOSPAL S000400 1615 TATTTAA
(-) TATABOX5 S000203 1617 TTATTT
(+) POLASIG3 S000088 1618 AATAAT
(-) ROOTMOTIFTAPOX1 S000098 1621 ATATT
(-) GATABOX S000039 1625 GATA
(+) ANAERO3CONSENSUS S000479 1627 TCATCAC
(-) GTGANTG10 S000378 1630 GTGA
(+) CACTFTPPCA1 S000449 1631 YACT
(+) NTBBF1ARROLB S000273 1632 ACTTTA
(-) DOFCOREZM S000265 1633 AAAG
(-) TAAAGSTKST1 S000387 1633 TAAAG
(-) TATABOXOSPAL S000400 1635 TATTTAA
(-) TATABOX5 S000203 1637 TTATTT
(+) POLASIG3 S000088 1638 AATAAT

1651 CTTATTAATATTCAGAAATTCAAATATGACCACTTTCATATTTTAATTTT
(+) TATABOX3 S000110 1653 TATTAAT
(-) TATABOX3 S000110 1654 TATTAAT

```
        (-) ROOTMOTIFTAPOX1 S000098 1657 ATATT
         (+) ROOTMOTIFTAPOX1 S000098 1658 ATATT
               (+) POLLEN1LELAT52 S000245 1664 AGAAA
                (-) EECCRCAH1 S000494 1665 GANTTNC
                  (+) ERELEE4 S000037 1667 AWTTCAAA
                        (-) ROOTMOTIFTAPOX1 S000098 1673 ATATT
                            (+) WRKY71OS S000447 1677 TGAC
                            (+) WBOXNTERF3 S000457 1677 TGACY
                                (+) CACTFTPPCA1 S000449 1681 YACT
                                  (-) DOFCOREZM S000265 1683 AAAG
                                       (-) LECPLEACS2 S000465 1688 TAAAATAT
                                       (+) ROOTMOTIFTAPOX1 S000098 1688 ATATT
                                           (-) POLASIG2 S000081 1692 AATTAAA
                                                  (-) GT1CONSENSUS S000198 1699 GRWAAW
                                                  (-) GT1GMSCAM4 S000453 1699 GAAAAA

1701   TTTCATTTTTTGCTACAGCTCACCTATAGGGCATTGGCTCACTGAAATTT
        (+) INRNTPSADB S000395 1702 YTCANTYY
                      (-) GTGANTG10 S000378 1720 GTGA
                                  (-) CAATBOX1 S000028 1733 CAAT
                                  (-) CCAATBOX1 S000030 1733 CCAAT
                                        (-) GTGANTG10 S000378 1739 GTGA
                                         (+) CACTFTPPCA1 S000449 1740 YACT

1751   TTTGTTTTATTAGTTTCCATCATGATTCTATATATTTTGATGCCTGAATT
       (-) ANAERO1CONSENSUS S000477 1751 AAACAAA
            (-) POLASIG1 S000080 1756 AATAAA
              (+) CPBCSPOR S000491 1758 TATTAG
                             (+) ARR1AT S000454 1773 NGATT
                                (-) BOXIINTPATPB S000296 1776 ATAGAA
                                      (+) ROOTMOTIFTAPOX1 S000098 1782 ATATT
                                                       (+) MARTBOX S000067 1799 TTWTWTTWTT
                                                        (+) MARTBOX S000067 1800 TTWTWTTWTT

1801   TTTTTTTTTCTCCTAGTCTATATAAAATTTATATATGAGAGAACTAAAAT
           (-) GT1CONSENSUS S000198 1805 GRWAAW
           (-) GT1GMSCAM4 S000453 1805 GAAAAA
             (-) POLLEN1LELAT52 S000245 1807 AGAAA
                         (-) TATAPVTRNALEU S000340 1819 TTTATATA
                         (+) TATABOX4 S000111 1819 TATATAA
                                 (-) TATABOX2 S000109 1827 TATAAAT
                                  (+) TATAPVTRNALEU S000340 1828 TTTATATA
                                   (-) TATABOX4 S000111 1829 TATATAA
                                                  (-) CARGCW8GAT S000431 1844 CWWWWWWWWG
                                                  (+) CARGCW8GAT S000431 1844 CWWWWWWWWG

1851   TTGATATGAAATATATTAATAGTCTTATAATTATTTAATTATTTATAATA
         (+) GATABOX S000039 1853 GATA
                (-) ROOTMOTIFTAPOX1 S000098 1860 ATATT
```

(+) ROOTMOTIFTAPOX1 S000098 1863 ATATT
(+) TATABOX3 S000110 1864 TATTAAT
(-) TATABOX3 S000110 1865 TATTAAT
(-) POLASIG3 S000088 1880 AATAAT
(+) TATABOX5 S000203 1881 TTATTT
(+) TATABOXOSPAL S000400 1882 TATTTAA
(-) POLASIG2 S000081 1884 AATTAAA
(-) POLASIG3 S000088 1888 AATAAT
(+) TATABOX5 S000203 1889 TTATTT
(-) TATABOX2 S000109 1891 TATAAAT
(-) MARTBOX S000067 1897 TTWTWTTWTT
(+) POLASIG1 S000080 1897 AATAAA

1901 AAATAATCTGAAAATCTTCCAATGAGATTATTACATTTAAAAATTGTTCA
(-) TATABOX5 S000203 1901 TTATTT
(+) POLASIG3 S000088 1902 AATAAT
(-) ARR1AT S000454 1905 NGATT
(-) EECCRCAH1 S000494 1910 GANTTNC
(+) GT1CONSENSUS S000198 1910 GRWAAW
(-) ARR1AT S000454 1913 NGATT
(+) CCAATBOX1 S000030 1919 CCAAT
(+) CAATBOX1 S000028 1920 CAAT
(+) ARR1AT S000454 1925 NGATT
(-) POLASIG3 S000088 1927 AATAAT
(-) L1BOXATPDF1 S000386 1932 TAAATGYA
(-) SEF4MOTIFGM7S S000103 1938 RTTTTTR
(-) CAATBOX1 S000028 1943 CAAT
(+) CAATBOX1 S000028 1949 CAAT

1951 ATTATAATGTATAAGCTAAAGTGAAAAAAAGTTGATATTTATTCCACTAT
(-) NTBBF1ARROLB S000273 1967 ACTTTA
(+) TAAAGSTKST1 S000387 1967 TAAAG
(-) INRNTPSADB S000395 1968 YTCANTYY
(+) DOFCOREZM S000265 1968 AAAG
(-) CACTFTPPCA1 S000449 1970 YACT
(+) GTGANTG10 S000378 1971 GTGA
(+) GT1CONSENSUS S000198 1973 GRWAAW
(+) GT1GMSCAM4 S000453 1973 GAAAAA
(+) DOFCOREZM S000265 1978 AAAG
(+) GATABOX S000039 1984 GATA
(+) SEF1MOTIF S000006 1985 ATATTTAWW
(+) ROOTMOTIFTAPOX1 S000098 1985 ATATT
(-) POLASIG1 S000080 1988 AATAAA
(+) CACTFTPPCA1 S000449 1995 YACT
(+) ROOTMOTIFTAPOX1 S000098 1999 ATATT

2001 ATTACTTTAATTGATTGGTAATACTAAACTAAAGACAACATATTGATTAA
(+) CACTFTPPCA1 S000449 2003 YACT
(+) NTBBF1ARROLB S000273 2004 ACTTTA

(-) DOFCOREZM S000265 2005 AAAG
(-) TAAAGSTKST1 S000387 2005 TAAAG
(-) POLASIG2 S000081 2006 AATTAAA
(-) CAATBOX1 S000028 2010 CAAT
(+) ARR1AT S000454 2012 NGATT
(-) CAATBOX1 S000028 2014 CAAT
(-) CCAATBOX1 S000030 2014 CCAAT
(+) GT1CONSENSUS S000198 2017 GRWAAW
(+) CACTFTPPCA1 S000449 2022 YACT
(+) TAAAGSTKST1 S000387 2030 TAAAG
(+) DOFCOREZM S000265 2031 AAAG
(+) RAV1AAT S000314 2036 CAACA
(+) ROOTMOTIFTAPOX1 S000098 2040 ATATT
(-) CAATBOX1 S000028 2042 CAAT
(+) ARR1AT S000454 2044 NGATT

2051 AAAAACAAAACTAAAGACAACATATAAAATAAATATATAATTATTTATTT
(+) ANAERO1CONSENSUS S000477 2053 AAACAAA
(+) TAAAGSTKST1 S000387 2062 TAAAG
(+) DOFCOREZM S000265 2063 AAAG
(+) RAV1AAT S000314 2068 CAACA
(-) TATABOX5 S000203 2077 TTATTT
(-) SEF1MOTIF S000006 2078 ATATTTAWW
(+) POLASIG1 S000080 2078 AATAAA
(-) ROOTMOTIFTAPOX1 S000098 2082 ATATT
(+) TATABOX4 S000111 2084 TATATAA
(-) POLASIG3 S000088 2090 AATAAT
(+) TATABOX5 S000203 2091 TTATTT
(-) POLASIG1 S000080 2094 AATAAA
(+) TATABOX5 S000203 2095 TTATTT
(-) -300ELEMENT S000122 2097 TGHAAARK
(-) GT1CONSENSUS S000198 2098 GRWAAW
(-) GT1GMSCAM4 S000453 2098 GAAAAA

2101 TTCAATTTTTTGTGAGGATATAGTGAATGATATAATTAAATCTTGCACAT
(+) INRNTPSADB S000395 2101 YTCANTYY
(-) CARGCW8GAT S000431 2103 CWWWWWWWWG
(+) CAATBOX1 S000028 2103 CAAT
(+) CARGCW8GAT S000431 2103 CWWWWWWWWG
(+) GTGANTG10 S000378 2112 GTGA
(+) MYBST1 S000180 2116 GGATA
(+) GATABOX S000039 2117 GATA
(-) CACTFTPPCA1 S000449 2122 YACT
(+) GTGANTG10 S000378 2123 GTGA
(+) GATABOX S000039 2129 GATA
(+) POLASIG2 S000081 2134 AATTAAA
(-) ARR1AT S000454 2139 NGATT
(-) CAATBOX1 S000028 2149 CAAT
(+) WBOXATNPR1 S000390 2150 TTGAC

```
2151   TGACAGAGTTTTAATAAATTGAAATTTTAACATAATTAATTTTCTCAAAA
       (-) BIHD1OS S000498 2151 TGTCA
       (+) WRKY71OS S000447 2151 TGAC
                   (+) POLASIG1 S000080 2163 AATAAA
                      (-) INRNTPSADB S000395 2166 YTCANTYY
                        (-) CAATBOX1 S000028 2168 CAAT
                                             (-) GT1CONSENSUS S000198 2189 GRWAAW
                                               (-) POLLEN1LELAT52 S000245 2191 AGAAA
                                                      (+) DOFCOREZM S000265 2198 AAAG
                                                        (-) CACTFTPPCA1 S000449 2200 YACT

2201   GTATATTTATTCATATGCTTATGAGTCAATATTTTGTAAGTAAAAAAATA
         (+) SEF1MOTIF S000006 2203 ATATTTAWW
         (+) ROOTMOTIFTAPOX1 S000098 2203 ATATT
            (-) POLASIG1 S000080 2206 AATAAA
                  (-) EBOXBNNAPA S000144 2212 CANNTG
                  (-) CATATGGMSAUR S000370 2212 CATATG
                  (-) MYCCONSENSUSAT S000407 2212 CANNTG
                  (+) EBOXBNNAPA S000144 2212 CANNTG
                  (+) CATATGGMSAUR S000370 2212 CATATG
                  (+) MYCCONSENSUSAT S000407 2212 CANNTG
                           (-) PREATPRODH S000450 2221 ACTCAT
                            (+) GCN4OSGLUB1 S000277 2222 TGAGTCA
                              (-) WBOXHVISO1 S000442 2224 TGACT
                              (-) WBOXNTERF3 S000457 2224 TGACY
                               (-) WBOXATNPR1 S000390 2225 TTGAC
                               (-) WRKY71OS S000447 2225 TGAC
                                 (-) CARGCW8GAT S000431 2227 CWWWWWWWWG
                                 (+) CAATBOX1 S000028 2227 CAAT
                                 (+) CARGCW8GAT S000431 2227 CWWWWWWWWG
                                  (-) ROOTMOTIFTAPOX1 S000098 2228 ATATT
                                   (+) ROOTMOTIFTAPOX1 S000098 2229 ATATT
                                             (-) CACTFTPPCA1 S000449 2239 YACT
                                                     (-) ROOTMOTIFTAPOX1 S000098 2247 ATATT
                                                      (+) ROOTMOTIFTAPOX1 S000098 2248 ATATT

2251   TTTTTTTTAAATATTTTATAATACAAGAAGTAAAACTTTTGCTCGCATTA
            (-) SEF1MOTIF S000006 2256 ATATTTAWW
             (-) TATABOXOSPAL S000400 2257 TATTTAA
                (-) ROOTMOTIFTAPOX1 S000098 2260 ATATT
                 (-) LECPLEACS2 S000465 2261 TAAAATAT
                 (+) ROOTMOTIFTAPOX1 S000098 2261 ATATT
                                   (-) CACTFTPPCA1 S000449 2279 YACT
                                          (-) DOFCOREZM S000265 2286 AAAG
                                                        (+) POLLEN1LELAT52 S000245 2300 AGAAA

2301   GAAATTATAGCTCCACCACTTTTCAAACCAAATCAAAAAATTGTATTGAA
                    (+) CACTFTPPCA1 S000449 2317 YACT
```

```
                  (-) -300ELEMENT S000122 2318 TGHAAARK
                   (-) DOFCOREZM S000265 2319 AAAG
                         (+) MYB1AT S000408 2325 WAACCA
                          (+) REALPHALGLHCB21 S000362 2326 AACCAA
                               (-) ARR1AT S000454 2331 NGATT
                                  (-) CARGCW8GAT S000431 2334 CWWWWWWWWG
                                  (+) CARGCW8GAT S000431 2334 CWWWWWWWWG
                                        (-) CAATBOX1 S000028 2340 CAAT
                                             (-) CAATBOX1 S000028 2345 CAAT

2351   CCGATTTTTTTTTGTCCAGTTTAATTCTTCACTGAAATTGAATTGAAACG
        (+) ARR1AT S000454 2352 NGATT
                         (-) POLASIG2 S000081 2370 AATTAAA
                                  (-) GTGANTG10 S000378 2379 GTGA
                                   (+) CACTFTPPCA1 S000449 2380 YACT
                                       (-) INRNTPSADB S000395 2385 YTCANTYY
                                         (-) CAATBOX1 S000028 2387 CAAT
                                            (-) INRNTPSADB S000395 2390 YTCANTYY
                                              (-) CAATBOX1 S000028 2392 CAAT
                                                   (+) MYBCOREATCYCB1 S000502 2397 AACGG

2401   GTACATCAATGCTAGACCTATATCAAATCAAAAATGAATCGTGCATATGT
       (-) CURECORECR S000493 2401 GTAC
       (+) CURECORECR S000493 2401 GTAC
             (+) CAATBOX1 S000028 2407 CAAT
                           (-) GATABOX S000039 2421 GATA
                                (-) ARR1AT S000454 2426 NGATT
                                   (-) SEF4MOTIFGM7S S000103 2429 RTTTTTR
                                     (-) INRNTPSADB S000395 2431 YTCANTYY
                                           (-) ARR1AT S000454 2437 NGATT
                                             (-) RHERPATEXPA7 S000512 2439 KCACGW
                                                  (-) EBOXBNNAPA S000144 2444 CANNTG
                                                  (-) CATATGGMSAUR S000370 2444 CATATG
                                                  (-) MYCCONSENSUSAT S000407 2444 CANNTG
                                                  (+) EBOXBNNAPA S000144 2444 CANNTG
                                                  (+) CATATGGMSAUR S000370 2444 CATATG
                                                  (+) MYCCONSENSUSAT S000407 2444 CANNTG
                                                      (-) RAV1AAT S000314 2448 CAACA

2451   TGTTTTAATCATTTTATAAATATATTATATATTTTGATGGTTATATATAT
             (-) ARR1AT S000454 2457 NGATT
                     (-) SEF1MOTIF S000006 2465 ATATTTAWW
                     (+) TATABOX2 S000109 2465 TATAAAT
                         (-) ROOTMOTIFTAPOX1 S000098 2469 ATATT
                            (+) ROOTMOTIFTAPOX1 S000098 2472 ATATT
                               (-) TATABOX4 S000111 2475 TATATAA
                                   (+) ROOTMOTIFTAPOX1 S000098 2479 ATATT
                                          (-) MYB1AT S000408 2488 WAACCA
                                             (-) TATABOX4 S000111 2491 TATATAA
```

(+) TATABOX4 S000111 2500 TATATAA

2501 ATATAATATATATATATATATATATATATATATATATATATATTATAATATA
(-) ROOTMOTIFTAPOX1 S000098 2505 ATATT
(+) ROOTMOTIFTAPOX1 S000098 2538 ATATT
(-) ROOTMOTIFTAPOX1 S000098 2545 ATATT
(+) ROOTMOTIFTAPOX1 S000098 2550 ATATT

2551 TATTATATGATTATGGCTTATATAACTTAATATTATCTATCATTCTTCTA
(+) ARR1AT S000454 2558 NGATT
(-) TATABOX4 S000111 2568 TATATAA
(+) TATABOX4 S000111 2569 TATATAA
(-) ROOTMOTIFTAPOX1 S000098 2579 ATATT
(+) ROOTMOTIFTAPOX1 S000098 2580 ATATT
(-) GT1CONSENSUS S000198 2582 GRWAAW
(-) IBOXCORE S000199 2583 GATAA
(-) GATABOX S000039 2584 GATA
(-) GATABOX S000039 2588 GATA
(-) BOXIINTPATPB S000296 2596 ATAGAA

2601 TTTTTTTTAATGATTTTAGTTACAAATATATTAGATCACTTTATAATACT
(+) ARR1AT S000454 2611 NGATT
(-) ROOTMOTIFTAPOX1 S000098 2625 ATATT
(+) ROOTMOTIFTAPOX1 S000098 2628 ATATT
(+) CPBCSPOR S000491 2629 TATTAG
(-) GTGANTG10 S000378 2636 GTGA
(+) CACTFTPPCA1 S000449 2637 YACT
(+) NTBBF1ARROLB S000273 2638 ACTTTA
(-) DOFCOREZM S000265 2639 AAAG
(-) TAAAGSTKST1 S000387 2639 TAAAG
(+) CACTFTPPCA1 S000449 2647 YACT

2651 TAATTATTAGTGTTCTATTATACTATATAATATACTTAATGTTATGACCA
(-) HDZIP2ATATHB2 S000373 2651 TAATMATTA
(-) POLASIG3 S000088 2653 AATAAT
(+) CPBCSPOR S000491 2655 TATTAG
(-) CACTFTPPCA1 S000449 2659 YACT
(-) BOXIINTPATPB S000296 2663 ATAGAA
(+) CACTFTPPCA1 S000449 2671 YACT
(+) TATABOX4 S000111 2674 TATATAA
(-) ROOTMOTIFTAPOX1 S000098 2679 ATATT
(+) CACTFTPPCA1 S000449 2683 YACT
(+) WRKY71OS S000447 2695 TGAC
(+) WBOXNTERF3 S000457 2695 TGACY

2701 GCACGCCAATTCAAGGCACACACACAAATCATACAATCACACAGTATAAA
(+) CCAATBOX1 S000030 2706 CCAAT
(+) CAATBOX1 S000028 2707 CAAT
(-) ARR1AT S000454 2727 NGATT

(+) CAATBOX1 S000028 2734 CAAT
(-) ARR1AT S000454 2735 NGATT
(-) GTGANTG10 S000378 2737 GTGA
(-) CACTFTPPCA1 S000449 2743 YACT
(+) DOFCOREZM S000265 2750 AAAG

2751 AAGAGAAGTAGAATAACACAGAATTTAACGTGGTTCAACCGTAAGGTCTA
(-) NODCON2GM S000462 2751 CTCTT
(-) OSE2ROOTNODULE S000468 2751 CTCTT
(-) CACTFTPPCA1 S000449 2757 YACT
(-) -10PEHVPSBD S000392 2760 TATTCT
(+) T/GBOXATPIN2 S000458 2777 AACGTG
(+) ABRERATCAL S000507 2777 MACGYGB
(-) ACGTATERD1 S000415 2778 ACGT
(+) ABRELATERD1 S000414 2778 ACGTG
(+) ACGTATERD1 S000415 2778 ACGT
(-) MYBCORE S000176 2786 CNGTTR

2801 CATCCACTGGAAAGATAAGAGAAAAAGTTCTACTATGATGAAAAGAGTAA
(+) CACTFTPPCA1 S000449 2805 YACT
(+) DOFCOREZM S000265 2811 AAAG
(+) NODCON1GM S000461 2811 AAAGAT
(+) OSE1ROOTNODULE S000467 2811 AAAGAT
(+) GATABOX S000039 2814 GATA
(+) IBOX S000124 2814 GATAAG
(+) IBOXCORE S000199 2814 GATAA
(+) IBOXCORENT S000424 2814 GATAAGR
(-) NODCON2GM S000462 2817 CTCTT
(-) OSE2ROOTNODULE S000468 2817 CTCTT
(+) POLLEN1LELAT52 S000245 2820 AGAAA
(+) GT1CONSENSUS S000198 2821 GRWAAW
(+) GT1GMSCAM4 S000453 2821 GAAAAA
(+) DOFCOREZM S000265 2824 AAAG
(+) CACTFTPPCA1 S000449 2831 YACT
(+) DOFCOREZM S000265 2842 AAAG
(-) NODCON2GM S000462 2843 CTCTT
(-) OSE2ROOTNODULE S000468 2843 CTCTT
(-) CACTFTPPCA1 S000449 2846 YACT

2851 GATACAATAACTAAAAACTTTCAAACCCTAACCCCAATGTGTTTTCCGAA
(+) GATABOX S000039 2851 GATA
(+) CAATBOX1 S000028 2855 CAAT
(-) SEF4MOTIFGM7S S000103 2862 RTTTTTR
(-) DOFCOREZM S000265 2868 AAAG
(+) TELOBOXATEEF1AA1 S000308 2873 AAACCCTAA
(+) UP2ATMSD S000472 2873 AAACCCTA
(+) CCAATBOX1 S000030 2884 CCAAT
(+) LEAFYATAG S000432 2884 CCAATGT
(+) CAATBOX1 S000028 2885 CAAT

(-) GT1CONSENSUS S000198 2892 GRWAAW

2901 CATCTCACAACTCTAATATTTTATCCTAATCGGGTCGCTGCGCGAAACTT
(-) GTGANTG10 S000378 2905 GTGA
(+) CAREOSREP1 S000421 2908 CAACTC
(-) CPBCSPOR S000491 2913 TATTAG
(-) ROOTMOTIFTAPOX1 S000098 2915 ATATT
(-) LECPLEACS2 S000465 2916 TAAAATAT
(+) ROOTMOTIFTAPOX1 S000098 2916 ATATT
(-) GT1CONSENSUS S000198 2920 GRWAAW
(-) IBOXCORE S000199 2921 GATAA
(+) SREATMSD S000470 2921 TTATCC
(-) GATABOX S000039 2922 GATA
(-) MYBST1 S000180 2922 GGATA
(-) ARR1AT S000454 2928 NGATT
(-) E2FCONSENSUS S000476 2940 WTTSSCSS
(-) HSELIKENTACIDICPR1 S000056 2941 CNNGAANNNTTCNNG
(+) HSELIKENTACIDICPR1 S000056 2941 CNNGAANNNTTCNNG
(-) DOFCOREZM S000265 2948 AAAG

2951 TCAAGTTGGGTCACAATTCTAGTCCATGAAAAACATATCCAAAATTCAAG
(-) WBOXNTERF3 S000457 2959 TGACY
(-) WRKY71OS S000447 2960 TGAC
(-) GTGANTG10 S000378 2961 GTGA
(+) CAATBOX1 S000028 2964 CAAT
(+) GT1CONSENSUS S000198 2978 GRWAAW
(+) GT1GMSCAM4 S000453 2978 GAAAAA
(-) GATABOX S000039 2986 GATA
(-) MYBST1 S000180 2986 GGATA
(+) TATCCAOSAMY S000403 2986 TATCCA
(+) SORLIP1AT S000482 3000 GCCAC

Supplementary Figure 7:

The secondary structure prediction of Uncharacterized protein LOC110648447 isoforms using PSIPRED server

1. Uncharacterized protein LOC110648447 isoform X1

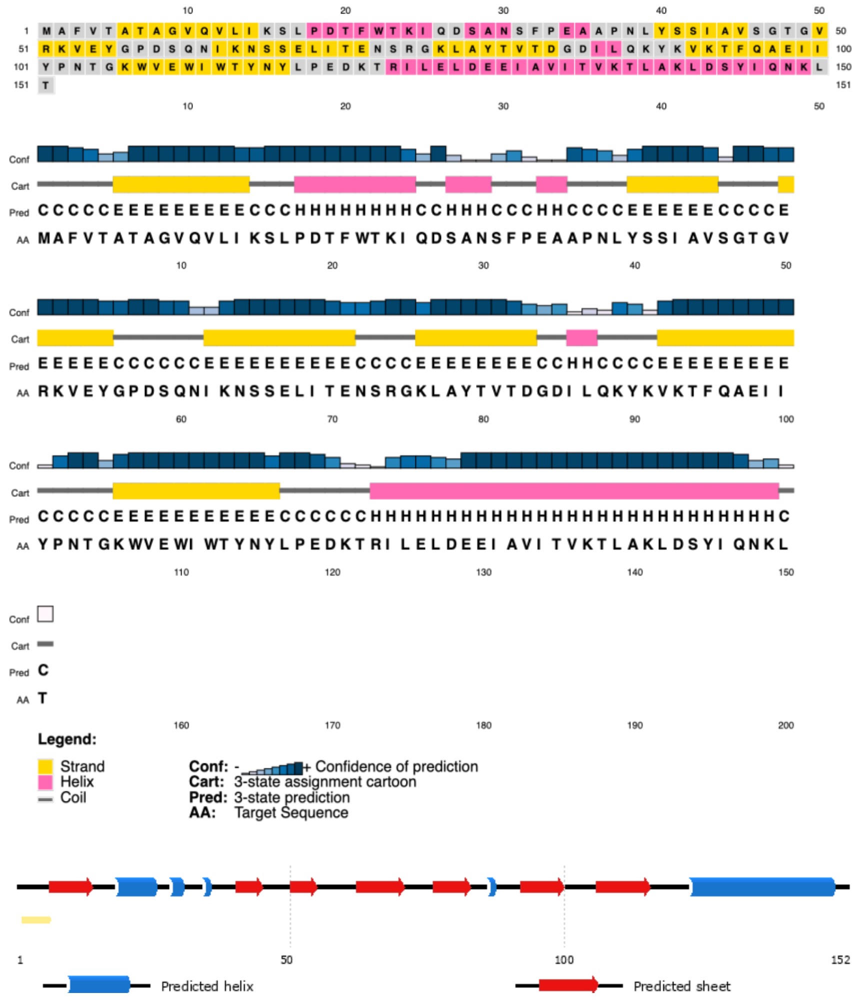

2. Uncharacterized protein LOC110648447 isoform X2

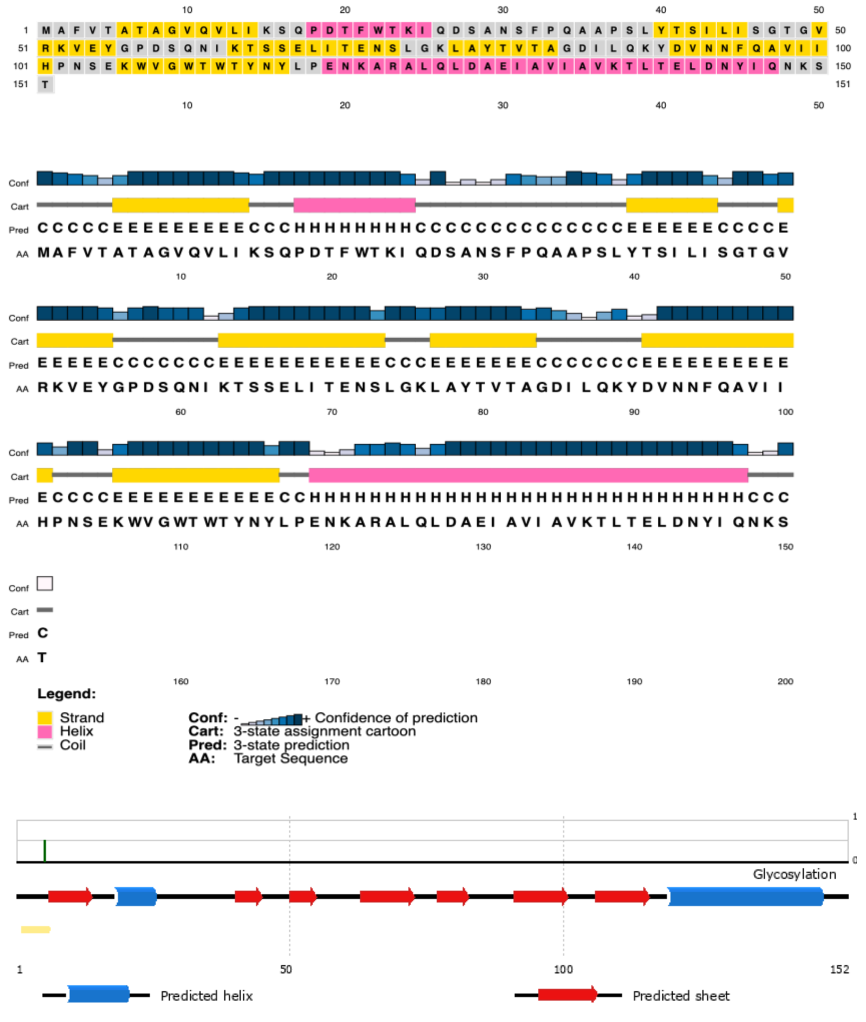

3. Uncharacterized protein LOC110648447 isoform X3

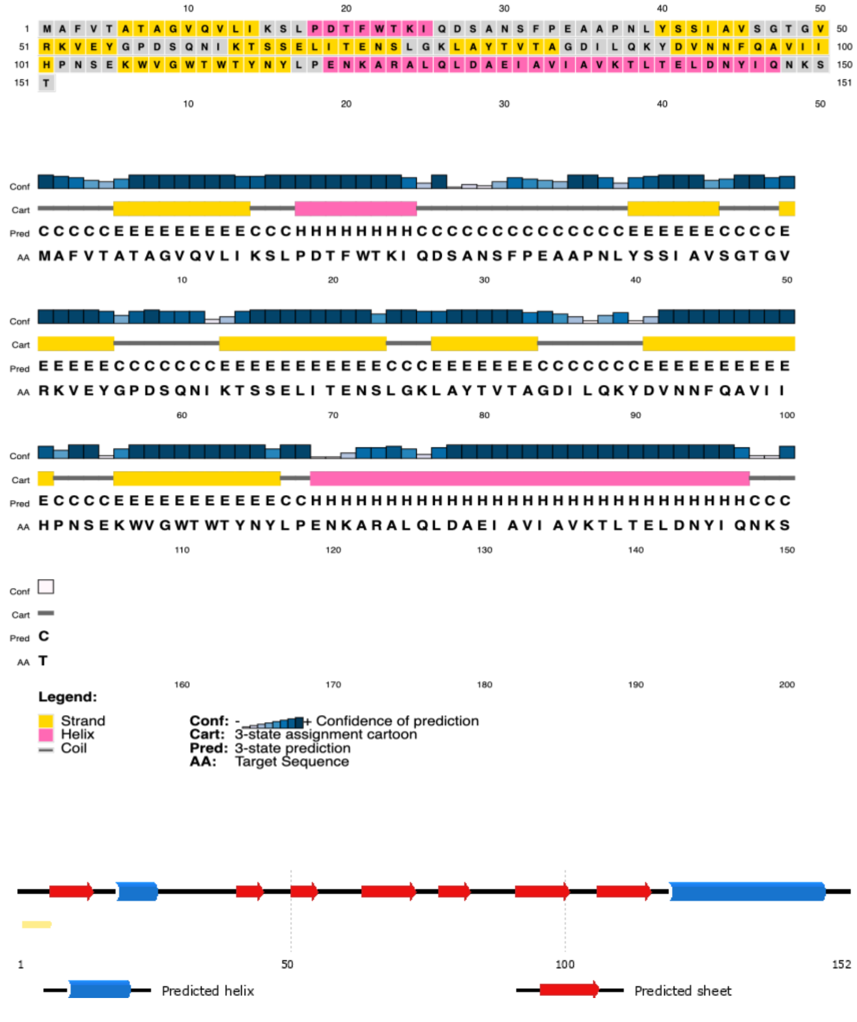

Supplementary Figure 8:

The secondary structure prediction of Uncharacterized protein LOC110648447 isoforms using I-TASSER server

I-TASSER protein predictions

(https://zhanglab.ccmb.med.umich.edu)

Isoform X1 : https://zhanggroup.org/I-TASSER/output/S652592/ Submission date 2021-10-26

Isoform X2 : https://zhanggroup.org/I-TASSER/output/S653021/ Submission date 2021-10-28

Isoform X3 : https://zhanggroup.org/I-TASSER/output/S654214/ Submission date 2021-11-09

Note that the results are kept on the server for 60 days

Secondary structure	H:Helix; S:Strand; C:Coil
Solvent accessibility (Sol. Acc.)	Values range from **0** (buried residue) to **9** (highly exposed residue)

Isoform X1

```
                     20        40        60        80        100       120       140
                      |         |         |         |         |         |         |
Sequence             MAFVTATAGVQVLIKSLPDTFWTKIQDSANSFPEAAPNLYSSIAVSGTGVRKVEYGPDSQNIKNSSELITENSRGKLAYTVTDGDILQKYKVKTFQAEIIYPNTGKWVEWIWTYNYLPEDKTRILELDEEIAVITVKTLAKLDSYIQNKLT
Prediction           CCCCSSSSSSSSSSCCCHHHHHHHHCCCCHHCCHHCCCCSSSSSSSCCCSSSSSSCCCCCSSSSSSSSSSSCCCCSSSSSSSCCCCCCCCCSSSSSSSSSSCCCCCSSSSSSSSSSSCCCCCCCCCCHHHHHHHHHHHHHHHHHHHHHCCC
Confidence Score     9641389999999617999999987282001620255211489973685899999799726888999999478888999999857420032127999999988999869999999999668877667987899999999999999998864579
Solution Accessibility 7442423031414141306301410363441014213531441424443123031364343143334413367433030302434136315423230302037734202030303134763746264264124202510430152047448
```

Isoform X2

```
                     20        40        60        80        100       120       140
                      |         |         |         |         |         |         |
Sequence             MAFVTATAGVQVLIKSQPDTFWTKIQDSANSFPQAAPSLYTSILISGTGVRKVEYGPDSQNIKTSSELITENSLGKLAYTVTAGDILQKYDVNNFQAVIIHPNSEKWVGWTWTYNYLPENKARALQLDAEIAVIAVKTLTELDNYIQNKST
Prediction           CCSSSSSSSSSSSSCCCHHHHHHHHCCCCCCCCCCCCHHSSSSSSSCCCSSSSSSCCCCCSSSSSSSSSSSCCCCSSSSSSSCCCCCCCSSSSSSSSSSSCCCCCSSSSSSSSSSSCCCCCCCCCCCCHHHHHHHHHHHHHHHHHHHCCCC
Confidence Score     9225789999999616999999987275332651282034479985790799799899748889999998167878999999044400212379899999879997799999999995587677656880789999999999999999830799
Solution Accessibility 6331323131413141305301410274432124113531441314343123020244343143334413367433120202324126315414130301147632202010303334654466363464024202410430052037468
```

Isoform X3

```
                     20        40        60        80        100       120       140
                      |         |         |         |         |         |         |
Sequence             MAFVTATAGVQVLIKSLPDTFWTKIQDSANSFPEAAPNLYSSIAVSGTGVRKVEYGPDSQNIKTSSELITENSLGKLAYTVTAGDILQKYDVNNFQAVIIHPNSEKWVGWTWTYNYLPENKARALQLDAEIAVIAVKTLTELDNYIQNKST
Prediction           CCSSSSSSSSSSSSCCCHHHHHHHHCCCCCCCCCCCCHHHSSSSSSCCSSSSSSSCCCCCSSSSSSSSSSSCCCCSSSSSSSSCCCCCCSSSSSSSSSSSCCCCCSSSSSSSSSSSCCCCCCCCCCCCHHHHHHHHHHHHHHHHHHHHHCCC
Confidence Score     92367899999999617999999987376531662272230047764783899899899628899999999845678899999991343002213799999998699987999999999965877777658807899999999999999999830799
Solution Accessibility 6331324131413141305301410373432024213530451414343023031244343143335414367433020202324025314414130302147632202010303335764366463364025202410430052037468
```

Supplementary Figure 9:

The predicted normalized B-factor of predicted secondary structure of Uncharacterized protein LOC110648447 isoforms using I-TASSER server

B-factor is a value to indicate the extent of the inherent thermal mobility of residues/atoms in proteins. In I-TASSER, this value is deduced from threading template proteins from the PDB in combination with the sequence profiles derived from sequence databases. The reported B-factor profile in the figure below corresponds to the normalized B-factor of the target protein, defined by B=(B'-u)/s, where B' is the raw B-factor value, u and s are respectively the mean and standard deviation of the raw B-factors along the sequence.

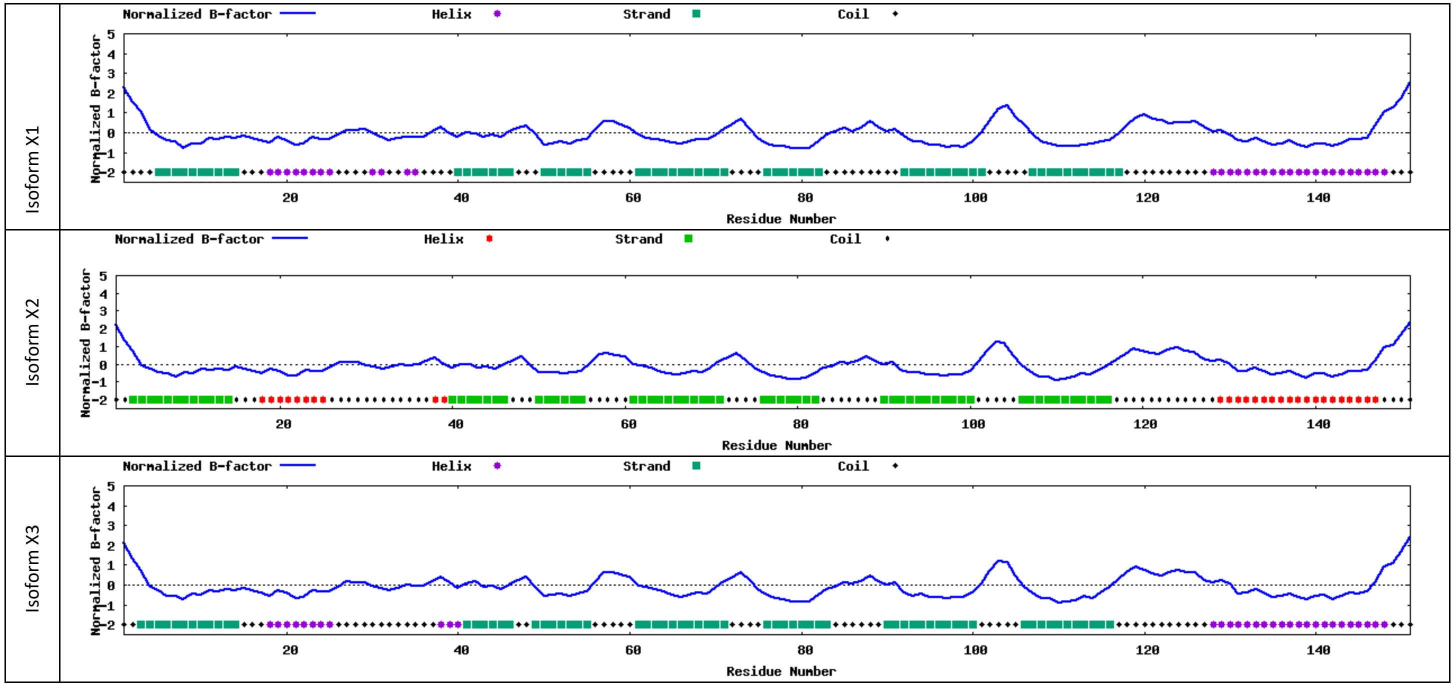

Supplementary Tables

Supplementary Table 1:

Identification results of the selected 1D-PAGE band and 2D-PAGE spots by ESI-QUAD-TOF mass spectrometry (MS/MS) analysis

ID	Protein name	Genbank	Theoritical pI/MW	Observed pI/MW	Hit peptide fragments	Sequence coverage (%)	emPAI	Fold changes*
1D band	Uncharacterized protein LOC110648447 isoform X1 [*H. brasiliensis*]	XP_021658364.1	5.2/17.0	-/18.0	K.LDSYIQNK.L<br>K.NSSELITENSR.G<br>R.KVEYGPDSQNIK.N<br>K.LAYTVTDGDILQK.Y<br>K.TFQAEIIYPNTGK.W<br>R.GKLAYTVTDGDILQK.Y<br>K.IQDSANSFPEAAPNLYSSIAVSGTGVR.K	56.9	5.25	n/a
	Uncharacterized protein LOC110648447 isoform X3 [*H. brasiliensis*]	XP_021658366.1	5.1/16.6		R.KVEYGPDSQNIK.T<br>K.TSSELITENSLGK.L<br>K.LAYTVTAGDILQK.Y<br>K.TLTELDNYIQNK.S<br>K.YDVNNFQAVIIHPNSEK.W<br>K.IQDSANSFPEAAPNLYSSIAVSGTGVR.K	62.0	3.96	
	Oxygen-evolving enhancer protein 3-2, chloroplastic-like [*H. brasiliensis*]	XP_021639605.1	9.5/24.4		R.YDLNTVISAK.S<br>K.AWPYVQNDLR.L<br>K.LFQSISDLDHAAK.I<br>R.FFIQPLEPAQAAAR.A<br>K.VGPPPPPSGGLPGTLNSDQPR.D	29.9	1.51	
C1	17.3 kDa class I heat shock protein-like [*H. brasiliensis*]	XP_021675560.1	6.0/18.4	6.5/22.0	R.SELASETSAFANTR.M	8.0	0.22	-1.308
C2	Superoxide dismutase [Cu-Zn]-like [*H. brasiliensis*]	XP_021640614.1	5.6/15.4	6.4/18.0	K.HIPLSGPNSIIGR.A	8.0	0.26	-1.779
C3	ATP synthase subunit D, mitochondrial-like [*H. brasiliensis*]	XP_021666672.1	5.1/19.8	5.3/26.5	K.KVTDVAFK.A<br>K.EAYDSIEVPK.Y<br>R.AFDEVNSTLQTK.F<br>K.YVDTVTPQYKPK.F	29.0	3.2	-2.597

ID	Protein name	Genbank	Theoritical pI/MW	Observed pI/MW	Hit peptide fragments	Sequence coverage (%)	emPAI	Fold changes*
					R.RAFDEVNSTLQTK.F<br>K.FSQEPEPIDWEYYR.K<br>R.LVDMYKEAYDSIEVPK.Y			
C4	Universal stress protein PHOS34<br>[*H. brasiliensis*]	XP_021668720.1	5.7/18.3	6.0/20.0	R.VGVAVDFSPCSRK.A	18.0	0.21	-1.847
	Probable phospholipid hydroperoxide glutathione peroxidase<br>[*H. brasiliensis*]	XP_021674982.1	8.8/26.5		R.GNDVDLSTYKGK.V	5.0	0.21	
C5	Putative cytochrome c oxidase subunit II PS17 (Fragments)<br>[*Pinus strobus*]	PS17_PINST	9.6/17.1	5.9/18.5	R.VVEALSPR.-	50.0	3.44	-8.712
N1	Uncharacterized protein LOC110648447 isoform X2 and isoform X3<br>[*H. brasiliensis*]<br>(Protein matching the same set of peptides)	XP_021658365.1<br>XP_021658366.1	5.3/16.7<br>5.1/16.6	4.7/17.7	K.KVEYGPDSQNIK.T<br>K.TSSELITENSLGK.L<br>K.LAYTVTAGDILQK.Y<br>K.TLTELDNYIQNK.S<br>K.YDVNNFQAVIIHPNSEK.W	35.0	1.87	Undetectable in control treatment
	Uncharacterized protein LOC110648447 isoform X1<br>[*H. brasiliensis*]	XP_021658366.1	5.2/17.0		K.NSSELITENSR.G<br>R.KVEYGPDSQNIK.N<br>K.LAYTVTDGDILQK.Y<br>K.TFQAEIIYPNTGK.W	32.5	1.29	
	Peroxiredoxin-2E-2, chloroplastic-like<br>[*H. brasiliensis*]	XP_021644838.1	8.4/24.3		R.YALLAEDGVVK.V<br>K.SIFFAVPGAFTPTCSQK.H	12.2	0.34	
N2	Uncharacterized protein LOC110648447 isoform X1<br>[*H. brasiliensis*]	XP_021658364.1	5.2/17.0	4.9/17.3	K.NSSELITENSR.G<br>R.KVEYGPDSQNIK.N<br>K.TLAKLDSYIQNK.L<br>K.LAYTVTDGDILQK.Y<br>K.TFQAEIIYPNTGK.W	60.0	4.25	Undetectable in control treatment

ID	Protein name	Genbank	Theoritical pI/MW	Observed pI/MW	Hit peptide fragments	Sequence coverage (%)	emPAI	Fold changes*
					R.ILELDEEIAVITVK.T<br>R.GKLAYTVTDGDILQK.Y<br>K.IQDSANSFPEAAPNLYSSIAVSGTGVR.K			
	Uncharacterized protein LOC110648447 isoform X3 [*H. brasiliensis*]	XP_021658366.1	5.1/16.6		R.KVEYGPDSQNIK.T<br>K.TSSELITENSLGK.L<br>K.LAYTVTAGDILQK.Y<br>K.TLTELDNYIQNK.S<br>K.YDVNNFQAVIIHPNSEK.W<br>K.IQDSANSFPEAAPNLYSSIAVSGTGVR.K	62.3	2.55	
	Thylakoid luminal 17.4 kDa protein, chloroplastic isoform X1 [*H. brasiliensis*]	XP_021662359.1	6.7/25.7		K.AYAVGASFK.G<br>K.ANLQGAVFK.N<br>K.SLAAALMSDAK.F<br>K.GVDFSNAVLDR.V<br>K.FDGADMSEVVMSK.A	22.3	1.06	
N3	Uncharacterized protein LOC110648447 isoform X3 [*H. brasiliensis*]	XP_021658366.1	5.1/16.6	5.2/16.3	K.SLPDTFWTK.I<br>R.KVEYGPDSQNIK.T<br>K.TSSELITENSLGK.L<br>K.LAYTVTAGDILQK.Y<br>K.TLTELDNYIQNK.S<br>R.ALQLDAEIAVIAVK.T<br>K.WVGWTWTYNYLPENK.A<br>K.YDVNNFQAVIIHPNSEK.W<br>K.IQDSANSFPEAAPNLYSSIAVSGTGVR.K	81.0	5.69	Undetectable in control treatment
	Uncharacterized protein LOC110648447 isoform X1 [*H. brasiliensis*]	XP_021658364.1	5.2/17.0		K.SLPDTFWTK.I<br>K.NSSELITENSR.G<br>R.KVEYGPDSQNIK.N<br>K.LAYTVTDGDILQK.Y<br>K.TFQAEIIYPNTGK.W<br>R.ILELDEEIAVITVK.T<br>R.GKLAYTVTDGDILQK.Y<br>K.VKTFQAEIIYPNTGK.W<br>K.IQDSANSFPEAAPNLYSSIAVSGTGVR.K	68.2	5.46	

ID	Protein name	Genbank	Theoritical pI/MW	Observed pI/MW	Hit peptide fragments	Sequence coverage (%)	emPAI	Fold changes*
	Uncharacterized protein LOC110648447 isoform X2 [*H. brasiliensis*]	XP_021658365.1	5.3/16.7		R.KVEYGPDSQNIK.T<br>K.TSSELITENSLGK.L<br>K.LAYTVTAGDILQK.Y<br>K.TLTELDNYIQNK.S<br>R.ALQLDAEIAVIAVK.T<br>K.WVGWTWTYNYLPENK.A<br>K.YDVNNFQAVIIHPNSEK.W<br>K.IQDSANSFPQAAPSLYTSILISGTGVR.K	82.1	4.42	
	Thylakoid luminal 16.5 kDa protein, chloroplastic isoform X1 [*H. brasiliensis*]	XP_021657916.1	9.3/25.2		K.TQVDTFNSSLASLISSVTSNDIESSK.I	10.9	0.15	
N4	Aminotran_3 domaincontaining protein [*Cephalotus follicularis*]	GAV65601.1	6.4/51.0	6.3/17.0	K.SDFLNALR.S	1.0	0.07	Undetectable in control treatment
N5	Uncharacterized protein LOC110648447 isoform X1 [*H. brasiliensis*]	XP_021658364.1	5.2/17.0	5.5/17.3	K.LDSYIQNK.L<br>K.NSSELITENSR.G<br>R.KVEYGPDSQNIK.N<br>K.TLAKLDSYIQNK.L<br>K.LAYTVTDGDILQK.Y<br>K.TFQAEIIYPNTGK.W<br>K.LAYTVTDGDILQKYK.V	40.0	3.22	+2.959
	Uncharacterized protein LOC110648447 isoform X2 and isoform X3 [*H. brasiliensis*] (Protein matching the same set of peptides)	XP_021658365.1<br>XP_021658366.1	5.3/16.7<br>5.1/16.6		R.KVEYGPDSQNIK.T<br>K.LAYTVTAGDILQK.Y<br>K.TLTELDNYIQNK.S	24.5	0.87	
N6	Uncharacterized protein LOC110648447 isoform X1	XP_021658364.1	5.2/17.0	5.7/17.3	K.NSSELITENSR.G	7.0	0.22	+2.913

ID	Protein name	Genbank	Theoritical pI/MW	Observed pI/MW	Hit peptide fragments	Sequence coverage (%)	emPAI	Fold changes*
	[*H. brasiliensis*]							
N7	Metalloendoproteinase 5-MMP-like [*H. brasiliensis*]	XP_021637894.1	7.0/34.4	7.0/20.5	R.SAVQVIDEK.V<br>R.TLAHAFAPEDGR.F<br>R.HLSQDDIDGIHALYS.-<br>K.KWADVSQFTFEEAAAGSK.S	6.0	0.53	Undetectable in control treatment
	Cytochrome b6-f complex iron-sulfur subunit, chloroplastic [*H. brasiliensis*]	XP_021668850.1	7.65/24.5		K.DAIGNDIFADQWLK.T	6.6	0.16	

* : "- fold" is downregulated and "+ fold" is upregulated

Supplementary Table 2:

Location of predicted cis-acting elements on the promoter region of Uncharacterized protein LOC110648447

Factor or Site Name	Loc.(Str.)	Signal Sequence	SITE #	Factor or Site Name	Loc.(Str.)	Signal Sequence	SITE #
POLLEN1LELAT52	13 (+)	AGAAA	S000245	CARGCW8GAT	230 (+)	CWWWWWWWWG	S000431
ANAERO1CONSENSUS	15 (+)	AAACAAA	S000477	ARR1AT	241 (-)	NGATT	S000454
ANAERO1CONSENSUS	20 (+)	AAACAAA	S000477	GTGANTG10	243 (-)	GTGA	S000378
DOFCOREZM	24 (+)	AAAG	S000265	CACTFTPPCA1	248 (-)	YACT	S000449
DOFCOREZM	29 (+)	AAAG	S000265	POLASIG3	269 (+)	AATAAT	S000088
EBOXBNNAPA	33 (-)	CANNTG	S000144	ROOTMOTIFTAPOX1	272 (-)	ATATT	S000098
MYCCONSENSUSAT	33 (-)	CANNTG	S000407	TATAPVTRNALEU	274 (-)	TTTATATA	S000340
EBOXBNNAPA	33 (+)	CANNTG	S000144	TATABOX4	274 (+)	TATATAA	S000111
MYCCONSENSUSAT	33 (+)	CANNTG	S000407	TATABOX2	276 (+)	TATAAAT	S000109
NODCON2GM	39 (-)	CTCTT	S000462	POLASIG3	281 (-)	AATAAT	S000088
OSE2ROOTNODULE	39 (-)	CTCTT	S000468	TATABOX3	283 (+)	TATTAAT	S000110
POLASIG3	47 (-)	AATAAT	S000088	POLASIG2	287 (+)	AATTAAA	S000081
TATABOX5	48 (+)	TTATTT	S000203	GATABOX	296 (+)	GATA	S000039
GATABOX	62 (+)	GATA	S000039	SORLREP3AT	297 (-)	TGTATATAT	S000488
GATABOX	66 (+)	GATA	S000039	CAATBOX1	304 (+)	CAAT	S000028
RAV1AAT	82 (+)	CAACA	S000314	TATABOX5	315 (-)	TTATTT	S000203
DPBFCOREDCDC3	88 (+)	ACACNNG	S000292	ARR1AT	320 (+)	NGATT	S000454
CACTFTPPCA1	93 (-)	YACT	S000449	EECCRCAH1	321 (+)	GANTTNC	S000494
TATABOX4	97 (+)	TATATAA	S000111	GT1CONSENSUS	322 (-)	GRWAAW	S000198
GTGANTG10	112 (+)	GTGA	S000378	GATABOX	333 (+)	GATA	S000039
REALPHALGLHCB21	121 (+)	AACCAA	S000362	GT1CONSENSUS	333 (+)	GRWAAW	S000198
SEF4MOTIFGM7S	137 (-)	RTTTTTR	S000103	IBOXCORE	333 (+)	GATAA	S000199
TATABOX3	142 (-)	TATTAAT	S000110	GT1CONSENSUS	341 (+)	GRWAAW	S000198
POLASIG1	145 (+)	AATAAA	S000080	CARGCW8GAT	353 (-)	CWWWWWWWWG	S000431
GARE2OSREP1	161 (+)	TAACGTA	S000420	CARGCW8GAT	353 (+)	CWWWWWWWWG	S000431
ACGTATERD1	163 (-)	ACGT	S000415	TATABOX4	355 (+)	TATATAA	S000111
ACGTATERD1	163 (+)	ACGT	S000415	POLASIG2	367 (-)	AATTAAA	S000081
CCAATBOX1	170 (+)	CCAAT	S000030	POLLEN1LELAT52	377 (+)	AGAAA	S000245
EBOXBNNAPA	171 (-)	CANNTG	S000144	EECCRCAH1	378 (-)	GANTTNC	S000494
MYCCONSENSUSAT	171 (-)	CANNTG	S000407	TBOXATGAPB	388 (+)	ACTTTG	S000383
CAATBOX1	171 (+)	CAAT	S000028	DOFCOREZM	389 (-)	AAAG	S000265
EBOXBNNAPA	171 (+)	CANNTG	S000144	WBOXATNPR1	401 (+)	TTGAC	S000390
MYCCONSENSUSAT	171 (+)	CANNTG	S000407	HEXMOTIFTAH3H4	402 (-)	ACGTCA	S000053
CAATBOX1	173 (-)	CAAT	S000028	PALINDROMICCBOXGM	402 (-)	TGACGTCA	S000255
CCAATBOX1	173 (-)	CCAAT	S000030	ASF1MOTIFCAMV	402 (+)	TGACG	S000024
GT1CONSENSUS	177 (+)	GRWAAW	S000198	PALINDROMICCBOXGM	402 (+)	TGACGTCA	S000255
POLLEN1LELAT52	182 (-)	AGAAA	S000245	TGACGTVMAMY	402 (+)	TGACGT	S000377
CACTFTPPCA1	187 (+)	YACT	S000449	WRKY71OS	402 (+)	TGAC	S000447
CACTFTPPCA1	199 (-)	YACT	S000449	ACGTCBOX	403 (-)	GACGTC	S000131
ARR1AT	208 (-)	NGATT	S000454	ACGTCBOX	403 (+)	GACGTC	S000131
ROOTMOTIFTAPOX1	221 (-)	ATATT	S000098	TGACGTVMAMY	404 (-)	TGACGT	S000377
ROOTMOTIFTAPOX1	222 (+)	ATATT	S000098	ACGTATERD1	404 (-)	ACGT	S000415
DOFCOREZM	230 (-)	AAAG	S000265	HEXMOTIFTAH3H4	404 (+)	ACGTCA	S000053
TAAAGSTKST1	230 (-)	TAAAG	S000387	ACGTATERD1	404 (+)	ACGT	S000415
CARGCW8GAT	230 (-)	CWWWWWWWWG	S000431	ASF1MOTIFCAMV	405 (-)	TGACG	S000024

Factor or Site Name	Loc.(Str.)	Signal Sequence	SITE #
WBOXATNPR1	406 (-)	TTGAC	S000390
WRKY71OS	406 (-)	TGAC	S000447
POLASIG1	412 (-)	AATAAA	S000080
CAATBOX1	415 (-)	CAAT	S000028
CAATBOX1	421 (+)	CAAT	S000028
SEF1MOTIF	426 (-)	ATATTTAWW	S000006
POLASIG1	426 (+)	AATAAA	S000080
ROOTMOTIFTAPOX1	430 (-)	ATATT	S000098
MARTBOX	438 (+)	TTWTWTTWTT	S000067
MARTBOX	439 (+)	TTWTWTTWTT	S000067
POLASIG3	458 (-)	AATAAT	S000088
TATABOX5	459 (+)	TTATTT	S000203
GT1CONSENSUS	461 (-)	GRWAAW	S000198
GT1CONSENSUS	462 (-)	GRWAAW	S000198
CARGCW8GAT	469 (-)	CWWWWWWWWG	S000431
CARGCW8GAT	469 (+)	CWWWWWWWWG	S000431
INRNTPSADB	473 (-)	YTCANTYY	S000395
CAATBOX1	483 (-)	CAAT	S000028
CCAATBOX1	483 (-)	CCAAT	S000030
GTGANTG10	489 (-)	GTGA	S000378
ROOTMOTIFTAPOX1	493 (+)	ATATT	S000098
CARGCW8GAT	498 (-)	CWWWWWWWWG	S000431
CARGCW8GAT	498 (+)	CWWWWWWWWG	S000431
TATABOX5	500 (-)	TTATTT	S000203
POLASIG1	501 (+)	AATAAA	S000080
TAAAGSTKST1	503 (+)	TAAAG	S000387
DOFCOREZM	504 (+)	AAAG	S000265
NODCON1GM	504 (+)	AAAGAT	S000461
OSE1ROOTNODULE	504 (+)	AAAGAT	S000467
ARR1AT	506 (+)	NGATT	S000454
GATABOX	515 (+)	GATA	S000039
GATABOX	517 (-)	GATA	S000039
NAPINMOTIFBN	524 (-)	TACACAT	S000070
GATABOX	531 (+)	GATA	S000039
GT1CONSENSUS	531 (+)	GRWAAW	S000198
IBOXCORE	531 (+)	GATAA	S000199
TAAAGSTKST1	533 (+)	TAAAG	S000387
DOFCOREZM	534 (+)	AAAG	S000265
EECCRCAH1	537 (-)	GANTTNC	S000494
ARR1AT	540 (-)	NGATT	S000454
IBOXCORENT	542 (-)	GATAAGR	S000424
IBOX	543 (-)	GATAAG	S000124
IBOXCORE	544 (-)	GATAA	S000199
GATABOX	545 (-)	GATA	S000039
POLASIG1	551 (+)	AATAAA	S000080
TATABOX5	554 (-)	TTATTT	S000203
POLASIG3	555 (+)	AATAAT	S000088
POLASIG2	558 (+)	AATTAAA	S000081
TATABOXOSPAL	560 (-)	TATTTAA	S000400
TATABOX5	562 (-)	TTATTT	S000203
POLASIG1	563 (+)	AATAAA	S000080
POLASIG2	570 (-)	AATTAAA	S000081
CAATBOX1	574 (-)	CAAT	S000028
GATABOX	577 (+)	GATA	S000039
SEF4MOTIFGM7S	581 (-)	RTTTTTR	S000103
POLASIG2	585 (+)	AATTAAA	S000081
ARR1AT	590 (-)	NGATT	S000454
IBOXCORENT	592 (-)	GATAAGR	S000424
IBOX	593 (-)	GATAAG	S000124
IBOXCORE	594 (-)	GATAA	S000199
GATABOX	595 (-)	GATA	S000039
POLASIG1	601 (+)	AATAAA	S000080
TATABOX5	604 (-)	TTATTT	S000203
POLASIG3	605 (+)	AATAAT	S000088
POLASIG2	608 (+)	AATTAAA	S000081
TATABOXOSPAL	610 (-)	TATTTAA	S000400
TATABOX5	612 (-)	TTATTT	S000203
POLASIG1	613 (+)	AATAAA	S000080
POLASIG2	620 (-)	AATTAAA	S000081
CAATBOX1	624 (-)	CAAT	S000028
GATABOX	627 (+)	GATA	S000039
SEF4MOTIFGM7S	631 (-)	RTTTTTR	S000103
CACTFTPPCA1	639 (-)	YACT	S000449
SORLREP3AT	648 (-)	TGTATATAT	S000488
TATABOX5	659 (+)	TTATTT	S000203
GT1CONSENSUS	662 (-)	GRWAAW	S000198
IBOXCORE	663 (-)	GATAA	S000199
GATABOX	664 (-)	GATA	S000039
GT1CONSENSUS	668 (-)	GRWAAW	S000198
CACTFTPPCA1	697 (-)	YACT	S000449
EECCRCAH1	698 (-)	GANTTNC	S000494
INRNTPSADB	706 (+)	YTCANTYY	S000395
POLLEN1LELAT52	710 (-)	AGAAA	S000245
GT1CONSENSUS	717 (+)	GRWAAW	S000198
ROOTMOTIFTAPOX1	720 (-)	ATATT	S000098
POLLEN1LELAT52	733 (+)	AGAAA	S000245
GT1CONSENSUS	734 (+)	GRWAAW	S000198
GT1GMSCAM4	734 (+)	GAAAAA	S000453
CACTFTPPCA1	747 (-)	YACT	S000449
GTGANTG10	748 (+)	GTGA	S000378
BIHD1OS	749 (-)	TGTCA	S000498
WRKY71OS	749 (+)	TGAC	S000447
GTGANTG10	767 (+)	GTGA	S000378
ARR1AT	768 (+)	NGATT	S000454
EBOXBNNAPA	774 (-)	CANNTG	S000144
MYCCONSENSUSAT	774 (-)	CANNTG	S000407
EBOXBNNAPA	774 (+)	CANNTG	S000144
MYCCONSENSUSAT	774 (+)	CANNTG	S000407
GTGANTG10	781 (-)	GTGA	S000378
CACTFTPPCA1	782 (+)	YACT	S000449

Factor or Site Name	Loc.(Str.)	Signal Sequence	SITE #
ARR1AT	800 (+)	NGATT	S000454
CURECORECR	811 (-)	GTAC	S000493
CURECORECR	811 (+)	GTAC	S000493
CAATBOX1	814 (+)	CAAT	S000028
ROOTMOTIFTAPOX1	815 (-)	ATATT	S000098
GATABOX	817 (-)	GATA	S000039
CAATBOX1	825 (-)	CAAT	S000028
EECCRCAH1	846 (-)	GANTTNC	S000494
CAATBOX1	847 (+)	CAAT	S000028
PYRIMIDINEBOXOSRAMY1A	852 (+)	CCTTTT	S000259
DOFCOREZM	853 (-)	AAAG	S000265
CAATBOX1	861 (+)	CAAT	S000028
MARABOX1	862 (+)	AATAAAYAAA	S000063
POLASIG1	862 (+)	AATAAA	S000080
TATABOX5	865 (-)	TTATTT	S000203
MARABOX1	866 (+)	AATAAAYAAA	S000063
POLASIG1	866 (+)	AATAAA	S000080
TATABOX5	869 (-)	TTATTT	S000203
POLASIG1	870 (+)	AATAAA	S000080
REALPHALGLHCB21	878 (-)	AACCAA	S000362
MYBATRD22	879 (-)	CTAACCA	S000175
MYB1AT	879 (-)	WAACCA	S000408
GATABOX	888 (+)	GATA	S000039
GT1CONSENSUS	888 (+)	GRWAAW	S000198
IBOXCORE	888 (+)	GATAA	S000199
MARARS	890 (-)	WTTTATRTTTW	S000064
ROOTMOTIFTAPOX1	892 (-)	ATATT	S000098
SEF4MOTIFGM7S	896 (-)	RTTTTTR	S000103
TATABOX5	899 (-)	TTATTT	S000203
POLASIG1	900 (+)	AATAAA	S000080
CACTFTPPCA1	913 (-)	YACT	S000449
GTGANTG10	914 (+)	GTGA	S000378
DOFCOREZM	918 (+)	AAAG	S000265
NTBBF1ARROLB	923 (-)	ACTTTA	S000273
TAAAGSTKST1	923 (+)	TAAAG	S000387
DOFCOREZM	924 (+)	AAAG	S000265
CACTFTPPCA1	935 (+)	YACT	S000449
ARR1AT	944 (+)	NGATT	S000454
TATABOX4	947 (-)	TATATAA	S000111
CACTFTPPCA1	957 (-)	YACT	S000449
DOFCOREZM	971 (-)	AAAG	S000265
TAAAGSTKST1	971 (-)	TAAAG	S000387
POLASIG1	983 (-)	AATAAA	S000080
CAATBOX1	986 (-)	CAAT	S000028
WBBOXPCWRKY1	994 (+)	TTTGACY	S000310
WBOXATNPR1	995 (+)	TTGAC	S000390
WBOXHVISO1	996 (+)	TGACT	S000442
WRKY71OS	996 (+)	TGAC	S000447
WBOXNTERF3	996 (+)	TGACY	S000457
CACTFTPPCA1	1000 (+)	YACT	S000449
DOFCOREZM	1002 (-)	AAAG	S000265
MYBST1	1009 (+)	GGATA	S000180
GATABOX	1010 (+)	GATA	S000039
GATABOX	1016 (-)	GATA	S000039
NODCON1GM	1017 (-)	AAAGAT	S000461
OSE1ROOTNODULE	1017 (-)	AAAGAT	S000467
DOFCOREZM	1019 (-)	AAAG	S000265
CARGCW8GAT	1019 (-)	CWWWWWWWWG	S000431
CARGCW8GAT	1019 (+)	CWWWWWWWWG	S000431
POLASIG1	1022 (-)	AATAAA	S000080
CAATBOX1	1025 (-)	CAAT	S000028
CACTFTPPCA1	1032 (-)	YACT	S000449
POLASIG1	1036 (-)	AATAAA	S000080
TATABOX5	1037 (+)	TTATTT	S000203
RAV1AAT	1043 (-)	CAACA	S000314
SEF4MOTIFGM7S	1060 (+)	RTTTTTR	S000103
SEF1MOTIF	1063 (-)	ATATTTAWW	S000006
TATABOXOSPAL	1064 (-)	TATTTAA	S000400
ROOTMOTIFTAPOX1	1067 (-)	ATATT	S000098
GATABOX	1069 (-)	GATA	S000039
GATABOX	1075 (-)	GATA	S000039
ERELEE4	1081 (-)	AWTTCAAA	S000037
TATABOX5	1085 (-)	TTATTT	S000203
POLASIG1	1086 (+)	AATAAA	S000080
TATCCAOSAMY	1095 (-)	TATCCA	S000403
MYBST1	1096 (+)	GGATA	S000180
GATABOX	1097 (+)	GATA	S000039
GT1CONSENSUS	1106 (+)	GRWAAW	S000198
CACTFTPPCA1	1115 (+)	YACT	S000449
CCAATBOX1	1122 (+)	CCAAT	S000030
LEAFYATAG	1122 (+)	CCAATGT	S000432
CAATBOX1	1123 (+)	CAAT	S000028
TATAPVTRNALEU	1130 (+)	TTTATATA	S000340
TATABOX4	1131 (-)	TATATAA	S000111
GATABOX	1136 (-)	GATA	S000039
MYBST1	1136 (-)	GGATA	S000180
TATCCAOSAMY	1136 (+)	TATCCA	S000403
CCAATBOX1	1139 (+)	CCAAT	S000030
CAATBOX1	1140 (+)	CAAT	S000028
ATHB6COREAT	1140 (+)	CAATTATTA	S000399
POLASIG3	1142 (-)	AATAAT	S000088
ARR1AT	1152 (-)	NGATT	S000454
DPBFCOREDCDC3	1160 (+)	ACACNNG	S000292
EBOXBNNAPA	1161 (-)	CANNTG	S000144
MYCCONSENSUSAT	1161 (-)	CANNTG	S000407
MYCATERD1	1161 (-)	CATGTG	S000413
EBOXBNNAPA	1161 (+)	CANNTG	S000144
MYCATRD22	1161 (+)	CACATG	S000174
MYCCONSENSUSAT	1161 (+)	CANNTG	S000407
TATAPVTRNALEU	1168 (-)	TTTATATA	S000340
TATABOX4	1168 (+)	TATATAA	S000111
GT1CONSENSUS	1180 (-)	GRWAAW	S000198

Factor or Site Name	Loc. (Str.)	Signal Sequence	SITE #	Factor or Site Name	Loc. (Str.)	Signal Sequence	SITE #
IBOXCORE	1181 (-)	GATAA	S000199	MARTBOX	1377 (-)	TTWTWTTWTT	S000067
GATABOX	1182 (-)	GATA	S000039	POLASIG3	1377 (+)	AATAAT	S000088
POLASIG1	1191 (-)	AATAAA	S000080	ROOTMOTIFTAPOX1	1380 (-)	ATATT	S000098
TATABOX3	1193 (+)	TATTAAT	S000110	POLASIG3	1385 (+)	AATAAT	S000088
TATABOX3	1194 (-)	TATTAAT	S000110	POLASIG3	1388 (+)	AATAAT	S000088
ROOTMOTIFTAPOX1	1197 (-)	ATATT	S000098	POLASIG3	1391 (+)	AATAAT	S000088
GATABOX	1201 (-)	GATA	S000039	POLASIG3	1394 (+)	AATAAT	S000088
EBOXBNNAPA	1204 (-)	CANNTG	S000144	POLASIG3	1397 (+)	AATAAT	S000088
MYCCONSENSUSAT	1204 (-)	CANNTG	S000407	MARTBOX	1400 (-)	TTWTWTTWTT	S000067
EBOXBNNAPA	1204 (+)	CANNTG	S000144	POLASIG3	1400 (+)	AATAAT	S000088
MYCCONSENSUSAT	1204 (+)	CANNTG	S000407	ROOTMOTIFTAPOX1	1403 (-)	ATATT	S000098
AMYBOX1	1206 (-)	TAACARA	S000020	TATABOX2	1405 (+)	TATAAAT	S000109
MYBGAHV	1206 (-)	TAACAAA	S000181	TATABOX5	1408 (-)	TTATTT	S000203
GAREAT	1206 (-)	TAACAAR	S000439	POLASIG3	1409 (+)	AATAAT	S000088
CAATBOX1	1213 (-)	CAAT	S000028	TATABOX3	1413 (-)	TATTAAT	S000110
NAPINMOTIFBN	1222 (-)	TACACAT	S000070	POLASIG3	1416 (+)	AATAAT	S000088
GATABOX	1227 (-)	GATA	S000039	POLASIG3	1419 (+)	AATAAT	S000088
CACTFTPPCA1	1235 (-)	YACT	S000449	POLASIG3	1422 (+)	AATAAT	S000088
GTGANTG10	1238 (+)	GTGA	S000378	POLASIG3	1425 (+)	AATAAT	S000088
DOFCOREZM	1241 (+)	AAAG	S000265	SEF4MOTIFGM7S	1437 (+)	RTTTTTR	S000103
CACTFTPPCA1	1255 (+)	YACT	S000449	BIHD1OS	1447 (-)	TGTCA	S000498
T/GBOXATPIN2	1262 (+)	AACGTG	S000458	WRKY71OS	1447 (+)	TGAC	S000447
ABRERATCAL	1262 (+)	MACGYGB	S000507	EECCRCAH1	1452 (+)	GANTTNC	S000494
ACGTATERD1	1263 (-)	ACGT	S000415	GTGANTG10	1465 (-)	GTGA	S000378
ABRELATERD1	1263 (+)	ACGTG	S000414	CAATBOX1	1468 (+)	CAAT	S000028
ACGTATERD1	1263 (+)	ACGT	S000415	ARR1AT	1485 (+)	NGATT	S000454
MYB1AT	1266 (-)	WAACCA	S000408	EECCRCAH1	1493 (-)	GANTTNC	S000494
IBOXCORE	1269 (-)	GATAA	S000199	CAATBOX1	1494 (+)	CAAT	S000028
GATABOX	1270 (-)	GATA	S000039	DOFCOREZM	1499 (-)	AAAG	S000265
RAV1AAT	1276 (-)	CAACA	S000314	RAV1AAT	1508 (+)	CAACA	S000314
DOFCOREZM	1286 (-)	AAAG	S000265	TATABOX5	1512 (-)	TTATTT	S000203
RAV1AAT	1291 (-)	CAACA	S000314	POLASIG1	1513 (+)	AATAAA	S000080
SEF3MOTIFGM	1294 (-)	AACCCA	S000115	GATABOX	1530 (+)	GATA	S000039
TATABOX5	1298 (+)	TTATTT	S000203	GT1CONSENSUS	1530 (+)	GRWAAW	S000198
POLASIG1	1301 (-)	AATAAA	S000080	IBOXCORE	1530 (+)	GATAA	S000199
MARTBOX	1302 (+)	TTWTWTTWTT	S000067	CACTFTPPCA1	1546 (-)	YACT	S000449
TATABOX5	1302 (+)	TTATTT	S000203	TATABOX5	1549 (-)	TTATTT	S000203
AUXREPSIAA4	1314 (-)	KGTCCCAT	S000026	POLASIG1	1550 (+)	AATAAA	S000080
ARR1AT	1322 (+)	NGATT	S000454	CAATBOX1	1559 (-)	CAAT	S000028
CAATBOX1	1324 (-)	CAAT	S000028	CEREGLUBOX2PSLEGA	1561 (+)	TGAAAACT	S000033
ROOTMOTIFTAPOX1	1343 (-)	ATATT	S000098	SEF4MOTIFGM7S	1568 (-)	RTTTTTR	S000103
NODCON2GM	1352 (-)	CTCTT	S000462	TATABOX2	1573 (-)	TATAAAT	S000109
OSE2ROOTNODULE	1352 (-)	CTCTT	S000468	POLASIG2	1579 (+)	AATTAAA	S000081
ARR1AT	1355 (+)	NGATT	S000454	GT1CONSENSUS	1585 (-)	GRWAAW	S000198
TATABOX3	1357 (-)	TATTAAT	S000110	ROOTMOTIFTAPOX1	1591 (+)	ATATT	S000098
POLASIG3	1360 (+)	AATAAT	S000088	GT1CONSENSUS	1593 (-)	GRWAAW	S000198
MARTBOX	1363 (-)	TTWTWTTWTT	S000067	POLASIG2	1605 (+)	AATTAAA	S000081
POLASIG3	1363 (+)	AATAAT	S000088	TATABOXOSPAL	1615 (-)	TATTTAA	S000400
ROOTMOTIFTAPOX1	1366 (-)	ATATT	S000098	TATABOX5	1617 (-)	TTATTT	S000203
POLASIG3	1371 (+)	AATAAT	S000088	POLASIG3	1618 (+)	AATAAT	S000088
POLASIG3	1374 (+)	AATAAT	S000088	ROOTMOTIFTAPOX1	1621 (-)	ATATT	S000098

Factor or Site Name	Loc.(Str.)	Signal Sequence	SITE #	Factor or Site Name	Loc.(Str.)	Signal Sequence	SITE #
GATABOX	1625 (-)	GATA	S000039	GATABOX	1853 (+)	GATA	S000039
ANAERO3CONSENSUS	1627 (+)	TCATCAC	S000479	ROOTMOTIFTAPOX1	1860 (-)	ATATT	S000098
GTGANTG10	1630 (-)	GTGA	S000378	ROOTMOTIFTAPOX1	1863 (+)	ATATT	S000098
CACTFTPPCA1	1631 (+)	YACT	S000449	TATABOX3	1864 (+)	TATTAAT	S000110
NTBBF1ARROLB	1632 (+)	ACTTTA	S000273	TATABOX3	1865 (-)	TATTAAT	S000110
DOFCOREZM	1633 (-)	AAAG	S000265	POLASIG3	1880 (-)	AATAAT	S000088
TAAAGSTKST1	1633 (-)	TAAAG	S000387	TATABOX5	1881 (+)	TTATTT	S000203
TATABOXOSPAL	1635 (-)	TATTTAA	S000400	TATABOXOSPAL	1882 (+)	TATTTAA	S000400
TATABOX5	1637 (-)	TTATTT	S000203	POLASIG2	1884 (-)	AATTAAA	S000081
POLASIG3	1638 (+)	AATAAT	S000088	POLASIG3	1888 (-)	AATAAT	S000088
TATABOX3	1653 (+)	TATTAAT	S000110	TATABOX5	1889 (+)	TTATTT	S000203
TATABOX3	1654 (-)	TATTAAT	S000110	TATABOX2	1891 (-)	TATAAAT	S000109
ROOTMOTIFTAPOX1	1657 (-)	ATATT	S000098	MARTBOX	1897 (-)	TTWTWTTWTT	S000067
ROOTMOTIFTAPOX1	1658 (+)	ATATT	S000098	POLASIG1	1897 (+)	AATAAA	S000080
POLLEN1LELAT52	1664 (+)	AGAAA	S000245	TATABOX5	1901 (-)	TTATTT	S000203
EECCRCAH1	1665 (-)	GANTTNC	S000494	POLASIG3	1902 (+)	AATAAT	S000088
ERELEE4	1667 (+)	AWTTCAAA	S000037	ARR1AT	1905 (-)	NGATT	S000454
ROOTMOTIFTAPOX1	1673 (-)	ATATT	S000098	EECCRCAH1	1910 (-)	GANTTNC	S000494
WRKY71OS	1677 (+)	TGAC	S000447	GT1CONSENSUS	1910 (+)	GRWAAW	S000198
WBOXNTERF3	1677 (+)	TGACY	S000457	ARR1AT	1913 (-)	NGATT	S000454
CACTFTPPCA1	1681 (+)	YACT	S000449	CCAATBOX1	1919 (+)	CCAAT	S000030
DOFCOREZM	1683 (-)	AAAG	S000265	CAATBOX1	1920 (+)	CAAT	S000028
LECPLEACS2	1688 (-)	TAAAATAT	S000465	ARR1AT	1925 (+)	NGATT	S000454
ROOTMOTIFTAPOX1	1688 (+)	ATATT	S000098	POLASIG3	1927 (-)	AATAAT	S000088
POLASIG2	1692 (-)	AATTAAA	S000081	L1BOXATPDF1	1932 (-)	TAAATGYA	S000386
GT1CONSENSUS	1699 (-)	GRWAAW	S000198	SEF4MOTIFGM7S	1938 (-)	RTTTTTR	S000103
GT1GMSCAM4	1699 (-)	GAAAAA	S000453	CAATBOX1	1943 (-)	CAAT	S000028
INRNTPSADB	1702 (+)	YTCANTYY	S000395	CAATBOX1	1949 (+)	CAAT	S000028
GTGANTG10	1720 (-)	GTGA	S000378	NTBBF1ARROLB	1967 (-)	ACTTTA	S000273
CAATBOX1	1733 (-)	CAAT	S000028	TAAAGSTKST1	1967 (+)	TAAAG	S000387
CCAATBOX1	1733 (-)	CCAAT	S000030	INRNTPSADB	1968 (-)	YTCANTYY	S000395
GTGANTG10	1739 (-)	GTGA	S000378	DOFCOREZM	1968 (+)	AAAG	S000265
CACTFTPPCA1	1740 (+)	YACT	S000449	CACTFTPPCA1	1970 (-)	YACT	S000449
ANAERO1CONSENSUS	1751 (-)	AAACAAA	S000477	GTGANTG10	1971 (+)	GTGA	S000378
POLASIG1	1756 (-)	AATAAA	S000080	GT1CONSENSUS	1973 (+)	GRWAAW	S000198
CPBCSPOR	1758 (+)	TATTAG	S000491	GT1GMSCAM4	1973 (+)	GAAAAA	S000453
ARR1AT	1773 (+)	NGATT	S000454	DOFCOREZM	1978 (+)	AAAG	S000265
BOXIINTPATPB	1776 (-)	ATAGAA	S000296	GATABOX	1984 (+)	GATA	S000039
ROOTMOTIFTAPOX1	1782 (+)	ATATT	S000098	SEF1MOTIF	1985 (+)	ATATTTAWW	S000006
MARTBOX	1799 (+)	TTWTWTTWTT	S000067	ROOTMOTIFTAPOX1	1985 (+)	ATATT	S000098
MARTBOX	1800 (+)	TTWTWTTWTT	S000067	POLASIG1	1988 (-)	AATAAA	S000080
GT1CONSENSUS	1805 (-)	GRWAAW	S000198	CACTFTPPCA1	1995 (+)	YACT	S000449
GT1GMSCAM4	1805 (-)	GAAAAA	S000453	ROOTMOTIFTAPOX1	1999 (+)	ATATT	S000098
POLLEN1LELAT52	1807 (-)	AGAAA	S000245	CACTFTPPCA1	2003 (+)	YACT	S000449
TATAPVTRNALEU	1819 (-)	TTTATATA	S000340	NTBBF1ARROLB	2004 (+)	ACTTTA	S000273
TATABOX4	1819 (+)	TATATAA	S000111	DOFCOREZM	2005 (-)	AAAG	S000265
TATABOX2	1827 (-)	TATAAAT	S000109	TAAAGSTKST1	2005 (-)	TAAAG	S000387
TATAPVTRNALEU	1828 (+)	TTTATATA	S000340	POLASIG2	2006 (-)	AATTAAA	S000081
TATABOX4	1829 (-)	TATATAA	S000111	CAATBOX1	2010 (-)	CAAT	S000028
CARGCW8GAT	1844 (-)	CWWWWWWWWG	S000431	ARR1AT	2012 (+)	NGATT	S000454
CARGCW8GAT	1844 (+)	CWWWWWWWWG	S000431	CAATBOX1	2014 (-)	CAAT	S000028

Factor or Site Name	Loc.(Str.)	Signal Sequence	SITE #	Factor or Site Name	Loc.(Str.)	Signal Sequence	SITE #
CCAATBOX1	2014 (-)	CCAAT	S000030	EBOXBNNAPA	2212 (-)	CANNTG	S000144
GT1CONSENSUS	2017 (+)	GRWAAW	S000198	CATATGGMSAUR	2212 (-)	CATATG	S000370
CACTFTPPCA1	2022 (+)	YACT	S000449	MYCCONSENSUSAT	2212 (-)	CANNTG	S000407
TAAAGSTKST1	2030 (+)	TAAAG	S000387	EBOXBNNAPA	2212 (+)	CANNTG	S000144
DOFCOREZM	2031 (+)	AAAG	S000265	CATATGGMSAUR	2212 (+)	CATATG	S000370
RAV1AAT	2036 (+)	CAACA	S000314	MYCCONSENSUSAT	2212 (+)	CANNTG	S000407
ROOTMOTIFTAPOX1	2040 (+)	ATATT	S000098	PREATPRODH	2221 (-)	ACTCAT	S000450
CAATBOX1	2042 (-)	CAAT	S000028	GCN4OSGLUB1	2222 (+)	TGAGTCA	S000277
ARR1AT	2044 (+)	NGATT	S000454	WBOXHVISO1	2224 (-)	TGACT	S000442
ANAERO1CONSENSUS	2053 (+)	AAACAAA	S000477	WBOXNTERF3	2224 (-)	TGACY	S000457
TAAAGSTKST1	2062 (+)	TAAAG	S000387	WBOXATNPR1	2225 (-)	TTGAC	S000390
DOFCOREZM	2063 (+)	AAAG	S000265	WRKY71OS	2225 (-)	TGAC	S000447
RAV1AAT	2068 (+)	CAACA	S000314	CARGCW8GAT	2227 (-)	CWWWWWWWWG	S000431
TATABOX5	2077 (-)	TTATTT	S000203	CAATBOX1	2227 (+)	CAAT	S000028
SEF1MOTIF	2078 (-)	ATATTTAWW	S000006	CARGCW8GAT	2227 (+)	CWWWWWWWWG	S000431
POLASIG1	2078 (+)	AATAAA	S000080	ROOTMOTIFTAPOX1	2228 (-)	ATATT	S000098
ROOTMOTIFTAPOX1	2082 (-)	ATATT	S000098	ROOTMOTIFTAPOX1	2229 (+)	ATATT	S000098
TATABOX4	2084 (+)	TATATAA	S000111	CACTFTPPCA1	2239 (-)	YACT	S000449
POLASIG3	2090 (-)	AATAAT	S000088	ROOTMOTIFTAPOX1	2247 (-)	ATATT	S000098
TATABOX5	2091 (+)	TTATTT	S000203	ROOTMOTIFTAPOX1	2248 (+)	ATATT	S000098
POLASIG1	2094 (-)	AATAAA	S000080	SEF1MOTIF	2256 (-)	ATATTTAWW	S000006
TATABOX5	2095 (+)	TTATTT	S000203	TATABOXOSPAL	2257 (-)	TATTTAA	S000400
-300ELEMENT	2097 (-)	TGHAAARK	S000122	ROOTMOTIFTAPOX1	2260 (-)	ATATT	S000098
GT1CONSENSUS	2098 (-)	GRWAAW	S000198	LECPLEACS2	2261 (-)	TAAAATAT	S000465
GT1GMSCAM4	2098 (-)	GAAAAA	S000453	ROOTMOTIFTAPOX1	2261 (+)	ATATT	S000098
INRNTPSADB	2101 (+)	YTCANTYY	S000395	CACTFTPPCA1	2279 (-)	YACT	S000449
CARGCW8GAT	2103 (-)	CWWWWWWWWG	S000431	DOFCOREZM	2286 (-)	AAAG	S000265
CAATBOX1	2103 (+)	CAAT	S000028	POLLEN1LELAT52	2300 (+)	AGAAA	S000245
CARGCW8GAT	2103 (+)	CWWWWWWWWG	S000431	CACTFTPPCA1	2317 (+)	YACT	S000449
GTGANTG10	2112 (+)	GTGA	S000378	-300ELEMENT	2318 (-)	TGHAAARK	S000122
MYBST1	2116 (+)	GGATA	S000180	DOFCOREZM	2319 (-)	AAAG	S000265
GATABOX	2117 (+)	GATA	S000039	MYB1AT	2325 (+)	WAACCA	S000408
CACTFTPPCA1	2122 (-)	YACT	S000449	REALPHALGLHCB21	2326 (+)	AACCAA	S000362
GTGANTG10	2123 (+)	GTGA	S000378	ARR1AT	2331 (-)	NGATT	S000454
GATABOX	2129 (+)	GATA	S000039	CARGCW8GAT	2334 (-)	CWWWWWWWWG	S000431
POLASIG2	2134 (+)	AATTAAA	S000081	CARGCW8GAT	2334 (+)	CWWWWWWWWG	S000431
ARR1AT	2139 (-)	NGATT	S000454	CAATBOX1	2340 (-)	CAAT	S000028
CAATBOX1	2149 (-)	CAAT	S000028	CAATBOX1	2345 (-)	CAAT	S000028
WBOXATNPR1	2150 (+)	TTGAC	S000390	ARR1AT	2352 (+)	NGATT	S000454
BIHD1OS	2151 (-)	TGTCA	S000498	POLASIG2	2370 (-)	AATTAAA	S000081
WRKY71OS	2151 (+)	TGAC	S000447	GTGANTG10	2379 (-)	GTGA	S000378
POLASIG1	2163 (+)	AATAAA	S000080	CACTFTPPCA1	2380 (+)	YACT	S000449
INRNTPSADB	2166 (-)	YTCANTYY	S000395	INRNTPSADB	2385 (-)	YTCANTYY	S000395
CAATBOX1	2168 (-)	CAAT	S000028	CAATBOX1	2387 (-)	CAAT	S000028
GT1CONSENSUS	2189 (-)	GRWAAW	S000198	INRNTPSADB	2390 (-)	YTCANTYY	S000395
POLLEN1LELAT52	2191 (-)	AGAAA	S000245	CAATBOX1	2392 (-)	CAAT	S000028
DOFCOREZM	2198 (+)	AAAG	S000265	MYBCOREATCYCB1	2397 (+)	AACGG	S000502
CACTFTPPCA1	2200 (-)	YACT	S000449	CURECORECR	2401 (-)	GTAC	S000493
SEF1MOTIF	2203 (+)	ATATTTAWW	S000006	CURECORECR	2401 (+)	GTAC	S000493
ROOTMOTIFTAPOX1	2203 (+)	ATATT	S000098	CAATBOX1	2407 (+)	CAAT	S000028
POLASIG1	2206 (-)	AATAAA	S000080	GATABOX	2421 (-)	GATA	S000039

Factor or Site Name	Loc.(Str.)	Signal Sequence	SITE #	Factor or Site Name	Loc.(Str.)	Signal Sequence	SITE #
ARR1AT	2426 (-)	NGATT	S000454	CACTFTPPCA1	2671 (+)	YACT	S000449
SEF4MOTIFGM7S	2429 (-)	RTTTTTR	S000103	TATABOX4	2674 (+)	TATATAA	S000111
INRNTPSADB	2431 (-)	YTCANTYY	S000395	ROOTMOTIFTAPOX1	2679 (-)	ATATT	S000098
ARR1AT	2437 (-)	NGATT	S000454	CACTFTPPCA1	2683 (+)	YACT	S000449
RHERPATEXPA7	2439 (-)	KCACGW	S000512	WRKY71OS	2695 (+)	TGAC	S000447
EBOXBNNAPA	2444 (-)	CANNTG	S000144	WBOXNTERF3	2695 (+)	TGACY	S000457
CATATGGMSAUR	2444 (-)	CATATG	S000370	CCAATBOX1	2706 (+)	CCAAT	S000030
MYCCONSENSUSAT	2444 (-)	CANNTG	S000407	CAATBOX1	2707 (+)	CAAT	S000028
EBOXBNNAPA	2444 (+)	CANNTG	S000144	ARR1AT	2727 (-)	NGATT	S000454
CATATGGMSAUR	2444 (+)	CATATG	S000370	CAATBOX1	2734 (+)	CAAT	S000028
MYCCONSENSUSAT	2444 (+)	CANNTG	S000407	ARR1AT	2735 (-)	NGATT	S000454
RAV1AAT	2448 (-)	CAACA	S000314	GTGANTG10	2737 (-)	GTGA	S000378
ARR1AT	2457 (-)	NGATT	S000454	CACTFTPPCA1	2743 (-)	YACT	S000449
SEF1MOTIF	2465 (-)	ATATTTAWW	S000006	DOFCOREZM	2750 (+)	AAAG	S000265
TATABOX2	2465 (+)	TATAAAT	S000109	NODCON2GM	2751 (-)	CTCTT	S000462
ROOTMOTIFTAPOX1	2469 (-)	ATATT	S000098	OSE2ROOTNODULE	2751 (-)	CTCTT	S000468
ROOTMOTIFTAPOX1	2472 (+)	ATATT	S000098	CACTFTPPCA1	2757 (-)	YACT	S000449
TATABOX4	2475 (-)	TATATAA	S000111	-10PEHVPSBD	2760 (-)	TATTCT	S000392
ROOTMOTIFTAPOX1	2479 (+)	ATATT	S000098	T/GBOXATPIN2	2777 (+)	AACGTG	S000458
MYB1AT	2488 (-)	WAACCA	S000408	ABRERATCAL	2777 (+)	MACGYGB	S000507
TATABOX4	2491 (-)	TATATAA	S000111	ACGTATERD1	2778 (-)	ACGT	S000415
TATABOX4	2500 (+)	TATATAA	S000111	ABRELATERD1	2778 (+)	ACGTG	S000414
ROOTMOTIFTAPOX1	2505 (-)	ATATT	S000098	ACGTATERD1	2778 (+)	ACGT	S000415
ROOTMOTIFTAPOX1	2538 (+)	ATATT	S000098	MYBCORE	2786 (-)	CNGTTR	S000176
ROOTMOTIFTAPOX1	2545 (-)	ATATT	S000098	CACTFTPPCA1	2805 (+)	YACT	S000449
ROOTMOTIFTAPOX1	2550 (+)	ATATT	S000098	DOFCOREZM	2811 (+)	AAAG	S000265
ARR1AT	2558 (+)	NGATT	S000454	NODCON1GM	2811 (+)	AAAGAT	S000461
TATABOX4	2568 (-)	TATATAA	S000111	OSE1ROOTNODULE	2811 (+)	AAAGAT	S000467
TATABOX4	2569 (+)	TATATAA	S000111	GATABOX	2814 (+)	GATA	S000039
ROOTMOTIFTAPOX1	2579 (-)	ATATT	S000098	IBOX	2814 (+)	GATAAG	S000124
ROOTMOTIFTAPOX1	2580 (+)	ATATT	S000098	IBOXCORE	2814 (+)	GATAA	S000199
GT1CONSENSUS	2582 (-)	GRWAAW	S000198	IBOXCORENT	2814 (+)	GATAAGR	S000424
IBOXCORE	2583 (-)	GATAA	S000199	NODCON2GM	2817 (-)	CTCTT	S000462
GATABOX	2584 (-)	GATA	S000039	OSE2ROOTNODULE	2817 (-)	CTCTT	S000468
GATABOX	2588 (-)	GATA	S000039	POLLEN1LELAT52	2820 (+)	AGAAA	S000245
BOXIINTPATPB	2596 (-)	ATAGAA	S000296	GT1CONSENSUS	2821 (+)	GRWAAW	S000198
ARR1AT	2611 (+)	NGATT	S000454	GT1GMSCAM4	2821 (+)	GAAAAA	S000453
ROOTMOTIFTAPOX1	2625 (-)	ATATT	S000098	DOFCOREZM	2824 (+)	AAAG	S000265
ROOTMOTIFTAPOX1	2628 (+)	ATATT	S000098	CACTFTPPCA1	2831 (+)	YACT	S000449
CPBCSPOR	2629 (+)	TATTAG	S000491	DOFCOREZM	2842 (+)	AAAG	S000265
GTGANTG10	2636 (-)	GTGA	S000378	NODCON2GM	2843 (-)	CTCTT	S000462
CACTFTPPCA1	2637 (+)	YACT	S000449	OSE2ROOTNODULE	2843 (-)	CTCTT	S000468
NTBBF1ARROLB	2638 (+)	ACTTTA	S000273	CACTFTPPCA1	2846 (-)	YACT	S000449
DOFCOREZM	2639 (-)	AAAG	S000265	GATABOX	2851 (+)	GATA	S000039
TAAAGSTKST1	2639 (-)	TAAAG	S000387	CAATBOX1	2855 (+)	CAAT	S000028
CACTFTPPCA1	2647 (+)	YACT	S000449	SEF4MOTIFGM7S	2862 (-)	RTTTTTR	S000103
HDZIP2ATATHB2	2651 (-)	TAATMATTA	S000373	DOFCOREZM	2868 (-)	AAAG	S000265
POLASIG3	2653 (-)	AATAAT	S000088	TELOBOXATEEF1AA1	2873 (+)	AAACCCTAA	S000308
CPBCSPOR	2655 (+)	TATTAG	S000491	UP2ATMSD	2873 (+)	AAACCCTA	S000472
CACTFTPPCA1	2659 (-)	YACT	S000449	CCAATBOX1	2884 (+)	CCAAT	S000030
BOXIINTPATPB	2663 (-)	ATAGAA	S000296	LEAFYATAG	2884 (+)	CCAATGT	S000432

Factor or Site Name	Loc.(Str.)	Signal Sequence	SITE #
CAATBOX1	2885 (+)	CAAT	S000028
GT1CONSENSUS	2892 (-)	GRWAAW	S000198
GTGANTG10	2905 (-)	GTGA	S000378
CAREOSREP1	2908 (+)	CAACTC	S000421
CPBCSPOR	2913 (-)	TATTAG	S000491
ROOTMOTIFTAPOX1	2915 (-)	ATATT	S000098
LECPLEACS2	2916 (-)	TAAAATAT	S000465
ROOTMOTIFTAPOX1	2916 (+)	ATATT	S000098
GT1CONSENSUS	2920 (-)	GRWAAW	S000198
IBOXCORE	2921 (-)	GATAA	S000199
SREATMSD	2921 (+)	TTATCC	S000470
GATABOX	2922 (-)	GATA	S000039
MYBST1	2922 (-)	GGATA	S000180
ARR1AT	2928 (-)	NGATT	S000454
E2FCONSENSUS	2940 (-)	WTTSSCSS	S000476
HSELIKENTACIDICPR1	2941 (-)	CNNGAANNNTTCNNG	S000056
HSELIKENTACIDICPR1	2941 (+)	CNNGAANNNTTCNNG	S000056
DOFCOREZM	2948 (-)	AAAG	S000265
WBOXNTERF3	2959 (-)	TGACY	S000457
WRKY71OS	2960 (-)	TGAC	S000447
GTGANTG10	2961 (-)	GTGA	S000378
CAATBOX1	2964 (+)	CAAT	S000028
GT1CONSENSUS	2978 (+)	GRWAAW	S000198
GT1GMSCAM4	2978 (+)	GAAAAA	S000453
GATABOX	2986 (-)	GATA	S000039
MYBST1	2986 (-)	GGATA	S000180
TATCCAOSAMY	2986 (+)	TATCCA	S000403
SORLIP1AT	3000 (+)	GCCAC	S000482
//			

Supplementary Table 3:

The top 10 threading templates used by I-TASSER in predicting the 3D structures of Uncharacterized protein LOC110648447 isoforms

I-TASSER modeling starts from the structure templates identified by LOMETS from the PDB library. LOMETS is a meta-server threading approach containing multiple threading programs, where each threading program can generate tens of thousands of template alignments. I-TASSER only uses the templates of the highest significance in the threading alignments, the significance of which are measured by the Z-score, i.e. the difference between the raw and average scores in the unit of standard deviation. The templates in this section are the 10 best templates selected from the LOMETS threading programs. Usually, one template of the highest Z-score is selected from each threading program, where the threading programs are sorted by the average performance in the large-scale benchmark test experiments.

List of top 10 template of each isoform

Isoform	Tools	PDB hit	Name	Iden1	Iden2	Coverage	Normalized Z-score
Isoform X1	1	4igvA	Crystal structure of kirola (Act d 11)	0.18	0.19	0.93	2.29
	2	6ka2A	Crystal structure of a Thebaine synthase from *Papaver somniferum* in complex with TBN	0.11	0.12	0.96	3.02
	3	7mlcA	PYL10 bound to the ABA pan-antagonist 4a	0.13	0.19	0.97	1.86
	4	6ka2A	Crystal structure of a Thebaine synthase from *Papaver somniferum* in complex with TBN	0.13	0.12	0.92	1.53
	5	1vjh	Crystal structure of gene product of At1g24000 from *Arabidopsis thaliana*	0.19	0.20	0.78	1.71
	6	4igvA	Crystal structure of kirola (Act d 11)	0.19	0.19	0.93	2.68
	7	1vjh	Crystal structure of gene product of At1g24000 from *Arabidopsis thaliana*	0.15	0.20	0.78	2.85
	8	4jhgA	Crystal structure of *Medicago truncatula* Nodulin 13 (MtN13) in complex with trans-zeatin	0.14	0.17	0.98	1.72
	9	4m9bA	Crystal structure of Apo Ara h 8	0.18	0.20	0.97	1.88
	10	7mlcA	PYL10 bound to the ABA pan-antagonist 4a	0.13	0.19	0.94	1.25
Isoform X2	1	4rehA	Crystal structure of ginseng major latex-like protein 151 (GLP) from *Panax ginseng* (crystal-1)	0.16	0.20	0.94	2.31
	2	4rehA	Crystal structure of ginseng major latex-like protein 151 (GLP) from *Panax ginseng* (crystal-1)	0.16	0.20	0.93	3.09
	3	7mlcA	PYL10 bound to the ABA pan-antagonist 4a	0.13	0.20	0.95	1.89
	4	1icx	Crystal structure of pathogenesis-related protein LlPR10.1A from yellow lupine	0.10	0.15	0.93	1.66
	5	1vjh	Crystal structure of gene product of At1g24000 from *Arabidopsis thaliana*	0.16	0.20	0.75	1.77
	6	4rehA	Crystal structure of ginseng major latex-like protein 151 (GLP) from *Panax ginseng* (crystal-1)	0.15	0.20	0.93	2.61
	7	1vjh	Crystal structure of gene product of At1g24000 from *Arabidopsis thaliana*	0.13	0.20	0.76	2.78
	8	4jhgA	Crystal structure of *Medicago truncatula* Nodulin 13 (MtN13) in complex with trans-zeatin	0.14	0.16	0.98	1.84
	9	7mlcA	PYL10 bound to the ABA pan-antagonist 4a	0.10	0.20	0.95	1.20
	10	2i9yA	Solution structure of *Arabidopsis thaliana* protein At1g70830, a member of the major latex protein family	0.17	0.20	0.95	3.22
Isoform X3	1	4rehA	Crystal structure of ginseng major latex-like protein 151 (GLP) from *Panax ginseng* (crystal-1)	0.16	0.19	0.93	2.30
	2	4igvA	Crystal structure of kirola (Act d 11)	0.16	0.16	0.93	3.03
	3	7mlcA	PYL10 bound to the ABA pan-antagonist 4a	0.13	0.20	0.95	1.75
	4	1icx	Crystal structure of pathogenesis-related protein LlPR10.1A from yellow lupine	0.09	0.19	0.93	1.64
	5	1vjh	Crystal structure of gene product of At1g24000 from *Arabidopsis thaliana*	0.15	0.20	0.75	1.81
	6	4rehA	Crystal structure of ginseng major latex-like protein 151 (GLP) from *Panax ginseng* (crystal-1)	0.15	0.19	0.93	2.76
	7	1vjh	Crystal structure of gene product of At1g24000 from *Arabidopsis thaliana*	0.13	0.20	0.77	2.76
	8	4c9cA	Crystal structure of the strawberry pathogenesis-related 10 (PR-10) Fra a 1E protein (Form A)	0.16	0.20	0.97	1.72
	9	4m9bA	Crystal structure of Apo Ara h 8	0.16	0.19	0.96	1.95
	10	7mlcA	PYL10 bound to the ABA pan-antagonist 4a	0.12	0.20	0.93	1.21

The top 10 alignments reported above (in order of their ranking) are from the following threading programs: 1: MUSTER, 2: FFAS-3D, 3: SPARKS-X, 4: HHSEARCH2, 5: HHSEARCH, I 6: Neff-PPAS, 7: HHSEARCH, 8: pGenTHREADER, 9: wdPPAS, 10: PROSPECT2. Rank of templates represents the top ten threading templates used by I-TASSER. **Ident1** is the percentage sequence identity of the templates in the threading aligned region with the query sequence. **Ident2** is the percentage sequence identity of the whole template chains with query sequence. **Coverage** represents the coverage of the threading alignment and is equal to the number of aligned residues divided by the length of query protein. **Normalized Z-score** is the normalized Z-score of the threading alignments. Alignment with a Normalized Z-score >1 mean a good alignment and vice versa.

Supplementary Table 4:

The alignment of Uncharacterized protein LOC110648447 isoforms with the top 10 threading templates in supplementary table 3

	PDB hit	Alignment
Isoform X1		20 40 60 80 100 120 140
	Query	MAFVTATAGVQVLIKSLPDTFWTKIQDSANSFPEAAPNLYSSIAVSGTGVRKVEYGPDSQNIKNSSELITENSRGKLAYTVTDGDILQKYKVKTFQAEIIYPNTGKWVEWIWTYNYLPEDKTRILELDDEEIAVITVKTLAKLDSYIQNKLT
	1.4igvA	--DLSGKMVKQVEILSDGIVFYEIFRYRLYLISEMSPVNIQGVDLLEGSVIFFKYTIDGKTAKDIVEAIDE-ETKSVTFKIVEGDLMELYKTFIIIVQVDTKGEHNSVTWTFHYEKLKED----VEEPNTLMNFCIEITKDIETYHLK---
	2.6ka2A	SG-LVGKLSTELEVDCDAEKYYNMYKHGEDV-KKAVPHLCVDVKISGDPIKEWNVNIDGKTIRSVEETTHDDETKTLRHRVFEGDVMKDFKKFDTIMVVNPKPDGNGCVVTRSIEYEK--TNENSPTPFDYLQFGHQAIEDMNKYLRDS—
	3.7mlcA	---SQCSSTLVKHIKAPLHLVWSIVRRFDE-PQKYKPFISRCVVLEVGSVREVDLKSGLPATKTEVLEILDDNEHILGIRIVGGDHLKNY-SSTISLHSETIDGKTGTLAIESFVVDVPEGNTKEETCFFVEALIQCNLNSLADVTERLQA
	4.6ka2A	VSGLVGKLSTELEVDCDAEKYYNMYKHG-EDVKKAVPLC-VDVKIDPGCIKEWNVNIDGKT-IRSVEETTHDETKTLRHRVFEGDVMK--DFKKFDTIMVNPKDGCVVTRSIEYEKTNENSP----TPFDYLQFGHQAIEDMNKYLRD---
	5.1vjh	STL-KGALSVKFDVKCPADKFFSAFVEDT------------------NR-------PFEKNGKTEIEAV-DLVKKTT-I-QSGSEIQKYFKTLKGSIAVTPIGVGDGSHVVWTFHFEKVHKD--IDDPHSIIDESVKYFKKLDEAILNF—
	6.4igvA	---LSGKMVKQVEILSDGIVFYEIFRYRLYLISEMSPVNIQGVDLLEGSVIFFKYTIDGKTAKDIVEAIDEET-KSVTFKIVEGDLMELYKTFIIIVQVDTKGEHNSVTWTFHYEKLKED----VEEPNTLMNFCIEITKDIETYHLK---
	7.1vjh	ST-LKGALSVKFDVKCPADKFFSAFVEDT----------------NR----------PFEKNGKTEIEAVDLVKKTT-I-QSGSEIQKYFKTLKGSIAVTPIGVGDGSHVVWTFHFEKVHKD--IDDPHSIIDESVKYFKKLDEAILNF—
	8.4jhgA	MG--VITSESEYVSSLSAEKLYRGIVEDNIIYPKALPRFIEKAELEGDGIKKLTFVGDFGSTKQHIDMVDR-ENCAYTYSVYEGIALSPLEKIVFEFKLVPTPEEGIVKSTTKYYTKGDDIELSKDYLEAGIERFEGFTKAVESFLLANPD
	9.4m9bA	---GVFTFEDEITSTVPPAKLYNAMKDADSITPKIIDDVKSVEIVEGNGIKKLTIVEDGEFILHKVESIDE-ANYAYNYSVVGGVALPTAEKITFETKLVEGPNGGIGKLTLKYHTK-GDAKPDEEELKKGKAKGEGLFRAIEGYVLANPT
	10.7mlcA	S---QCSSTLVKHIKAPLHLVWSIVRRFDEP--QKYKPFISRCVVQGKKVREVDLKSGLPATKSTEVEILDDNEHILGIRIVGGDHRLKNYSSTISLHSETIDGKTGTLAIESFVVDVPEG----NTKEETCFFVEALIQCNLNSLAESME
Isoform X2		20 40 60 80 100 120 140
	Query	MAFVTATAGVQVLIKSQPDTFWTKIQDSANSFPQAAPSLYTSILISGTGVRKVEYGPDSQNIKTSSELITENSLGKLAYTVTAGDILQKYDVNNFQAVIIHPNSEKWVGWTWTYNYLPENKARALQLDAEIAVIAVKTLTELDNYIQNKST
	1.4rehA	-GL-TGKLICQTGIKSDGDVFHELFGTRPHHVPNITPANIGCDLHEGEFVVIWNYSIDGNMIAKEEIVAIDEEDKSVTFKVVEGHLFEEFKSIVFSVHVDTKGEDNLVTWSIDYEKLNE----SVKDPTSYLDFLLSVTRDIEAHHLP---
	2.4rehA	---LTGKLICQTGIKSDGDVFHELFGTRPHHVPNITPANIQGCDLEGEFVVIWNYSIDGNAMIAKEEVAIDEEDKSVTFKVVEGHLFEEFKSIVFSVHVDTKGEDNLVTWSIDYEKLNE----SVKDPTSYLDFLLSVTRDIEAHHLP---
	3.7mlcA	---SQCSSTLVKHIKAPLHLVWSIVRRFDE-PQKYKPF-ISRCVVQGKSVREVDLKSGLPATKTEVLEILDDNEHILGIRIVGGDHLKNY-SSTISLHSETIDGGTLAIESFVVDVPEG--NTKEETCFFVEALIQCNLNSLADVTERLQA
	4.1icx	---GIFAFENEQSSTVAPAKLYKATKDS-DEIPKVIEP-IQSVEINGGTIKKIIIHDGH--TSFVLHKLDADEANTYNYSIIGGEGLESLE--KISYESILPGPGSIGKINVKFHTKGDVLS—ETVRDQAKFKGLGLFKAIEGYVLAHPD
	5.1vjh	STL-KGALSVKFDVKCPADKFFSAFVEDT------------------NR-------PFE---KNGKTEIVDLVKKTT-I-QSGSEIQKYFKTLKGSIAVTPIGGGSHVVWTFHFEKVHKDI----DDPHSIIDESVKYFKKLDEAILNF--
	6.4rehA	---LTGKLICQTGIKSDGDVFHELFGTRPHHVPNITPANIQGCDLHEGSVVIWNYSIDGNAMIAKEEIVAIDEDKSVTFKVVEGHLFEEFKSIVFSVHVDTKGEDNLVTWSIDYEKLNES----VKDPTSYLDFLLSVTRDIEAHHLP---
	7.1vjh	--TLKGALSVKFDVKCPADKFFSAFVEDT----------------NR----------PFEKNGKTEIEAVDLVKKTT-I-QSGSEIQKYFKTLKGSIAVTPIGGGSHVVWTFHFEKVHKD----IDDPHSIIDESVKYFKKLDEAILNF—
	8.4jhgA	MG--VITSESEYVSSLSAEKLYRGIVEDNIIYPKALPRFIEKATLEGDGIKKLTFVGDFGSTKQHID-MVDRENCAYTYSVYEGIALSPLEKIVFEFKLVTPEEGCIVKSTTKYYTKGDDIELSKDYLEAGIERFEGFTKAVESFLLANPD
	9.7mlcA	S---QCSSTLVKHIKAPLHLVWSIVRRFDE-PQKYKPFISRCVVQGKKLVREVDLKSGLPATKSTEVEILDDNEHILGIRIVGGDHRLKNYSSTISLHSETIDGKTGTLAIESFVVDVP----EGNTKEETCFFVEALIQCNLNSLADVME
	10.2i9yA	-SSLVGKLETDVEIKASADKFHHMFAGKPHHVSKASPGNIQGCDLHEGSIVFWNYVHDGE-AKVAKERIEEPDKNLITFRVIEGDLMKEYKSFLLTIQVTPKPGGSIVHWHLEYEKISEEVAH----PETLLQFCVEVSKEIDEHLLAEE-
Isoform X3		20 40 60 80 100 120 140
	Query	MAFVTATAGVQVLIKSLPDTFWTKIQDSANSFPEAAPNLYSSIAVSGTGVRKVEYGPDSQNIKTSSELITENSLGKLAYTVTAGDILQKYDVNNFQAVIIHPNSEKWVGWTWTYNYLPENKARALQLDAEIAVIAVKTLTELDNYIQNKST
	1.4rehA	--GLTGKLICQTGIKSDGDVFHELFGTRPHHVPNITPANIQGCDLHEEFVVIWNYSIDGNIAKEEIVAIDE-EDKSVTFKVVEGHLFEEFKSIVFSVHVDTKGEDNLVTWSIDYEKLNE----SVKDPTSYLDFLLSVTRDIEAHHLP---
	2.4igvA	---LSGKMVKQVEILSDGIVFYEIFRYRLYLISEMSPVNIQGVDLEGNWVIFFKYTIDGK-EKTAKDIVEDEETKSVTFKIVEGDLMELYKTFIIIVQVDTKGEHNSVTWTFHYEKL----KEDVEEPNTLMNFCIEITKDIETYHLK---
	3.7mlcA	---SQCSSTLVKHIKAPLHLVWSIVRRFDE-PQKYKPF-ISRCVVQGKSVREVDLKSGLPTKSTEVLEILDDNEHILGIRIVGGDHLKNY-SSTISLHSETIDGGTLAIESFVVDVPEG--NTKEETCFFVEALIQCNLNSLADVTERLQA
	4.1icx	---GIFAFENEQSSTVAPAKLYKATKDS-DEIPKVIEPI-QSVEINGGTIKKIIIHDGH--TSFVLHKLDADEALTYNYSIIGGEGLESLE--KISYESILPGPGSIGKINVKFHTKGDVLS--ETVRDQAKFKGLGLFKAIEGYVLAHPD
	5.1vjh	STL-KGALSVKFDVKCPADKFFSAFVEDT------------------NR----------PFEKNGKTEIEDLVKKTT-I-QSGSEIQKYFKTLKGSIAVTPIGVGSHVVWTFHFEKVHKD----IDDPHSIIDESVKYFKKLDEAILNF--
	6.4rehA	---LTGKLICQTGIKSDGDVFHELFGTRPHHVPNITPANIQGCDLHEGSVVIWNYSIDGNAMIAKEEIVADEEDKSVTFKVVEGHLFEEFKSIVFSVHVDTKGEDNLVTWSIDYEKLNES----VKDPTSYLDFLLSVTRDIEAHHLP---
	7.1vjh	ST-LKGALSVKFDVKCPADKFFSAFVEDT----------------NR----------PFEKNGKTEIEAVDLVKKTT-I-QSGSEIQKYFKTLKGSIAVTPIGDGSHVVWTFHFEKVHKD----IDDPHSIIDESVKYFKKLDEAILNF--
	8.4c9cA	MAGV-YTYENEFTSDIPAPKLFKAFVDADNLIPKIAPQAVKCAELEGDGIKKITFGY---GYVKHKIHSIDKVNHTYSYSLIEGDALENIEKIDYETKLVAPHGGTIIKTTSKYHTKGDVEIKEEHVKAG-KEKAAHLFKLIEGYLKDHPS
	9.4m9bA	-G--VFTFEDEITSTVPPAKLYNAMKDADSITPKIIDDVKSVEIVEGNTIKKLTIVEDG-ETKFILHKVES-ANYAYNYSVVGGVALPTAEKITFETKLVEPNGGSIGKLTLKYHTKGDAKPDEEEL-KKGKAKGEGLFRAIEGYVLANPT
	10.7mlcA	S---QCSSTLVKHIKAPLHLVWSIVRRFDEP--QKYKPFISRCVVQGKKVREVDLKSGLP-ATKSTEVLEDDNEHILGIRIVGGDHRLKNYSSTISLHSETIDGKTGTLAIESFVVDVP----EGNTKEETCFFVEALIQCNLNSLADVME

All the residues are colored in black; however, those residues in template which are identical to the residue in the query sequence are highlighted in color. Coloring scheme is based on the property of amino acids, where polar are brightly coloured while non-polar residues are colored in dark shade.

Supplementary Table 5:

The top 5 final models of Uncharacterized protein LOC110648447 isoforms predicted by I-TASSER server

For each target, I-TASSER simulations generate a large ensemble of structural conformations, called decoys. To select the final models, I-TASSER uses the SPICKER program to cluster all the decoys based on the pair-wise structure similarity, and reports up to five models which corresponds to the five largest structure clusters. The confidence of each model is quantitatively measured by C-score that is calculated based on the significance of threading template alignments and the convergence parameters of the structure assembly simulations. C-score is typically in the range of [-5, 2], where a C-score of a higher value signifies a model with a higher confidence and vice-versa. TM-score and RMSD are estimated based on C-score and protein length following the correlation observed between these qualities. Since the top 5 models are ranked by the cluster size, it is possible that the lower-rank models have a higher C-score in rare cases. Although the first model has a better quality in most cases, it is also possible that the lower-rank models have a better quality than the higher-rank models as seen in our benchmark tests. If the I-TASSER simulations converge, it is possible to have less than 5 clusters generated; this is usually an indication that the models have a good quality because of the converged simulations.

**Isoform X1**					
Rank	1	2	3	4	5
Model	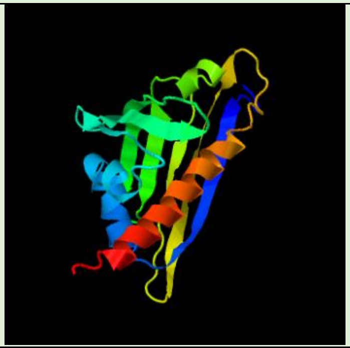	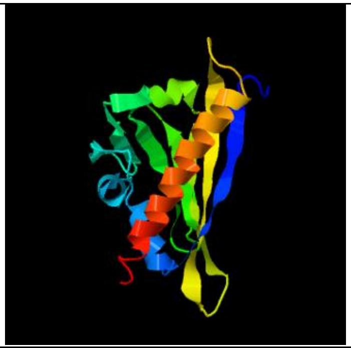	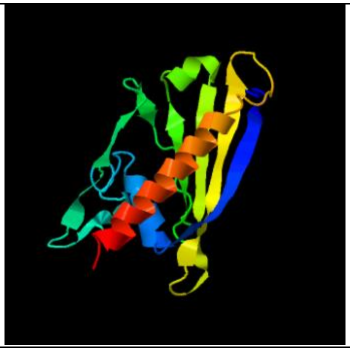	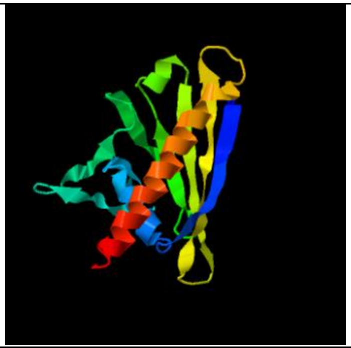	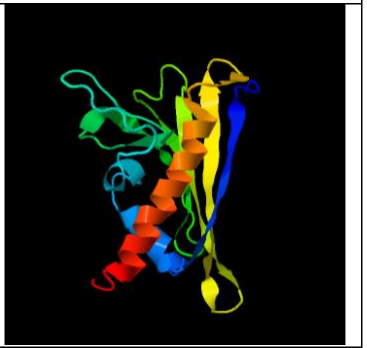
C-score	0.14	-2.27	-4.94	-5	-5
Note	Estimated TM-score = 0.73±0.11<br>Estimated RMSD = 4.5±3.0Å				

**Isoform X2**					
Rank	1	2	3	4	5
Model	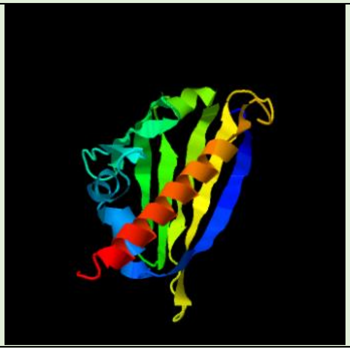	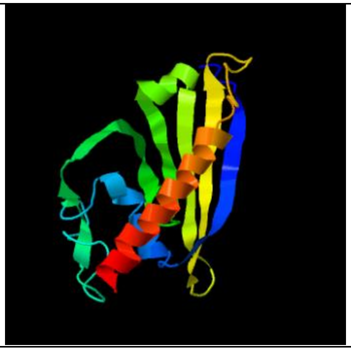	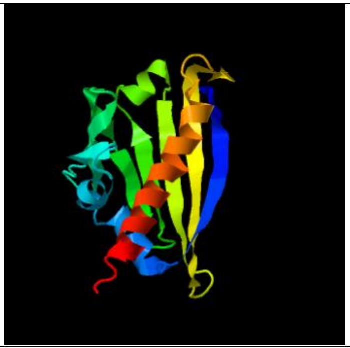	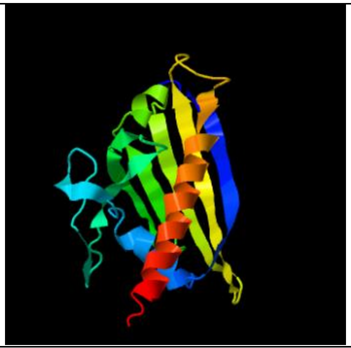	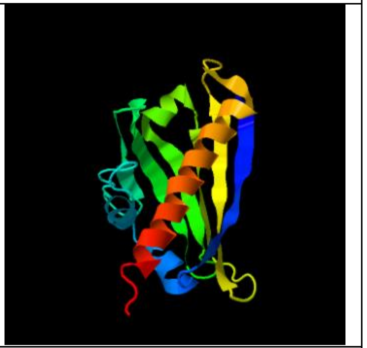
C-score	0.43	-5	-2.58	-5	-1.97
Note	Estimated TM-score = 0.77±0.10				

	Estimated RMSD = 3.9±2.7Å				
**Isoform X3**					
Rank	1	2	3	4	5
Model	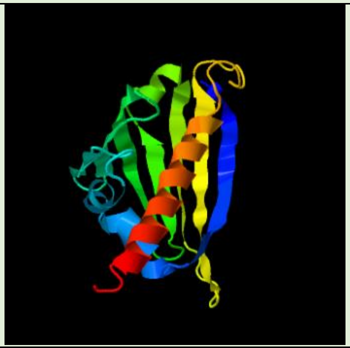	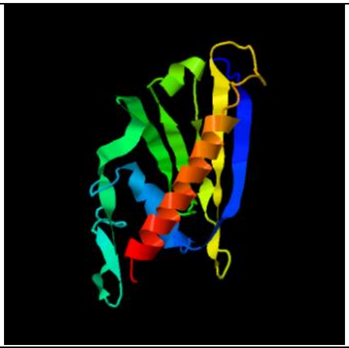	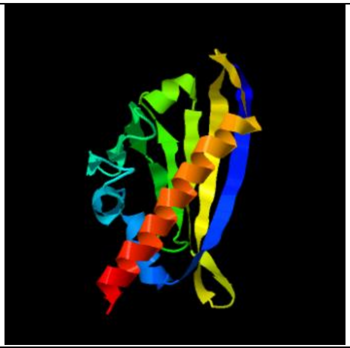	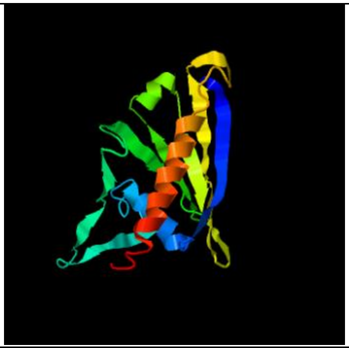	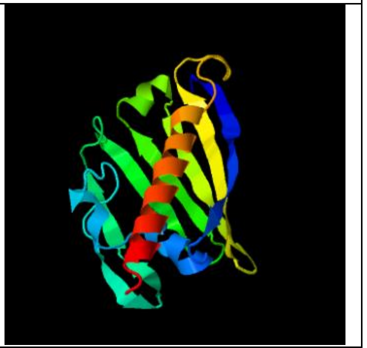
C-score	0.31	-5	-2.4	-5	-5
Note	Estimated TM-score = 0.75±0.10<br>Estimated RMSD = 4.1±2.8Å				

Supplementary Table 6:

The top 10 structurally closed proteins in the PDB to the model of Uncharacterized protein LOC110648447 isoforms predicted by I-TASSER server

After the structure assembly simulation, I-TASSER uses the TM-align structural alignment program to match the first I-TASSER model to all structures in the PDB library. This section reports the top 10 proteins from the PDB that have the closest structural similarity, i.e. the highest TM-score, to the predicted I-TASSER model. Due to the structural similarity, these proteins often have similar function to the target. However, users are encouraged to use the data in the next section 'Predicted function using COACH' to infer the function of the target protein, since COACH has been extensively trained to derive biological functions from multi-source of sequence and structure features which has on average a higher accuracy than the function annotations derived only from the global structure comparison.

	Rank	PDB hit	Name	TM-score	RMSD	IDEN	Coverage
Isoform X1	1	4igvA	Crystal structure of kirola (Act d 11)	0.866	1.54	0.113	0.940
	2	4rehA	Crystal structure of ginseng major latex-like protein 151 (GLP) from *Panax ginseng* (crystal-1)	0.864	1.48	0.120	0.940
	3	6ka2A	Crystal structure of a Thebaine synthase from *Papaver somniferum* in complex with TBN	0.863	1.64	0.090	0.954
	4	1e09A	Solution structure of the major cherry allergen Pru av 1	0.830	2.32	0.122	0.980
	5	1fm4A	Crystal structure of the birch pollen allergen Bet v 1L	0.828	2.29	0.142	0.980
	6	2qimA	Crystal structure of pathogenesis-related protein LIPR-10.2B from yellow lupine complex with cytokinin	0.811	2.54	0.116	0.974
	7	3rwsA	Crystal structure of *Medicago truncatula* Nodulin 13 (MtN13) in complex2 with trans-zeatin	0.806	2.54	0.108	0.980
	8	2flhD	Crystal structure of cytokinin-specific binding protein from mung bean in complex with cytokinin	0.805	2.49	0.103	0.954
	9	1tw0A	Native crystal structure of SPE16	0.802	2.53	0.074	0.980
	10	2bk0A	Crystal structure of the major celery allergen Api G 1	0.797	2.48	0.137	0.967
Isoform X2	1	4rehA	Crystal structure of ginseng major latex-like protein 151 (GLP) from *Panax ginseng* (crystal-1)	0.894	1.19	0.148	0.940
	2	4igvA	Crystal structure of kirola (Act d 11)	0.890	1.35	0.134	0.940
	3	6ka2A	Crystal structure of a Thebaine synthase from *Papaver somniferum* in complex with TBN	0.879	1.79	0.105	0.947
	4	1fskG	Complex formation between a FAB fragment of a monoclonal IgG antibody and the major allergen from birch pollen Bet v 1	0.838	2.19	0.129	0.974
	5	3c0vD	Crystal structure of cytokinin-specific binding protein in complex with cytokinin and Ta6Br12	0.835	2.15	0.113	0.940
	6	3rwsA	Crystal structure of *Medicago truncatula* Nodulin 13 (MtN13) in complex2 with trans-zeatin	0.834	2.22	0.102	0.974
	7	1e09A	Solution structure of the major cherry allergen Pru av 1	0.829	2.24	0.143	0.974
	8	1tw0B	Native crystal structure of SPE16	0.827	2.18	0.075	0.974
	9	4xrwA	Crystal structure of the di-domain ARO/CYC BexL from the BE-7585A biosynthetic pathway	0.825	2.18	0.090	0.954
	10	2qimA	Crystal structure of pathogenesis-related protein LIPR-10.2B from yellow lupine complex with cytokinin	0.825	2.27	0.110	0.967
Isoform X3	1	4rehA	Crystal structure of ginseng major latex-like protein 151 (GLP) from *Panax ginseng* (crystal-1)	0.896	1.18	0.141	0.940
	2	4igvA	Crystal structure of kirola (Act d 11)	0.893	1.28	0.134	0.940
	3	6ka2A	Crystal structure of a Thebaine synthase from *Papaver somniferum* in complex with TBN	0.883	1.79	0.098	0.947
	4	1fm4A	Crystal structure of the birch pollen allergen Bet v 1L	0.839	2.22	0.129	0.974
	5	3rwsA	Crystal structure of *Medicago truncatula* Nodulin 13 (MtN13) in complex2 with trans-zeatin	0.836	2.21	0.102	0.974
	6	2qimA	Crystal structure of pathogenesis-related protein LIPR-10.2B from yellow lupine complex with cytokinin	0.830	2.23	0.096	0.967
	7	1e09A	Solution structure of the major cherry allergen Pru av 1	0.829	2.26	0.129	0.974
	8	1tw0A	Native crystal structure of SPE16	0.823	2.30	0.068	0.974
	9	2flhD	Crystal structure of cytokinin-specific binding protein from mung bean in complex with cytokinin	0.822	2.31	0.112	0.940
	10	4xrwA	Crystal structure of the di-domain ARO/CYC BexL from the BE-7585A biosynthetic pathway	0.821	2.23	0.104	0.954

Ranking of proteins is based on TM-score of the structural alignment between the query structure and known structures in the PDB library.
RMSDa is the RMSD between residues that are structurally aligned by TM-align.
IDENa is the percentage sequence identity in the structurally aligned region.
Cov represents the coverage of the alignment by TM-align and is equal to the number of structurally aligned residues divided by length of the query protein.

Supplementary Table 7:

The predicted functions of Uncharacterized protein LOC110648447 isoforms by COFACTOR and COACH through I-TASSER server

This section reports biological annotations of the target protein by COFACTOR and COACH based on the I-TASSER structure prediction. While COFACTOR deduces protein functions (ligand-binding sites, EC and GO) using structure comparison and protein-protein networks, COACH is a meta-server approach that combines multiple function annotation results (on ligand-binding sites) from the COFACTOR, TM-SITE and S-SITE programs.

Supplementary Table 7.1: Ligand binding sites

Rank	C-score	Cluster size	PDB hit	PDB hit name	Ligand	Ligand name	Ligand binding site residues
**Isoform X1**							
1	0.15	30	4a83A	Crystal structure of major birch allergen Bet v 1 a in complex with deoxycholate	DXC	Deoxycholate	25, 26, 29, 32, 51, 63, 65, 77, 79, 95, 131, 134, 138
2	0.15	40	3zvuA	Structure of the PYR1 His60Pro mutant in complex with the HAB1 phosphatase and abscisic acid	A8S	Abscisic acid	39, 55, 63, 79, 81, 86, 93, 130, 134, 138
3	0.09	33	4n3eB	Crystal structure of Hyp-1, a St John's wort PR-10 protein, in complex with 8-anilo-1-napthalene sulfonate (ANS)	2AN	8-anilo-1-napthalene sulfonate (ANS)	10, 12, 14, 21, 25, 97, 109, 111, 135, 136, 139
4	0.06	14	1fm4A	Crystal structure of the birch pollen allergen Bet v 1L	DXC	Deoxycholate	25, 32, 53, 65, 79, 93, 95, 113, 115, 131, 138
5	0.04	10	4q0kA	Crystal structure of phytohormone binding protein from Medicago truncatula in complex with gibberellic acid (GA3)	GA3	Gibberellin	25, 32, 36, 53, 55, 63, 97, 134, 135, 138
**Isoform X2**							
**Rank**	**C-score**	**Cluster size**	**PDB hit**	**PDB hit name**	**Ligand**	**Ligand name**	**Ligand binding site residues**
1	0.24	62	3ie5A	Crystal structure of Hyp-1 protein from *Hypericum perforatum* (St John's wort) involved in hypericin biosynthesis	PG4	Tetraethylene glycol	25, 40, 86, 114, 138
2	0.18	34	4a83A	Crystal structure of major birch allergen Bet v 1 a in complex with deoxycholate	DXC	Deoxycholate	25, 26, 29, 30, 32, 51, 64, 66, 77, 79, 95, 131, 134, 138
3	0.08	31	4n3eB	Crystal structure of Hyp-1, a St John's wort PR-10 protein, in complex with 8-anilo-1-napthalene sulfonate (ANS)	2AN	8-anilo-1-napthalene sulfonate (ANS)	10, 12, 14, 21, 25, 97, 108, 110, 135, 136, 139
4	0.07	17	4a8gA	Crystal structure of major birch pollen allergen Bet v 1 a in complex with dimethylbenzylammonium propane sulfonate	DMX	Dimethylbenzylammonium propane sulfonate	25, 26, 30, 32, 51, 77, 79, 97, 110, 135
5	0.05	13	2wqlC	Crystal structure of the major carrot allergen Dau c 1	P4C	O-acetaldehydyl-hexaethelene glycol	25, 40, 77, 97, 110, 135, 138
**Isoform X3**							
**Rank**	**C-score**	**Cluster size**	**PDB hit**	**PDB hit name**	**Ligand**	**Ligand name**	**Ligand binding site residues**
1	0.26	65	3ie5A	Crystal structure of Hyp-1 protein from *Hypericum perforatum* (St John's wort) involved in hypericin biosynthesis	PG4	Tetraethylene glycol	25, 40, 86, 114, 138
2	0.19	32	4a83A	Crystal structure of major birch allergen Bet v 1 a in complex with deoxycholate	DXC	Deoxycholate	25, 26, 29, 30, 32, 51, 64, 66, 77, 79, 95, 131, 134, 138
3	0.14	32	4n3eF	Crystal structure of Hyp-1, a St John's wort PR-10 protein, in complex with 8-anilo-1-napthalene sulfonate (ANS)	2AN	8-anilo-1-napthalene sulfonate (ANS)	12, 21, 25, 29, 79, 97, 99, 110, 135, 136, 139
4	0.04	10	4c94E	Crystal structure of the strawberry pathogenesis-related 10 (PR-10) Fra a 3 protein in complex with catechin	KXN	Catechin	29, 39, 53, 55, 66, 134, 138

5	0.04	9	4a81A	Crystal structure of major birch pollen allergen Bet v 1 a in ternary complex with 8-anilonapthalene-1-sulfonate (ANS) and deoxycholic acid	2AN	8-anilo-1-napthalene sulfonate (ANS)	10, 12, 25, 79, 97, 110, 112, 128, 131, 132, 135, 136

Note:
C-score is the confidence score of the prediction. C-score ranges [0-1], where a higher score indicates a more reliable prediction.
Cluster size is the total number of templates in a cluster.
Lig Name is name of possible binding ligand. Click the name to view its information in the BioLiP database
Rep is a single complex structure with the most representative ligand in the cluster, i.e., the one listed in the Lig Name column.
Mult is the complex structures with all potential binding ligands in the cluster

Supplementary Table 7.2: Enzyme commission (EC) numbers and active sites

Isoform X1

Rank	C-score[EC]	PDB hit	PDB hit name	TM-score	RMSD	IDEN	Cov	EC number	EC name	Active site residues
1	0.386	2vq5A	X-ray structure of Norcoclaurine synthase from *Thalictrum flavum* in complex with dopamine and hydroxybenzaldehyde	0.741	3.03	0.101	0.980	4.2.1.78	Norcoclaurine synthase	49, 68
2	0.252	2de6B	The reduced complex between oxygenase and ferredoxin in carbazole 1,9a-dioxygenase	0.607	3.89	0.056	0.874	1.14.12.-	Dioxygenases	n/a
3	0.236	1wqlA	Cumene dioxygenase (cumA1A2) from *Pseudomonas fluorescens* IP01	0.617	3.74	0.044	0.887	1.14.12.-	Dioxygenases	n/a
4	0.234	1im0A	Outer membrane phospholipase A from *E. coli* N156A active site mutant pH 8.3	0.533	4.47	0.053	0.834	3.1.1.32<br>3.1.1.4	PhospholipaseA1<br>PhospholipaseA2	n/a
5	0.234	1z01A	2-Oxoquinoline 8-monooxygenase component: Active site modulation by Rieske-[2fe-2S] center oxidation/reduction	0.609	3.72	0.035	0.868	1.14.13.61	2-hydroxyquinoline 8-monooxygenase	27

Isoform X2

Rank	C-score[EC]	PDB hit	PDB hit name	TM-score	RMSD	IDEN	Cov	EC number	EC name	Active site residues
1	0.349	2vq5A	X-ray structure of Norcoclaurine synthase from *Thalictrum flavum* in complex with dopamine and hydroxybenzaldehyde	0.788	2.55	0.103	0.967	4.2.1.78	Norcoclaurine synthase	45, 49
2	0.289	1uliC	Biphenyl dioxygenase (BphA1A2) derived from *Rhodococcus sp.* strain RHA1	0.644	3.54	0.059	0.894	1.14.12.18	Biphenyl 2,3-dioxygenase	n/a
3	0.289	3en1A	Crystal structure of Toluene 2,3-dioxygenase	0.650	3.53	0.066	0.901	1.14.12.11	Toluene dioxygenase	n/a
4	0.288	2de6B	The reduced complex between oxygenase and ferredoxin in carbazole 1,9a-dioxygenase	0.653	3.78	0.064	0.907	1.14.12.-	Dioxygenases	n/a
5	0.287	1wqlA	Cumene dioxygenase (cumA1A2) from *Pseudomonas fluorescens* IP01	0.639	3.63	0.074	0.901	1.14.12.-	Dioxygenases	n/a

Isoform X3

Rank	C-score[EC]	PDB hit	PDB hit name	TM-score	RMSD	IDEN	Cov	EC number	EC name	Active site residues
1	0.415	2vq5A	X-ray structure of Norcoclaurine synthase from *Thalictrum flavum* in complex with dopamine and hydroxybenzaldehyde	0.780	2.68	0.096	0.967	4.2.1.78	Norcoclaurine synthase	49
2	0.281	1ndoA	Naphthalene 1,2-dioxygenase	0.639	3.70	0.110	0.901	1.14.12.12	Naphthalene 1,2-dioxygenase	n/a

3	0.274	2de6B	The reduced complex between oxygenase and ferredoxin in carbazole 1,9a-dioxygenase	0.646	3.89	0.064	0.907	1.14.12.-	Dioxygenases	n/a
4	0.236	2fy3A	Structures of ligand bound human choline acetyltransferase provides insight into regulation of acetylcholine synthesis	0.520	4.74	0.044	0.848	2.3.1.6	Choline O-acetyltransferase	n/a
5	0.234	1t7nA	Crystal structure of the M564G mutant of murine CrAT	0.523	4.63	0.074	0.854	2.3.1.7	Carnitine O-acetyltransferase	n/a

$Cscore^{EC}$ is the confidence score for the EC number prediction. $Cscore^{EC}$ values range in between [0-1]; where a higher score indicates a more reliable EC number prediction.
TM-score is a measure of global structural similarity between query and template protein.
RMSDa is the RMSD between residues that are structurally aligned by TM-align.
IDENa is the percentage sequence identity in the structurally aligned region.

Supplementary Table 7.3: Consensus prediction of Gene ontology (GO) terms

The table shows a consensus GO terms amongst the 10 top scoring templates. The GO-score associated with each prediction is defined as the average weight of the GO term, where the weights are assigned based on $Cscore^{GO}$ of the template

	Molecular function		Biological process		Cellular component	
	GO term	GO score	GO term	GO score	GO term	GO score
Isoform X1	GO:0010427 ABA binding<br>GO:0004872 Signaling receptor activity<br>GO:0042803 Protein homodimerization activity	0.39<br>0.39<br>0.39	GO:0006952 Defense response<br>GO:0009607 Response to biotic stimulus<br>GO:0009738 ABA-activated signaling pathway	0.88<br>0.88<br>0.39	GO:0005737 Cytoplasm<br>GO:0005634 Nucleus	0.39<br>0.39
Isoform X2	GO:0016787 Hydrolase activity	0.42	GO:0006952 Defense response<br>GO:0009607 Response to biotic stimulus	0.94<br>0.94	None was predicted	
Isoform X3	GO:0004872 Signaling receptor activity<br>GO:0010427 ABA binding<br>GO:0042803 Protein homodimerization activity	0.39<br>0.39<br>0.39	GO:0006952 Defense response<br>GO:0009607 Response to biotic stimulus<br>GO:0009738 ABA-activated signaling pathway	0.88<br>0.88<br>0.39	GO:0005737 Cytoplasm<br>GO:0005634 Nucleus	0.64<br>0.39

Supplementary Table 7.4: Top 10 homologous GO term templates in PDB

Isoform X1								
**Rank**	**$Cscore^{GO}$**	**TM-score**	**RMSD[a]**	**IDEN[a]**	**Cov**	**PDB hit**	**Associated GO terms**	**GO term names**
1	0.42	0.7778	2.56	0.14	0.96	2wq1A	GO:0006952<br>GO:0009607	Defense response<br>Response to biotic stimulus
2	0.41	0.7887	2.64	0.10	0.97	3ie5B	GO:0006952<br>GO:0009607	Defense response<br>Response to biotic stimulus
3	0.41	0.7967	2.48	0.14	0.97	2bk0A	GO:0006952<br>GO:0009607	Defense response<br>Response to biotic stimulus
4	0.39	0.7631	2.83	0.12	0.97	3rt0C	GO:0005575<br>GO:0005634	Cellular component<br>Nucleus

							GO:0005515<br>GO:0010427<br>GO:0004872<br>GO:0042803<br>GO:0005737<br>GO:0009738	Protein binding<br>Abscisic acid binding<br>Signaling receptor activity<br>Protein homodimerization activity<br>Cytoplasm<br>Abscisic acid-activated signaling pathway
5	0.38	0.7472	3.07	0.12	0.97	2k7hA	GO:0006952<br>GO:0009607	Defense response<br>Response to biotic stimulus
6	0.36	0.6816	3.05	0.10	0.89	1z94B	GO:0006950	Response to stress
7	0.36	0.8304	2.32	0.12	0.98	1e09A	GO:0006952<br>GO:0009607	Defense response<br>Response to biotic stimulus
8	0.35	0.6723	2.03	0.14	0.77	1vjhA	GO:0006952<br>GO:0009607	Defense response<br>Response to biotic stimulus
9	0.34	0.8078	2.36	0.10	0.95	2flhB	GO:0006952<br>GO:0009607	Defense response<br>Response to biotic stimulus
10	0.34	0.8006	2.40	0.14	0.97	1b6fA	GO:0006952<br>GO:0009607<br>GO:0005737	Defense response<br>Response to biotic stimulus<br>Cytoplasm
**Isoform X2**								
**Rank**	**Cscore^{GO}**	**TM-score**	**RMSD^{a}**	**IDEN^{a}**	**Cov**	**PDB hit**	**Associated GO terms**	**GO term names**
1	0.45	0.8124	2.27	0.13	0.96	2bk0A	GO:0006952<br>GO:0009607	Defense response<br>Response to biotic stimulus
2	0.44	0.7987	2.56	0.12	0.97	3ie5B	GO:0006952<br>GO:0009607	Defense response<br>Response to biotic stimulus
3	0.44	0.7715	2.96	0.12	0.97	2k7hA	GO:0006952<br>GO:0009607	Defense response<br>Response to biotic stimulus
4	0.42	0.7978	2.48	0.07	0.97	1ifvA	GO:0006952<br>GO:0016787<br>GO:0009607	Defense response<br>Hydrolase activity<br>Response to biotic stimulus
5	0.42	0.7827	2.48	0.13	0.95	2wqlA	GO:0006952<br>GO:0009607	Defense response<br>Response to biotic stimulus
6	0.41	0.8107	2.34	0.11	0.96	1b6fA	GO:0006952<br>GO:0005737<br>GO:0009607	Defense response<br>Cytoplasm<br>Response to biotic stimulus
7	0.38	0.7721	2.78	0.07	0.96	1icxA	GO:0009607<br>GO:0006952	Response to biotic stimulus<br>Defense response
8	0.37	0.6762	1.90	0.16	0.76	1vjhA	GO:0009607<br>GO:0006952	Response to biotic stimulus<br>Defense response
9	0.37	0.8288	2.24	0.14	0.97	1e09A	GO:0009607<br>GO:0006952	Response to biotic stimulus<br>Defense response
10	0.36	0.8242	2.28	0.07	0.97	1tw0A	GO:0009607<br>GO:0006952	Response to biotic stimulus<br>Defense response
**Isoform X3**								
**Rank**	**Cscore^{GO}**	**TM-score**	**RMSD^{a}**	**IDEN^{a}**	**Cov**	**PDB hit**	**Associated GO terms**	**GO term names**
1	0.44	0.8152	2.28	0.12	0.96	2bk0A	GO:0006952	Defense response

							GO:0009607	Response to biotic stimulus
2	0.42	0.8110	2.50	0.12	0.97	1b6fA	GO:0006952<br>GO:0005737<br>GO:0009607	Defense response<br>Cytoplasm<br>Response to biotic stimulus
3	0.41	0.7883	2.47	0.14	0.95	2wqlA	GO:0006952<br>GO:0009607	Defense response<br>Response to biotic stimulus
4	0.39	0.7745	2.69	0.12	0.95	3rt0C	GO:0005575<br>GO:0005634<br>GO:0005515<br>GO:0010427<br>GO:0004872<br>GO:0042803<br>GO:0005737<br>GO:0009738	Cellular component<br>Nucleus<br>Protein binding<br>Abscisic acid binding<br>Signaling receptor activity<br>Protein homodimerization activity<br>Cytoplasm<br>Abscisic acid-activated signaling pathway
5	0.37	0.7656	2.85	0.07	0.96	1icxA	GO:0006952<br>GO:0009607	Defense response<br>Response to biotic stimulus
6	0.36	0.6730	1.93	0.16	0.76	1vjhA	GO:0006952<br>GO:0009607	Defense response<br>Response to biotic stimulus
7	0.36	0.8285	2.26	0.13	0.97	1e09A	GO:0006952<br>GO:0009607	Defense response<br>Response to biotic stimulus
8	0.35	0.8227	2.30	0.07	0.97	1tw0A	GO:0006952<br>GO:0009607	Defense response<br>Response to biotic stimulus
9	0.35	0.8304	2.26	0.10	0.95	2flhB	GO:0006952<br>GO:0009607	Defense response<br>Response to biotic stimulus
10	0.34	0.7934	2.62	0.12	0.97	3ie5B	GO:0006952<br>GO:0009607	Defense response<br>Response to biotic stimulus
